# Synthesis of spiro[indoline-3,1$^\prime$-quinolizines] and spiro[indoline-3,4$^\prime$-pyrido[1,2-a]quinolines] via three-component reactions of azaarenes, acetylenedicarboxylate, and 3-methyleneoxindoles

**DOI:** 10.1007/s11030-013-9459-5

**Published:** 2013-07-19

**Authors:** Jing Sun, Hui Gong, Yan Sun, Chao-Guo Yan

**Affiliations:** College of Chemistry & Chemical Engineering, Yangzhou University, Yangzhou, 225002 China

**Keywords:** Multicomponent reaction, MCR, Domino reaction, Diels–Alder reaction, Spirooxindole, Isoquinolinuclidine, Spiro[indoline-3$$, $$1$$^\prime $$-quinolizines]

## Abstract

**Abstract:**

The three-component reactions of substituted pyridines, dimethyl acetylenedicarboxylates, and 3-phenacylideneoxindoles afforded spiro[indoline-3,1$^\prime$-quinolizines] in high yields and with high diastereoselectivity. The Diels–Alder reactions of spiro[indoline-3,1$^\prime$-quinolizines] with maleic anhydride and $N$-phenyl maleimides successfully resulted in polyfunctionalized isoquinolinuclidine derivatives. The similar three-component reactions with quinoline resulted in the novel spiro[indoline-3,4$^\prime$-pyrido[1,2-a]quinolines] in moderate to good yields.

**Graphical Abstract:**

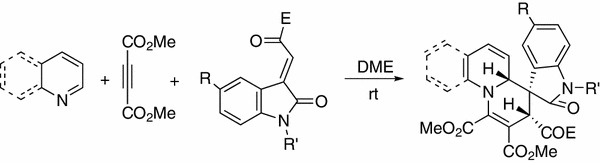

**Electronic supplementary material:**

The online version of this article (doi:10.1007/s11030-013-9459-5) contains supplementary material, which is available to authorized users.

## Introduction

The spirooxindole core is a privileged heterocyclic ring system that is featured in a large number of bioactive naturally occurring alkaloids and medicinally relevant compounds [1–5]. Due to the exceptional high reactivity of the 3-carbonyl group, 3-methylene and 3-phenacylideneoxindoles have attracted a lot of attention for synthetic reactions, especially multicomponent reactions [6–8] and catalytic asymmetric reactions [9–11] in the past few years. As a result, numerous elegant transformations have been developed for the diastereoselective and enantioselective construction of versatile spirooxindole skeletons [12–17]. For the synthesis of these challenging heterocycles, the 1,4-dipolar cycloaddition of Huisgen 1,4-dipoles, which were generated from reactions of nitrogen heterocycles with electron-deficient alkynes, has proven to be a convenient and efficient synthetic methodology [18, 19]. Nair et al. [20] first reported the three-component reaction of pyridine, dimethyl acetylenedicarboxylate (DMAD), and $N$-benzylisatins to give spiro[indololine-3,2$^\prime$-pyrido[2,1-b][1,3]oxazine]. Later, Yavari [21] and Nair [22] reported the similar reactions of quinoline and isoquinoline with DMAD and isatins for the preparation of complex spirooxindole derivatives. Shi and co-workers [23] found that the three-component reactions of pyridine, DMAD, and $N$-substituted isatylidene derivatives afforded spiro[indoline-3,2-quinolizine] in high yields and with good diastereoselectivities. Recently, we successfully developed an efficient synthetic protocol for dispirooxindole-fused heterocycles via the domino reaction of $p$-dimethylaminopyridine and DMAD with two molecules of 3-phenacylideneoxindoles [24]. In order to demonstrate the synthetic utility of this practical method, herein we wish to report the three-component reaction of azaarenes such as substituted pyridines and quinoline with DMAD and 3-phenacylideneoxindoles and 3-ethoxycarbonylmethyleneoxindoles for the synthesis of spiro [indoline-3,1$^\prime$-quinolizine] derivatives and their potential applications as effective dienes for Diels–Alder reactions.

## Results and discussion

We initiated our studies by evaluating the reactivity of the Huisgen 1,4-dipoles generated from the reaction of alkylpyridines with DMAD. According to our previously established conditions for the reaction of 4-dimethylaminopyridine [24], the three-component reactions of 2-picoline with DMAD and 3-phenacylideneoxindoles in THF at room temperature proceeded very smoothly to give the expected 2$^\prime$,9a$^\prime$-dihydrospiro[indoline-3,1$^\prime$-quinolizine] **1a**–**1b **in moderate yields (Table 1, entries 1–2). Under similar conditions, the reactions with 3-picoline and 4-picoline gave the corresponding spiro products **1c**–**1h** in high yields (Table 1, entries 3–8). When 4-methoxypyridine was utilized in the reactions, much higher yields of spiro compounds **1j**–**1o** (Table 1, entries 9–15) were obtained.

**Table 1:** Synthesis of 2$^\prime$,9a$^\prime$-dihydrospiro[indoline-3,1$^\prime$-quinolizine]s **1a**–**1o**

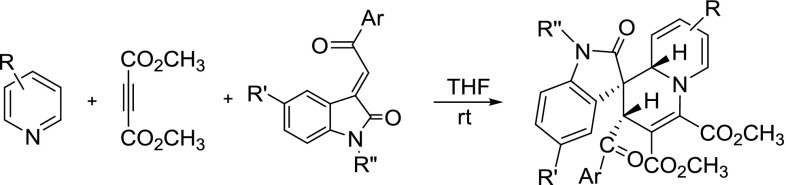						
**1a**–**1o**						
Entry	Compd.	R	Ar	R$^\prime$	R${^{\prime \prime }}$	Yield$^{\mathrm{{a}}}$ (%)
1	**1a**	2-CH$_{3}$	$p$-CH$_{3}$C$_{6}$H$_{4}$	H	Bn	66
2	**1b**	2-CH$_{3}$	$p$-CH$_{3}$C$_{6}$H$_{4}$	CH$_{3}$	*n*-C$_{4}$H$_{9}$	53
3	**1c**	3-CH$_{3}$	$p$-CH$_{3}$OC$_{6}$H$_{4}$	H	Bn	61
4	**1d**	3-CH$_{3}$	$m$-CH$_{3}$OC$_{6}$H$_{4}$	F	Bn	58
5	**1e**	3-CH$_{3}$	$p$-CH$_{3}$C$_{6}$H$_{4}$	Cl	Bn	62
6	**1f**	3-CH$_{3}$	$p$-CH$_{3}$OC$_{6}$H$_{4}$	F	*n*-C$_{4}$H$_{9}$	74
7	**1g**	4-CH$_{3}$	$p$-CH$_{3}$OC$_{6}$H$_{4}$	F	*n*-C$_{4}$H$_{9}$	77
8	**1h**	4-CH$_{3}$	$p$-CH$_{3}$OC$_{6}$H$_{4}$	Cl	*n*-C$_{4}$H$_{9}$	75
9	**1i**	4-CH$_{3}$O	$p$-CH$_{3}$OC$_{6}$H$_{4}$	Cl	Bn	89
10	**1j**	4-CH$_{3}$O	$p$-CH$_{3}$C$_{6}$H$_{4}$	Cl	Bn	84
11	**1k**	4-CH$_{3}$O	$p$-CH$_{3}$OC$_{6}$H$_{4}$	F	Bn	91
12	**1l**	4-CH$_{3}$O	$p$-CH$_{3}$C$_{6}$H$_{4}$	F	Bn	87
13	**1m**	4-CH$_{3}$O	C$_{6}$H$_{5}$	F	Bn	81
14	**1n**	4-CH$_{3}$O	$p$-CH$_{3}$OC$_{6}$H$_{4}$	Cl	*n*-C$_{4}$H$_{9}$	93
15	**1o**	4-CH$_{3}$O	$p$-CH$_{3}$C$_{6}$H$_{4}$	F	*n*-C$_{4}$H$_{9}$	90

*Reaction conditions* Substituted pyridine (1.2 mmol), DMAD (1.2 mmol) and 3-phenacylideneoxindole (1.0 mmol) in THF (10.0 mL), rt, 6 h

$$^{\mathrm{{a}}}$$Isolated yield

**Fig. 1:**
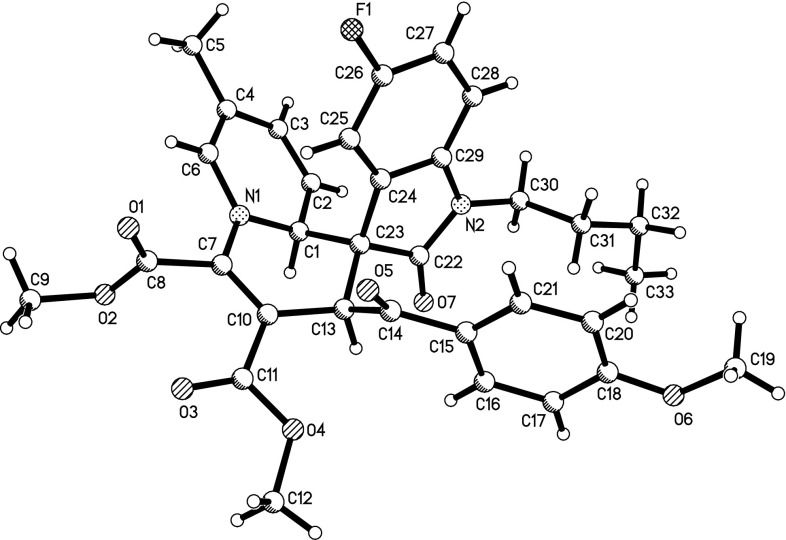
ORTEP representation of crystal structure of spiro compound **1f**

The structures of the prepared 2$^\prime$,9a$^\prime$-dihydrospiro[indoline-3,1$^\prime$-quinolizin]-2-ones **1a**–**1n** were fully characterized by $^{1}$H NMR, $^{13}$C NMR, HRMS, and IR. The $^{1}$H NMR spectra of the spiro compounds **1a**–**1n** usually show one set of signals for the characteristic groups in the molecule, which clearly indicated that only one diastereoisomer existed in each sample. The molecular structures of compounds **1f ** (Fig. 1), **1h **(SPI, Fig. s1), and **1m** (SPI, Fig. s2) were successfully confirmed by single-crystal X-ray diffraction. These three molecules (**1f**, **1h**, **1m)** have the same stereochemistry. In the newly formed tetrahydropyridyl ring, the two protons at 2- and 4-positions are in *cis*-orientation. The benzoyl and aryl groups of the oxindole moiety also exist in *cis*-position. It is reported that the benzoyl group and aryl group of oxindole moiety exist in *cis*-position in the starting 3-phenacylideneoxindoles [25, 26] indicating that this configuration is expected to be retained in the reaction. Thus, we unambiguously ascertained that compounds **1a**–**1o** are the *cis*-isomers proving that this three-component reaction undergoes with very high diastereoselectivity.

**Scheme 1:**
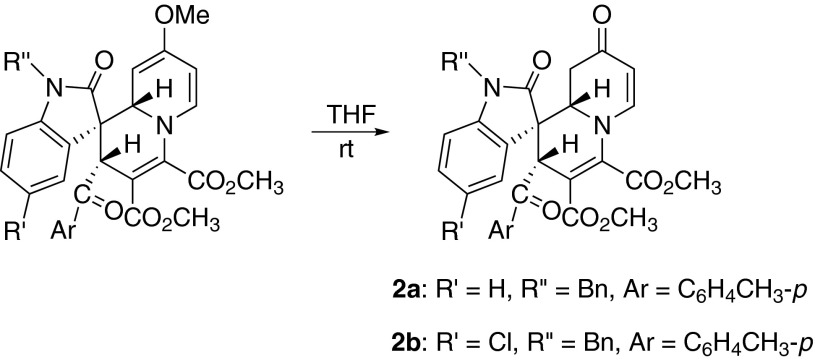
Formation of 2,8$^\prime$-dioxo-2$^\prime$,8$^\prime$,9$^\prime$,9a$^\prime$-tetrahydrospiro[indoline-3,1$^\prime$-quinolizines]

It should be pointed out that spiro compounds **1i**–**1o **derived from the reactions with 4-methoxypyridine are not very stable in solution because of the presence of a reactive methyl vinyl ether moiety. The 4-methoxy group could be slowly transformed into the 4-carbonyl group during the purification process when dissolved in THF, DCM, ethyl acetate, and toluene (Scheme 1). The structures of the two spiro compounds **2a**–**2b** were successfully characterized via spectroscopic methods, and the structure of spiro compound **2b** was also confirmed by X-ray diffraction (SPI, Fig. s3).

**Fig. 2:**
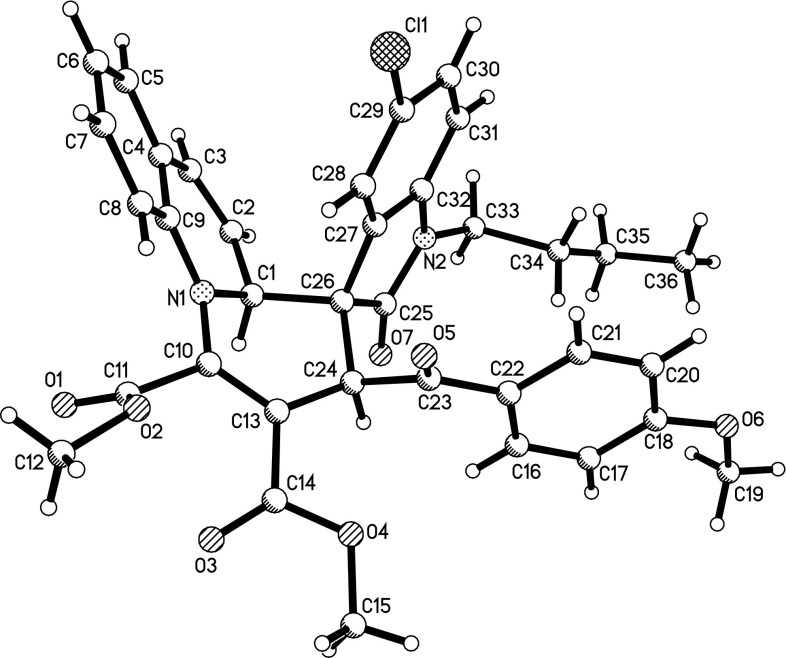
X-ray structure of spiro compound **3e**

**Fig. 3:**
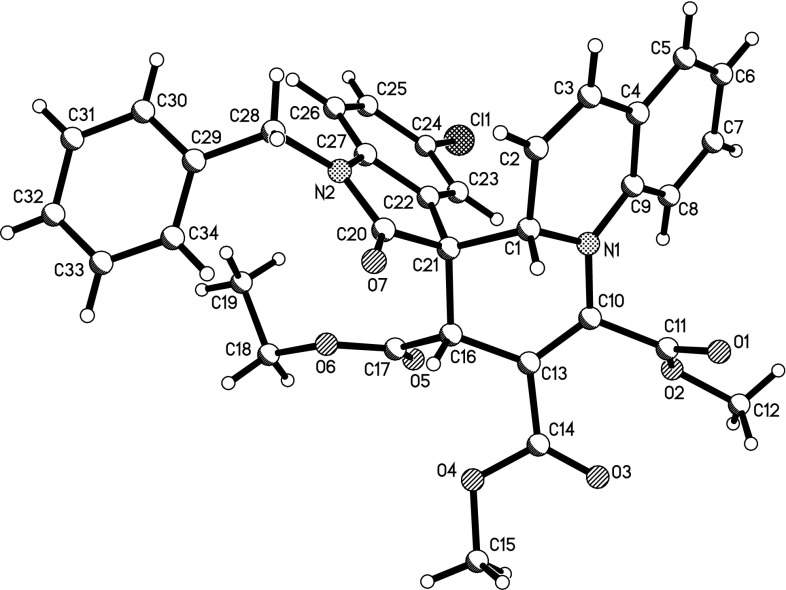
X-ray structure of spiro compound **3i**

To further demonstrate the substrate scope and the diastereoselectivity of this three-component reaction, quinoline was also utilized in the reaction. The three-component reaction of quinoline, DMAD, and 3-phenacylideneoxindoles in THF usually resulted in a complex mixture. After exploring different solvents, we were pleased to find that the reaction proceeded smoothly in DME to give the desired 3$^\prime$,4a$^\prime$-dihydrospiro[indoline-3,4$^\prime$-pyrido[1,2-a]quinolines] **3a**–**3e** in moderate yields after thin-layer chromatography (Table 2, entries 1–5). Under similar conditions, the reactions with 3-ethoxycarbonylmethyleneoxindoles afforded the spiro [indoline-3,4$^\prime$-pyrido[1,2-a]quinolines] **3f**–**3j ** with much better yields (Table 2, entries 6–10). The structures of spiro compounds **3a**–**3j **were also confirmed using spectroscopic methods and compounds **3e** and **3i** were further confirmed by X-ray diffraction (Figs. 2, 3, respectively). A stereochemistry similar to that of **1a**–**1o **was observed for the spiro compounds **3a**–**3j**, in which the two protons at 2- and 4-positions existed in *cis*-orientation in the newly formed tetrahydropyridyl ring, and the benzoyl group and the aryl group of the oxindole moiety also existed in *cis*-position. These results also indicate that this three-component reaction is a high diastereoselective reaction.

**Table 2:** Synthesis of 3$^\prime$,4a$^\prime$-dihydrospiro[indoline-3,4$^\prime$-pyrido[1,2-a]quinolines] **3a**–**3j**

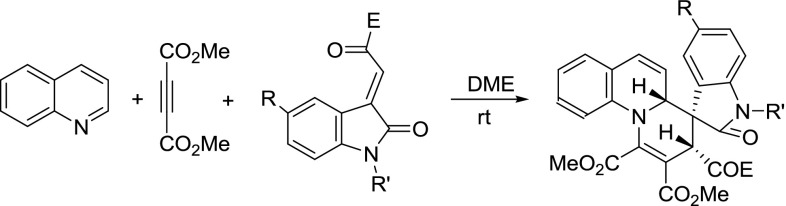					
**3a**–**3j**					
Entry	Compd.	R	R$^\prime$	E	Yield$^\mathrm{{a}}$ (%)
1	**3a**	Cl	Bn	C$_{6}$H$_{4}$CH$_{3}$-$p$	40
2	**3b**	Cl	Bn	C$_{6}$H$_{4}$Cl-$p$	52
3	**3c**	F	Bn	C$_{6}$H$_{5}$	55
4	**3d**	F	Bn	C$_{6}$H$_{4}$Cl-$p$	53
5	**3e**	Cl	*n*-C$_{4}$H$_{9}$	C$_{6}$H$_{4}$OCH$_{3}$-$p$	60
6	**3f**	CH$_{3}$	Bn	OEt	50
7	**3g**	H	Bn	OEt	63
8	**3h**	Cl	Bn	OEt	70
9	**3i**	Cl	*n*-Bu	OEt	73
10	**3j**	F	Bn	OEt	65

*Reaction conditions* Quinoline (1.5 mmol), DMAD (1.5 mmol), and 3-methyleneoxindole (1.0 mmol) in DME (10.0 mL), rt, 6 h $^\mathrm{{a}}$Isolated yield

**Fig. 4:**
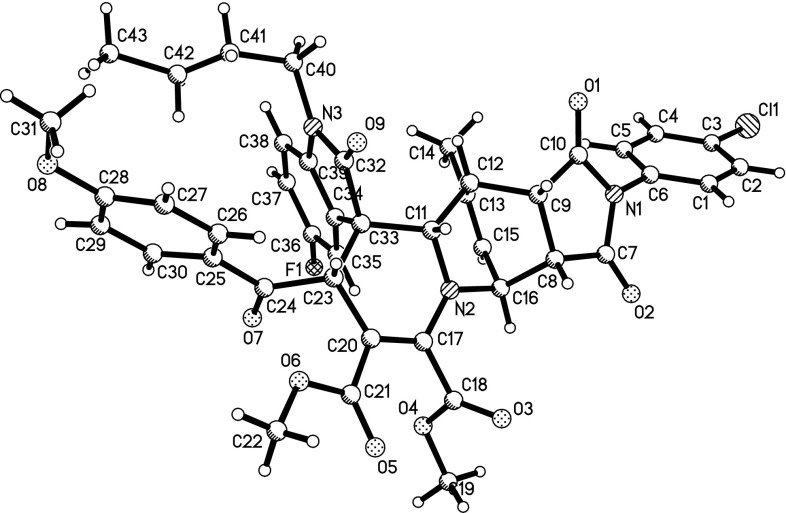
ORTEP representation of crystal structure of spiro compound **4d**

There is a 1,2-dihydropyridyl moiety in the above-prepared dihydrospiro[indoline-3,1$^\prime$-quinolizin]-2-ones **1a**–**1n**. 1,2-Dihydropyridine is an effective diene for Diels–Alder reaction to construct versatile bridged heterocyclic compounds [27–33]. Thus, we proceeded to investigate the role of our spiro compounds **1a**–**1n** as dienophiles in Diels–Alder reactions. The reaction of dihydrospiro[indoline-3,1$^\prime$-quinolizines] with a slight excess of $N$-phenyl maleimides or maleic anhydride proceeded smoothly in refluxing 1,2-dimethoxyethane for 6 h to give the desired 1,4-cycloaddition products **4a**–**4g **in satisfactory yields (Table 3). $^{1}$H NMR data and single-crystal determination of compound **4d** (Fig. 4) indicated that the configuration of previous dihydrospiro[indoline-3,1$^\prime$-quinolizine] moiety is retained and the maleimide unit exists in *exo*-configuration in this Diels–Alder reaction.

**Table 3:** Diels–Alder reactions of 2$^\prime$,9a$^\prime$-dihydrospiro[indoline-3,1$^\prime$-quinolizines]

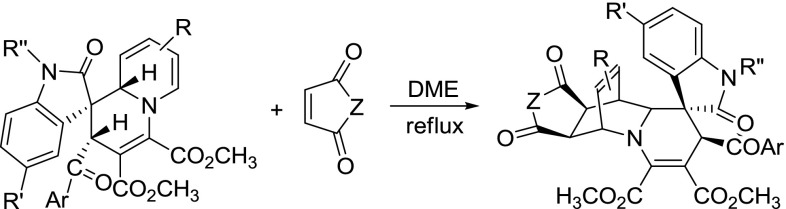							
**4a**–**4g**							
Entry	Compd.	R	Ar	R$^\prime$	R$^{\prime \prime }$	Z	Yield$^\mathrm{{a}}$ (%)
1	**4a**	4-CH$_{3}$	$p$-CH$_{3}$OC$_{6}$H$_{4}$	F	Bn	NC$_{6}$H$_{5}$	80
2	**4b**	4-CH$_{3}$	$p$-CH$_{3}$C$_{6}$H$_{4}$	Cl	Bn	NC$_{6}$H$_{5}$	77
3	**4c**	4-CH$_{3}$	$p$-CH$_{3}$OC$_{6}$H$_{4}$	F	*n*-C$_{4}$H$_{9}$	NC$_{6}$H$_{4}$CH$_{3}$-$p$	72
4	**4d**	4-CH$_{3}$	$p$-CH$_{3}$OC$_{6}$H$_{4}$	F	*n*-C$_{4}$H$_{9}$	NC$_{6}$H$_{4}$Cl-$p$	85
5	**4e**	3-CH$_{3}$	$p$-CH$_{3}$OC$_{6}$H$_{4}$	F	*n*-C$_{4}$H$_{9}$	NC$_{6}$H$_{4}$Cl-$p$	86
6	**4f**	4-OCH$_{3}$	$p$-CH$_{3}$OC$_{6}$H$_{4}$	Cl	*n*-C$_{4}$H$_{9}$	O	90
7	**4g**	4-OCH$_{3}$	$p$-CH$_{3}$C$_{6}$H$_{4}$	F	*n*-C$_{4}$H$_{9}$	O	88

*Reaction conditions* Spiro[indoline-3,1$^\prime$-quinolizine] (1.0 mmol) and $N$-substituted maleimide or maleic anhydride (1.5 mmol) in DME (10.0 mL), reflux, 12 h

$$^\mathrm{{a}}$$ Isolated yield

## Conclusion

In summary, an efficient protocol for the synthesis of functionalized spiro[indoline-3,1$^\prime$-quinolizine] and spiro[indoline-3,4$^\prime$-pyrido[1,2-a]quinoline] was successfully developed by three-component reactions of nitrogen heterocycles, DMADs, and 3-methyleneoxindoles. This MCR reaction can proceed smoothly under mild conditions to afford complex heterocycles in moderate to good yields and high diastereoselectivities. Furthermore, the prepared spiro[indoline-3,1$^\prime$-quinolizines] can undergo Diels–Alder reactions with maleic anhydride and $N$-phenyl maleimides to give complex isoquinolinuclidine derivatives. The simplicity of the procedure, readily available substrates, and ease of handling render this protocol applicable for the synthesis of structurally diverse heterocyclic compounds.

## Experimental section

### General procedure for the three-component reaction of substituted pyridine, DMAD, and 3-phenacylideneoxindoles

A mixture of substituted pyridine (1.2 mmol), DMAD (1.2 mmol, 0.170 g), and 3-phenacylideneoxindole (1.0 mmol) in 10.0 mL of tetrahydrofuran was stirred at room temperature for 6 h. Then, the solvent was removed by evaporation and the residue was subjected to thin-layer chromatography (15 $\times$ 25 cm SiO$_{2}$ plate) with a mixture of light petroleum and ethyl acetate (V/V = 2:1) as the developing reagent. The product was separated from silica gel by eluting with ethanol and is pure enough for spectroscopic analysis.

#### Dimethyl 1-benzyl-6$^\prime$-methyl-2$^\prime$-(4-methylbenzoyl)-2-oxo-2$^\prime$,9a$^\prime$-dihydrospiro[indoline-3,1$^\prime$-quinolizine]-3 $^\prime$,4$^\prime$-dicarboxylate (***1a***)

Yellow solid, 66 %, m.p. 173–175 $$^{\circ }$$C; $$^{1}$$H NMR (600 MHz, DMSO-$$d_{6}) \quad \delta $$: 7.26 (br s, 2H, ArH), 7.20 (br s, 2H, ArH), 7.15–7.13 (m, 4H, ArH), 7.06 (br s, 2H, ArH), 6.89 (br s, 3H, ArH), 6.58 (br s, 1H, CH), 5.60 (brs, 1H, CH), 5.27 (s, 1H, CH), 4.98 (br s, 1H, CH), 4.61 (br s, 2H, CH), 4.50 (br s, 1H, CH), 3.87 (s, 3H, OCH$$_{3})$$, 3.56 (s, 3H, OCH$$_{3})$$, 2.32 (s, 3H, CH$$_{3})$$, 1.93 (s, 3H, CH$$_{3});\,^{13}$$C NMR (150 MHz, CDCl$$_{3}) \quad \delta $$: 196.5, 174.2, 166.3, 166.1, 146.7, 143.4, 136.8, 135.1, 134.9, 128.9, 128.6, 128.4, 128.2, 127.4, 127.1, 126.9, 125.4, 124.9, 122.1, 116.1, 113.9, 108.5, 103.1, 66.8, 58.4, 53.3, 52.2, 49.2, 43.9, 21.7, 20.6; IR (KBr) $$\upsilon $$: 3447, 2946, 2025, 1732, 1710, 1685, 1655, 1611, 1578, 1489, 1465, 1437, 1405, 1368, 1291, 1232, 1177, 1129, 1080, 1017, 963, 815, 793, 738 cm$$^{-1}$$; MS ($$m$$/$$z)$$: HRMS (ESI) Calcd. for C$$_{36}$$H$$_{33}$$N$$_{2}$$O$$_{6}$$ ([M+H]$$^{+})$$: 589.2347. Found: 589.2350.

#### Diethyl 1-butyl-5,6$^\prime$-dimethyl-2$^\prime$-(4-methylbenzoyl)-2-oxo- 2$^\prime$,9a$^\prime$-dihydrospiro[indoline-3,1$^\prime$-quinolizine]-3$^\prime$,4$^\prime$- dicarboxylate (***1b***)

Yellow solid, 53 %, m.p. 161–163 $$^{\circ }$$C; $$^{1}$$H NMR (600 MHz, DMSO-$$d_{6}) \quad \delta $$: 7.39 (d, $$J$$ = 7.8 Hz, 2H, ArH), 7.18 (d, $$J$$ = 7.2 Hz, 2H, ArH), 7.03–7.01 (m, 2H, ArH), 6.73 (d, $$J$$ = 7.8 Hz, 1H, ArH), 6.21 (d, $$J$$ = 7.8 Hz, 1H, CH), 5.50–5.47 (m, 1H, CH), 5.23 (s, 1H, CH), 4.93–4.91 (m, 1H, CH), 4.65 (d, $$J$$ = 9.6 Hz, 1H, CH), 3.95 (s, 3H, OCH$$_{3})$$, 3.39 (s, 3H, OCH$$_{3})$$, 3.24–3.21 (m, 1H, CH), 3.08–3.04 (m, 1H, CH), 2.32 (s, 3H, CH$$_{3})$$, 2.24 (s, 3H, CH$$_{3})$$, 1.67 (s, 3H, CH$$_{3})$$, 0.95 (br s, 3H, CH), 0.69 (t, $$J$$ = 7.2 Hz, 3H, CH$$_{3});\,^{13}$$C NMR (150 MHz, DMSO-$$d_{6}) \quad \delta $$: 196.9, 172.8, 165.1, 164.3, 144.4, 143.2, 139.4, 130.6, 128.9, 128.6, 128.0, 127.8, 127.7, 126.7, 121.1, 121.0, 107.9, 101.9, 63.7, 53.3, 53.0, 51.3, 44.5, 28.4, 21.1, 21.0, 19.4, 13.5; IR (KBr) $$\upsilon $$: 3452, 2952, 2869, 2025, 1747, 1698, 1659, 1615, 1582, 1496, 1436, 1362, 1269, 1237, 1151, 1115, 1083, 1048, 936, 866, 827, 779, 740, 701 cm$$^{-1}$$; MS ($$m$$/$$z)$$: HRMS (ESI) Calcd. for C$$_{34}$$H$$_{37}$$N$$_{2}$$O$$_{6}$$ ([M+H]$$^{+})$$: 569.2686. Found: 569.2671.

#### Dimethyl 1-benzyl-2$^\prime$-(4-methoxybenzoyl)-7$^\prime$-methyl-2-oxo- 2$^\prime$,9a$^\prime$-dihydrospiro[indoline-3,1$^\prime$-quinolizine]-3$^\prime$,4$^\prime$- dicarboxylate (***1c***)

Yellow solid, 61 %, m.p. 190–192 $$^{\circ }$$C; $$^{1}$$H NMR (600 MHz, DMSO-$$d_{6}) \quad \delta $$: 7.51 (d, $$J$$ = 7.8 Hz, 2H, ArH), 7.18 (t, $$J$$ = 7.2 Hz, 1H, ArH), 7.11 (t, $$J$$ = 7.8 Hz, 4H, ArH), 6.98–6.93 (m, 3H, ArH), 6.81 (d, $$J$$ = 7.2 Hz, 2H, ArH), 6.69 (d, $$J$$ = 7.8 Hz, 1H, ArH), 6.05 (s, 1H, CH), 5.54 (d, $$J$$ = 8.4 Hz, 1H, CH), 5.35 (s, 1H, CH), 4.91–4.90 (m, 2H, CH), 4.64 (d, $$J$$ = 15.7 Hz, 1H, CH), 4.49 (d, $$J$$ = 15.7 Hz, 1H, CH), 3.95 (s, 3H, OCH$$_{3})$$, 3.82 (s, 3H, OCH$$_{3})$$, 3.43 (s, 3H, OCH$$_{3})$$, 1.43 (s, 3H, CH$$_{3});\,^{13}$$C NMR (150 MHz, CDCl$$_{3}) \quad \delta $$: 195.8, 174.8, 166.1, 165.3, 163.3, 145.6, 142.6, 135.2, 130.5, 130.4, 128.6, 128.5, 128.2, 127.6, 127.5, 126.9, 125.9, 123.0, 122.5, 116.0, 113.4, 109.5, 108.6, 104.6, 62.7, 55.4, 54.1, 53.4, 51.6, 47.4, 43.9, 17.5; IR (KBr) $$\upsilon $$: 3450, 2949, 2843, 2026, 1742, 1708, 1674, 1609, 1582, 1510, 1489, 1464, 1434, 1412, 1380, 1308, 1244, 1172, 1125, 1021, 983, 941, 899, 825, 776, 746 cm$$^{-1}$$; MS ($$m$$/$$z)$$: HRMS (ESI) Calcd. for C$$_{36}$$H$$_{33}$$N$$_{2}$$O$$_{7}$$ ([M+H]$$^{+})$$: 605.2282. Found: 605.2290.

#### Dimethyl 1-benzyl-5-fluoro-2$^\prime$-(3-methoxybenzoyl)-7$^\prime$-methyl-2-oxo-2$^\prime$,9a$^\prime$-dihydrospiro[indo line-3,1$^\prime$-quinolizine]-3$^\prime$,4$^\prime$-dicarboxylate (***1d***)

Yellow solid, 58 %, m.p. 172–173 $$^{\circ }$$C; $$^{1}$$H NMR (600 MHz, DMSO-$$d_{6}) \quad \delta $$: 7.36 (brs, 1H, ArH), 7.18–7.13 (m, 5H, ArH), 7.05–7.00 (m, 2H, ArH), 6.85 (br s, 3H, ArH), 6.74 (br s, 1H, ArH), 6.11 (s, 1H, CH), 5.59 (br s, 1H,CH), 5.41 (s, 1H, CH), 4.97–4.93 (m, 2H, CH), 4.60 (br s, 1H,CH), 4.48 (br s, 1H,CH), 3.97 (s, 3H, OCH$$_{3})$$, 3.73 (s, 3H, OCH$$_{3})$$, 3.45 (s, 3H, OCH$$_{3})$$, 1.47 (s, 3H, CH$$_{3});\,^{13}$$C NMR (150 MHz, CDCl$$_{3}) \quad \delta $$: 197.1, 174.4, 165.5 (d, $$J$$ = 134.6 Hz), 159.5, 145.7, 138.8, 138.7, 134.9, 129.3, 128.8, 128.5, 127.7, 126.9, 122.5, 120.8, 120.3, 115.7, 115.5 (d, $$J$$ = 24.9 Hz), 115.1 (d, $$J$$ = 24.3 Hz), 111.5, 109.7, 109.1 (d, $$J$$ = 5.7 Hz), 104.3, 62.6, 55.4, 54.4, 53.4, 51.7, 47.9, 44.1, 17.5; IR (KBr) $$\upsilon $$: 3451, 2948, 2839, 2025, 1742, 1710, 1612, 1582, 1486, 1452, 1433, 1410, 1342, 1294, 1251, 1194, 1176, 1117, 1050, 1009, 983, 946, 896, 868, 841, 813, 788, 752 cm$$^{-1}$$; MS ($$m$$/$$z)$$: HRMS (ESI) Calcd. for C$$_{36}$$H$$_{32}$$FN$$_{2}$$O$$_{7}$$ ([M+H]$$^{+})$$: 623.2201. Found: 623.2196.

#### Dimethyl 1-benzyl-5-chloro-7$^\prime$-methyl-2$^\prime$-(4- methylbenzoyl)-2-oxo-2$^\prime$,9a$^\prime$-dihydrospiro[indo-line-3,1$^\prime$- quinolizine]-3$^\prime$,4$^\prime$-dicarboxylate (***1e***)

Yellow solid, 62 %, m.p. 169–172 $$^{\circ }$$C; $$^{1}$$H NMR (600 MHz, DMSO-$$d_{6}) \quad \delta $$: 7.48 (d, $$J$$ = 7.8 Hz, 2H, ArH), 7.24 (d, $$J$$ = 7.8 Hz, 3H, ArH), 7.19 (t, $$J$$ = 7.2 Hz, 1H, ArH), 7.12–7.09 (m, 3H, ArH), 6.76 (d, $$J$$ = 7.2 Hz, 2H, ArH), 6.72 (d, $$J$$ = 8.4 Hz, 1H, ArH), 6.11 (s, 1H, CH), 5.59 (d, $$J$$ = 9.6 Hz, 1H, CH), 5.42 (s, 1H, CH), 4.96 (s, 1H, CH), 4.91 (d, $$J$$ = 9.6 Hz, 1H, CH), 4.64 (d, $$J$$ = 15.6 Hz, 1H, CH), 4.45 (d, $$J$$ = 15.6 Hz, 1H, CH), 3.97 (s, 3H, OCH$$_{3})$$, 3.43 (s, 3H, OCH$$_{3})$$, 2.36 (s, 3H, CH$$_{3})$$, 1.47 (s, 3H, CH$$_{3});\,^{13}$$C NMR (150 MHz, CDCl$$_{3}) \quad \delta $$: 196.7, 174.2, 165.9, 165.1, 145.6, 143.7, 141.2, 134.8, 134.7, 129.0, 128.7, 128.6, 128.4, 127.8, 127.6, 126.9, 122.6, 115.5, 109.8, 109.6, 104.6, 62.6, 54.3, 53.5, 51.7, 47.7, 43.9, 21.7, 17.5; IR (KBr) $$\upsilon $$: 3446, 3040, 2948, 2853, 2025, 1716, 1680, 1611, 1584, 1482, 1434, 1404, 1371, 1295, 1243, 1178, 1115, 1076, 983, 942, 899, 801, 743, 703 cm$$^{-1}$$; MS ($$m$$/$$z)$$: HRMS (ESI) Calcd. for C$$_{36}$$H$$_{32}$$ClN$$_{2}$$O$$_{6}$$ ([M+H]$$^{+})$$: 623.1957. Found: 623.1958.

#### Dimethyl 1-butyl-5-fluoro-2$^\prime$-(4-methoxybenzoyl)-7$^\prime$- methyl-2-oxo-2$^\prime$,9a$^\prime$-dihydrospiro[indo-line-3,1$^\prime$- quinolizine]-3$^\prime$,4$^\prime$-dicarboxylate (***1f***)

Yellow solid, 74 %, m.p. 162–163 $$^{\circ }$$C; $$^{1}$$H NMR (600 MHz, DMSO-$$d_{6}) \quad \delta $$: 7.44 (d, $$J$$ = 9.0 Hz, 2H, ArH), 7.09 (td, $$J_{1}$$ = 9.0 Hz, $$J_{2}$$ = 2.4 Hz, 1H, ArH), 6.90–6.89 (m, 3H, ArH), 6.82 (dd, $$J_{1}$$ = 8.7 Hz, $$J_{2}$$ = 2.4 Hz, 1H, ArH), 6.09 (s, 1H, CH), 5.62 (d, $$J$$ = 9.6 Hz, 1H, CH), 5.28 (s, 1H, CH), 4.92–4.90 (m, 1H, CH), 4.88 (brs, 1H, CH), 3.96 (s, 3H, OCH$$_{3})$$, 3.79 (s, 3H, OCH$$_{3})$$, 3.42 (s, 3H, OCH$$_{3})$$, 3.40–3.36 (m, 1H, CH), 3.25–3.21 (m, 1H, CH), 1.48 (s, 3H, CH$$_{3})$$, 0.99–0.91 (m, 3H, CH), 0.79–0.73 (m, 1H, CH), 0.69 (t, $$J$$ = 7.2 Hz, 3H, CH$$_{3});\,^{13}$$C NMR (150 MHz, DMSO-$$d_{6}) \quad \delta $$: 195.8, 174.0, 165.9, 165.1, 163.3, 158.9 (d, $$J$$ = 119.5 Hz), 145.5, 138.9, 130.5, 130.4, 128.2, 127.9, 127.8, 122.5, 115.8, 115.6 (d, $$J$$ = 25.2 Hz), 114.9 (d, $$J$$ = 23.9 Hz), 113.3, 109.6, 108.1 (d, $$J$$ = 8.3 Hz), 104.3, 62.2, 55.3, 54.1, 53.3, 51.6, 47.4, 40.0, 29.1, 20.0, 17.5, 13.6; IR (KBr) $$\upsilon $$: 3453, 2955, 2924, 2867, 2025, 1746, 1708, 1680, 1600, 1582, 1490, 1455, 1403, 1368, 1329, 1305, 1262, 1237, 1172, 1107, 1076, 1027, 1000, 981, 952, 895, 849, 815, 758 cm$$^{-1}$$; MS ($$m$$/$$z)$$: HRMS (ESI) Calcd. for C$$_{33}$$H$$_{34}$$FN$$_{2}$$O$$_{7}$$ ([M+H]$$^{+})$$: 589.2358. Found: 589.2353.

#### Dimethyl 1-butyl-5-fluoro-2$^\prime$-(4-methoxybenzoyl)-8$^\prime$- methyl-2-oxo-2$^\prime$,9a$^\prime$-dihydrospiro[indo-line-3,1$^\prime$- quinolizine]-3$^\prime$,4$^\prime$-dicarboxylate (***1g***)

Yellow solid, 77 %, m.p. 164–166 $$^{\circ }$$C; $$^{1}$$H NMR (600 MHz, DMSO-$$d_{6}) \quad \delta $$: 7.42 (d, $$J$$ = 7.2 Hz, 2H, ArH), 7.08 (br s, 1H, ArH), 6.89 (d, $$J$$ = 7.2 Hz, 3H, ArH), 6.79 (d, $$J$$ = 7.8 Hz, 1H, ArH), 6.33 (d, $$J$$ = 7.2 Hz, 1H, CH), 5.26 (s, 1H, CH), 4.88 (s, 1H, CH), 4.73 (d, $$J$$ = 7.2 Hz, 1H, CH), 4.61 (s, 1H, CH), 3.95 (s, 3H, OCH$$_{3})$$, 3.79 (s, 3H, OCH$$_{3})$$, 3.44 (s, 3H, OCH$$_{3})$$, 3.36 (br s, 1H, CH), 3.29 (br s, 1H, CH), 1.38 (s, 3H, CH$$_{3})$$, 1.06 (brs, 1H, CH), 0.98–0.97 (m, 2H, CH), 0.86 (brs, 1H, CH), 0.71 (br s, 3H, CH$$_{3});\,^{13}$$C NMR (150 MHz, CDCl$$_{3})$$ $$\delta $$: 195.9, 174.1, 165.8, 164.9, 163.3, 145.4, 139.0, 132.6, 130.5, 130.4, 128.0, 127.9, 126.6, 115.5 (d, $$J$$ = 25.2 Hz), 114.9 (d, $$J$$ = 24.3 Hz), 113.6, 113.5, 113.3, 112.1, 110.2, 108.0 (d, $$J$$ = 8.3 Hz), 105.8, 104.7, 63.1, 56.9, 55.4, 54.4, 53.4, 51.7, 47.5, 40.0, 29.4, 29.1, 20.8, 20.5, 20.1, 20.0, 13.7; IR (KBr) $$\upsilon $$: 3449, 2953, 2927, 2867, 2026, 1734, 1700, 1671, 1595, 1490, 1437, 1377, 1313, 1278, 1245, 1200, 1174, 1141, 1120, 1023, 952, 885, 853, 816, 790, 755, 729 cm$$^{-1}$$; MS ($$m$$/$$z)$$: HRMS (ESI) Calcd. for C$$_{33}$$H$$_{34}$$FN$$_{2}$$O$$_{7}$$ ([M+H]$$^{+})$$: 589.2358. Found: 589.2366.

#### Dimethyl 1-butyl-5-chloro-2$^\prime$-(4-methoxybenzoyl)-8$^\prime$- methyl-2-oxo-2$^\prime$,9a$^\prime$-dihydrospiro[indo-line-3,1$^\prime$- quinolizine]-3$^\prime$,4$^\prime$-dicarboxylate (***1h***)

Yellow solid, 75 %, m.p. 168–171 $$^{\circ }$$C; $$^{1}$$H NMR (600 MHz, DMSO-$$d_{6}) \quad \delta $$: 7.43 (d, $$J$$ = 7.8 Hz, 2H, ArH), 7.29 (d, $$J$$ = 7.8 Hz, 1H, ArH), 7.02 (s, 1H, ArH), 6.90 (t, $$J$$ = 8.4 Hz, 3H, ArH), 6.33 (d, $$J$$ = 7.8 Hz, 1H, CH), 5.27 (s, 1H, CH), 4.89 (s, 1H, CH), 4.73 (d, $$J$$ = 7.2 Hz, 1H, CH), 4.60 (s, 1H, CH), 3.95 (s, 3H, OCH$$_{3})$$, 3.79 (s, 3H, OCH$$_{3})$$, 3.44 (s, 3H, OCH$$_{3})$$, 3.37 (brs, 1H, CH), 3.29–3.26 (m, 1H, CH), 1.38 (s, 3H, CH$$_{3})$$, 1.05 (br s, 1H, CH), 0.99–0.95 (m, 2H, CH), 0.86 (br s, 1H, CH), 0.71 (t, $$J$$ = 7.2 Hz, 3H, CH$$_{3});\,^{13}$$C NMR (150 MHz, CDCl$$_{3})$$ $$\delta $$: 195.7, 173.9, 165.8, 164.9, 163.3, 145.4, 141.6, 132.7, 130.4, 128.4, 127.9, 127.8, 127.7, 126.5, 113.3, 110.1, 108.5, 106.0, 104.7, 63.2, 55.4, 54.4, 53.4, 51.7, 47.5, 39.9, 29.1, 20.8, 20.0, 13.7; IR (KBr) $$\upsilon $$: 3456, 3071, 2952, 2869, 2587, 2027, 1742, 1710, 1672, 1605, 1579, 1511, 1483, 1432, 1381, 1324, 1243, 1173, 1127, 1045, 1023, 982, 955, 913, 869, 836, 810, 730 cm$$^{-1}$$; MS ($$m$$/$$z)$$: HRMS (ESI) Calcd. for C$$_{33}$$H$$_{34}$$ClN$$_{2}$$O$$_{7}$$ ([M+H]$$^{+})$$: 605.2062. Found: 605.2067.

#### Dimethyl 1-benzyl-5-chloro-8$^\prime$-methoxy-2$^\prime$-(4- methoxybenzoyl)-2-oxo-2$^\prime$,9a$^\prime$-dihydrospiro-[indoline-3,1$^\prime$- quinolizine]-3$^\prime$,4$^\prime$-dicarboxylate (***1i***)

Yellow solid, 89 %, m.p. 150.0–150.3 $$^{\circ }$$C; $$^{1}$$H NMR (600 MHz, DMSO-$$d_{6}) \quad \delta $$: 7.54 (d, $$J$$ = 7.2 Hz, 2H, ArH), 7.25 (d, $$J$$ = 6.6 Hz, 1H, ArH), 7.18 (d, $$J$$ = 6.6 Hz, 1H, ArH), 7.13 (br s, 2H, ArH), 7.01 (s, 1H, ArH), 6.97–6.95 (m, 4H, ArH), 6.82 (d, $$J$$ = 7.8 Hz, 1H, ArH), 6.43 (d, $$J$$ = 7.8 Hz, 1H, CH), 5.36 (s, 1H, CH), 5.05 (s, 1H, CH), 4.66 (d, $$J$$ = 7.2 Hz, 1H, CH), 4.60–4.54 (m, 2H, CH$$_{2})$$, 3.95 (s, 3H, OCH$$_{3})$$, 3.82 (s, 3H, OCH$$_{3})$$, 3.77 (brs, 1H, CH), 3.47 (s, 3H, OCH$$_{3})$$, 2.93 (s, 3H, OCH$$_{3});\,^{13}$$C NMR (150 MHz, CDCl$$_{3}) \quad \delta $$: 195.5, 174.5, 165.7, 164.8, 163.5, 153.7, 145.2, 141.4, 135.0, 130.7, 130.6, 130.2, 128.8, 128.6, 128.4, 127.8, 127.7, 127.5, 127.1, 113.5, 113.4, 109.1, 100.2, 83.0, 64.0, 58.4, 55.5, 55.3, 54.1, 53.5, 53.4, 51.9, 47.3, 43.9, 18.4, 15.3; IR (KBr) $$\upsilon $$: 3457, 2954, 1745, 1707, 1671, 1628, 1600, 1511, 1484, 1455, 1435, 1377, 1339, 1251, 1226, 1178, 1136, 979, 944, 902, 868, 845, 808, 757 cm$$^{-1}$$; MS ($$m$$/$$z)$$: HRMS (ESI) Calcd. for C$$_{36}$$H$$_{32}$$Cl$$_{2}$$N$$_{2}$$O$$_{8}$$ ([M+H]$$^{+})$$: 655.1842. Found: 655.1841.

#### Dimethyl 1-benzyl-5-chloro-8$^\prime$-methoxy-2$^\prime$-(4- methylbenzoyl)-2-oxo-2$^\prime$,9a$^\prime$-dihydrospiro-[indoline-3,1$^\prime$- quinolizine]-3$^\prime$,4$^\prime$-dicarboxylate (***1j***)

Yellow solid, 84 %, m.p. 162.3–163.1 $$^{\circ }$$C; $$^{1}$$H NMR (600 MHz, DMSO-$$d_{6}) \quad \delta $$: 7.42 (d, $$J$$ = 7.8 Hz, 2H, ArH), 7.27–7.24 (m, 3H, ArH), 7.18 (d, $$J$$ = 7.2 Hz, 1H, ArH), 7.14 (t, $$J$$ = 7.2 Hz, 2H, ArH), 7.00 (s, 1H, ArH), 6.96 (d, $$J$$ = 7.8 Hz, 2H, ArH), 6.82 (d, $$J$$ = 8.4 Hz, 1H, ArH), 6.43 (d, $$J$$ = 8.4 Hz, 1H, CH), 5.37 (s, 1H, CH), 5.06 (s, 1H, CH), 4.66 (d, $$J$$ = 7.2 Hz, 1H, CH), 4.49 (br s, 2H, CH$$_{2})$$, 3.95 (s, 3H, OCH$$_{3})$$, 3.75 (brs, 1H, CH), 3.48 (s, 3H, OCH$$_{3})$$, 2.91 (s, 3H, OCH$$_{3})$$, 2.36 (s, 3H, CH$$_{3});\,^{13}$$C NMR (150 MHz, CDCl$$_{3}) \quad \delta $$: 196.8, 174.4, 173.4, 165.7, 164.8, 153.7, 145.2, 143.7, 141.4, 135.1, 135.0, 134.8, 129.2, 128.9, 128.8, 128.7, 128.5, 128.3, 127.9, 127.8, 127.7, 127.5, 127.3, 126.9, 100.1, 83.0, 63.9, 55.1, 54.0, 53.5, 51.9, 47.8, 43.9, 21.7, 21.6; IR (KBr) $$\upsilon $$: 3455, 2945, 1744, 1716, 1672, 1626, 1600, 1480, 1456, 1432, 1388, 1370, 1317, 1246, 1231, 1175, 1135, 1043, 977, 947, 918, 869, 838, 814, 784 cm$$^{-1}$$; MS ($$m$$/$$z)$$: HRMS (ESI) Calcd. for C$$_{36}$$H$$_{32}$$ClN$$_{2}$$O$$_{7}$$ ([M+H]$$^{+})$$: 639.1893. Found: 639.1898.

#### Dimethyl 1-benzyl-5-fluoro-8$^\prime$-methoxy-2$^\prime$-(4- methoxybenzoyl)-2-oxo-2$^\prime$,9a$^\prime$-dihydrospiro-[indoline-3,1$^\prime$- quinolizine]-3$^\prime$,4$^\prime$-dicarboxylate (***1k***)

Yellow solid, 91 %, m.p. 147.1–148.0 $$^{\circ }$$C; $$^{1}$$H NMR (600 MHz, DMSO-$$d_{6}) \quad \delta $$: 7.54 (d, $$J$$ = 8.4 Hz, 2H, ArH), 7.18 (t, $$J$$ = 7.2 Hz, 1H, ArH), 7.13 (d, $$J$$ = 7.2 Hz, 2H, ArH), 7.04 (t, $$J$$ = 8.4 Hz, 1H, ArH), 6.96 (t, $$J$$ = 7.8 Hz, 4H, ArH), 6.79–6.78 (m, 2H, ArH), 6.43 (d, $$J$$ = 7.8 Hz, 1H, CH), 5.35 (s, 1H, CH), 5.04 (d, $$J$$ = 3.0 Hz, 1H, CH), 4.65 (d, $$J$$ = 6.6 Hz, 1H, CH), 4.56 (brs, 2H, CH$$_{2})$$, 3.95 (s, 3H, OCH$$_{3})$$, 3.83 (s, 3H, OCH$$_{3})$$, 3.78 (br s, 1H, CH), 3.47 (s, 3H, OCH$$_{3})$$, 2.93 (s, 3H, OCH$$_{3});\,^{13}$$C NMR (150 MHz, DMSO-$$d_{6}) \quad \delta $$: 195.3, 173.6, 165.0, 164.0, 163.3, 157.9 (d, $$J$$ = 236.9 Hz), 153.0, 144.8, 139.1, 135.6, 130.2, 129.6, 129.3, 128.5, 127.4, 127.3, 127.2, 115.1 (d, $$J$$ = 23.1 Hz), 114.3 (d, $$J$$ = 28.2 Hz), 113.6, 109.4 (d, $$J$$ = 6.2 Hz), 106.1, 99.7, 82.8, 63.3, 56.0, 55.5, 54.5, 53.8, 53.4, 51.6, 46.4, 43.0, 18.5; IR (KBr) $$\upsilon $$: 3450, 1737, 1641, 1488, 1422, 1369, 1285, 1232, 1187, 1111, 952, 865, 816, 774 cm$$^{-1}$$; MS ($$m$$/$$z)$$: HRMS (ESI) Calcd. for C$$_{36}$$H$$_{32}$$FN$$_{2}$$O$$_{8}$$ ([M+H]$$^{+})$$: 639.2137. Found: 639.2142.

#### Dimethyl 1-benzyl-5-fluoro-8$^\prime$-methoxy-2$^\prime$-(4-methylbenzoyl)-2-oxo-2$^\prime$,9a$^\prime$-dihydrospiro[indo line-3,1$^\prime$-quinolizine]-3$^\prime$,4$^\prime$-dicarboxylate (***1l***)

Yellow solid, 87 %, m.p. 158.5–159.0 $$^{\circ }$$C; $$^{1}$$H NMR (600 MHz, DMSO-$$d_{6}) \quad \delta $$: 7.42 (d, $$J$$ = 7.8 Hz, 2H, ArH), 7.24 (t, $$J$$ = 7.8 Hz, 2H, ArH), 7.18 (t, $$J$$ = 7.2 Hz, 1H, ArH), 7.14 (t, $$J$$ = 7.2 Hz, 2H, ArH), 7.06 (t, $$J$$ = 8.4 Hz, 1H, ArH), 6.97 (t, $$J$$ = 7.2 Hz, 2H, ArH), 6.81–6.77 (m, 2H, ArH), 6.43 (d, $$J$$ = 8.4 Hz, 1H, CH), 5.37 (s, 1H, CH), 5.05 (d, $$J$$ = 8.4 Hz, 1H, CH), 4.66 (d, $$J$$ = 7.8 Hz, 1H, CH), 4.48 (brs, 2H, CH$$_{2})$$, 3.95 (s, 3H, OCH$$_{3})$$, 3.76 (brs, 1H, CH), 3.48 (s, 3H, OCH$$_{3})$$, 2.91 (s, 3H, OCH$$_{3})$$, 2.36 (s, 3H, CH$$_{3});\,^{13}$$C NMR (150 MHz, CDCl$$_{3}) \quad \delta $$: 196.9, 174.6, 165.7, 164.8, 159.0 (d, $$J$$ = 240 Hz), 153.6, 145.2, 143.7, 138.8, 135.2 134.8, 128.9, 128.8, 128.3, 127.7, 115.4 (d, $$J$$ = 25.4 Hz), 115.1 (d, $$J$$ = 23.4 Hz), 108.7 (d, $$J$$ = 7.5 Hz), 106.5, 100.2, 83.1, 63.9, 55.2, 54.1, 53.5, 51.8, 47.7, 43.9, 21.6; IR (KBr) $$\upsilon $$: 3454, 2946, 1745, 1715, 1675, 1625, 1597, 1487, 1454, 1434, 1387, 1317, 1297, 1243, 1227, 1179, 1153, 1128, 1045, 981, 947, 892, 868, 843, 809, 767 cm$$^{-1}$$; MS ($$m$$/$$z)$$: HRMS (ESI) Calcd. for C$$_{36}$$H$$_{32}$$FN$$_{2}$$O$$_{7}$$ ([M+H]$$^{+})$$: 623.2188. Found: 623.2190.

#### Dimethyl 1-benzyl-5-fluoro-8$^\prime$-methoxy-2$^\prime$-benzoyl-2-oxo- 2$^\prime$,9a$^\prime$-dihydrospiro[indoline-3,1$^\prime$-quinolizine]-3$^\prime$,4$^\prime$- dicarboxylate (***1m***)

Yellow solid, 81 %, m.p. 137.2–137.6 $$^{\circ }$$C; $$^{1}$$H NMR (600 MHz, DMSO-$$d_{6}) \quad \delta $$: 7.63 (brs, 1H, ArH), 7.46–7.44 (m, 4H, ArH), 7.17 (brs, 3H, ArH), 7.08 (brs, 1H, ArH), 7.02 (brs, 2H, ArH), 6.83–6.77 (m, 2H, ArH), 6.44 (d, $$J$$ = 8.4 Hz, 1H, CH), 5.39 (s, 1H, CH), 5.06 (brs, 1H, CH), 4.66 (d, $$J$$ = 5.4 Hz, 1H, CH), 4.46 (d, $$J$$ = 15.0 Hz, 1H, CH), 4.36 (d, $$J$$ = 15.0 Hz, 1H, CH), 3.96 (s, 3H, OCH$$_{3})$$, 3.75 (br s, 1H, CH), 3.50 (s, 3H, OCH$$_{3})$$, 2.88 (s, 3H, OCH$$_{3});\,^{13}$$C NMR (150 MHz, CDCl$$_{3}) \quad \delta $$: 197.5, 174.5, 165.7, 164.7, 159.0 (d, $$J$$ = 239.9 Hz), 153.6, 145.2, 138.9, 137.5, 135.3, 132.9, 128.8, 128.7, 128.2, 128.1, 127.8, 127.6, 115.4 (d, $$J$$ = 21.2 Hz), 115.2 (d, $$J$$ = 19.7 Hz), 108.7 (d, $$J$$ = 8.4 Hz), 106.3, 100.3, 83.1, 63.8, 55.1, 54.0, 53.5, 51.9, 48.1, 43.9; IR (KBr) $$\upsilon $$: 3450, 2948, 1752, 1712, 1647, 1631, 1598, 1488, 1436, 1388, 1335, 1296, 1227, 1155, 1131, 1051, 980, 940, 908, 894, 862, 826, 768 cm$$^{-1}$$; MS ($$m$$/$$z)$$: HRMS (ESI) Calcd. for C$$_{35}$$H$$_{30}$$FN$$_{2}$$O$$_{7}$$ ([M+H]$$^{+})$$: 609.2032. Found: 609.2034.

#### Dimethyl 1-butyl-5-chloro-8$^\prime$-methoxy-2$^\prime$-(4- methylbenzoyl)-2-oxo-2$^\prime$,9a$^\prime$-dihydrospiro[indo-line-3,1$^\prime$- quinolizine]-3$^\prime$,4$^\prime$-dicarboxylate (***1n***)

Yellow solid, 93 %, m.p. 162.1–163.0 $$^{\circ }$$C; $$^{1}$$H NMR (600 MHz, DMSO-$$d_{6}) \quad \delta $$: 7.41 (d, $$J$$ = 8.4 Hz, 2H, ArH), 7.30 (dd, $$J_{1}$$ = 8.4 Hz, $$J_{2}$$ = 1.8 Hz, 1H, ArH), 6.98 (d, $$J$$ = 1.8 Hz, 1H, ArH), 6.90 (dd, $$J_{1}$$ = 5.7 Hz, $$J_{2}$$ = 3.0 Hz, 3H, ArH), 6.43 (d, $$J$$ = 7.8 Hz, 1H, CH), 5.26 (s, 1H, CH), 4.98 (d, $$J$$ = 3.0 Hz, 1H, CH), 4.70 (dd, $$J_{1}$$ = 7.8 Hz, $$J_{2}$$ = 1.8 Hz, 1H, CH), 3.95 (s, 3H, OCH$$_{3})$$, 3.85 (br s, 1H, CH), 3.79 (s, 3H, OCH$$_{3})$$, 3.47 (s, 3H, OCH$$_{3})$$, 3.38–3.33 (m, 1H, CH), 3.30–3.27 (m, 1H, CH), 3.18 (s, 3H, OCH$$_{3})$$, 1.10–1.05 (m, 1H, CH), 1.00–0.96 (m, 2H, CH), 0.94–0.89 (m, 1H, CH), 0.71 (t, $$J$$ = 7.2 Hz, 3H, CH$$_{3});\,^{13}$$C NMR (150 MHz, CDCl$$_{3}) \quad \delta $$: 195.7, 174.1, 165.7, 164.8, 163.3, 163.2, 153.6, 145.2, 141.8, 130.4, 128.8, 128.6, 128.0, 127.7, 125.8, 113.6, 113.3, 108.4, 106.8, 102.5, 100.1, 83.1, 63.7, 57.6, 55.4, 55.2, 54.1, 53.4, 51.8, 47.5, 39.9, 29.3, 20.1, 13.6; IR (KBr) $$\upsilon $$: 3450, 2953, 1737, 1713, 1670, 1626, 1601, 1574, 1510, 1483, 1459, 1432, 1384, 1323, 1246, 1175, 1133, 1116, 1027, 979, 940, 915, 873, 848, 812, 780 cm$$^{-1}$$; MS ($$m$$/$$z)$$: HRMS (ESI) Calcd. for C$$_{33}$$H$$_{34}$$ClN$$_{2}$$O$$_{8}$$ ([M+H]$$^{+})$$: 621.1998. Found: 621.1997.

#### Dimethyl 1-butyl-5-fluoro-8$^\prime$-methoxy-2$^\prime$-(4- methylbenzoyl)-2-oxo-2$^\prime$,9a$^\prime$-dihydrospiro[indo-line-3,1$^\prime$- quinolizine]-3$^\prime$,4$^\prime$-dicarboxylate (***1o***)

Yellow solid, 90 %, m.p. 176.7–177.2 $$^{\circ }$$C; $$^{1}$$H NMR (600 MHz, DMSO-$$d_{6}) \quad \delta $$: 7.28 (d, $$J$$ = 8.4 Hz, 2H, ArH), 7.17 (d, $$J$$ = 7.8 Hz, 2H, ArH), 7.10 (td, $$J_{1}$$ = 9.0 Hz, $$J_{2}$$ = 2.4 Hz, 1H, ArH), 6.87 (dd, $$J_{1}$$ = 8.4 Hz, $$J_{2}$$ = 4.2 Hz, 1H, ArH), 6.75 (dd, $$J_{1}$$ = 8.4 Hz, $$J_{2}$$ = 2.4 Hz, 1H, ArH), 6.43 (d, $$J$$ = 7.8 Hz, 1H, CH), 5.27 (s, 1H, CH), 4.97 (d, $$J$$ = 3.6 Hz, 1H, CH), 4.70 (dd, $$J_{1}$$ = 8.4 Hz, $$J_{2}$$ = 2.4 Hz, 1H, CH), 3.95 (s, 3H, OCH$$_{3})$$, 3.86 (br s, 1H, CH), 3.48 (s, 3H, OCH$$_{3})$$, 3.30–3.20 (m, 2H, CH), 3.18 (s, 3H, OCH$$_{3})$$, 2.31 (s, 3H, CH$$_{3})$$, 1.09–1.03 (m, 1H, CH), 1.02–0.97 (m, 2H, CH), 0.92–0.85 (m, 1H, CH), 0.72 (t, $$J$$ = 7.2 Hz, 3H, CH$$_{3});\,^{13}$$C NMR (150 MHz, CDCl$$_{3}) \quad \delta $$: 197.2, 174.1, 165.7, 164.8, 158.9 (d, $$J$$ = 239.4 Hz), 153.5, 145.2, 143.5, 139.2, 135.0, 129.1, 128.8, 128.3, 128.1, 115.4 (d, $$J$$ = 25.1 Hz), 107.9 (d, $$J$$ = 8.3 Hz), 106.6, 102.6, 100.2, 83.2, 63.6, 57.6, 55.1, 54.1, 53.4, 51.8, 47.8, 39.9, 29.2, 21.6, 20.1, 13.7; IR (KBr) $$\upsilon $$: 3452, 2951, 1751, 1709, 1676, 1629, 1599, 1491, 1456, 1437, 1378, 1322, 1275, 1229, 1197, 1181, 1159, 1134, 1048, 1005, 975, 940, 901, 868, 839, 823, 761 cm$$^{-1}$$; MS ($$m$$/$$z)$$: HRMS (ESI) Calcd. for C$$_{33}$$H$$_{34}$$FN$$_{2}$$O$$_{7}$$ ([M+H]$$^{+})$$: 589.2345. Found: 589.2345.

#### Dimethyl 1-benzyl-2$^\prime$-(4-methylbenzoyl)-2,8$^\prime$-dioxo- 2$^\prime$,8$^\prime$,9$^\prime$,9a$^\prime$-tetrahydrospiro[indoline-3,1$^\prime$-quinolizine]-3$^\prime$,4$^\prime$- dicarboxylate (***2a***)

White solid, m.p. 188.8–188.9 $$^{\circ }$$C; $$^{1}$$H NMR (600 MHz, DMSO-$$d_{6}) \quad \delta $$: 7.59 (br s, 2H, ArH), 7.36 (br s, 1H, ArH), 7.19–7.13 (m, 8H, ArH), 6.80 (br s, 2H, ArH), 6.56 (brs, 1H, CH), 5.28 (br s, 2H, CH), 4.46 (d, $$J$$ = 13.2 Hz, 1H, CH), 4.39 (d, $$J$$ = 15.6 Hz, 1H, CH), 4.29 (d, $$J$$ = 15.6 Hz, 1H, CH), 4.06 (s, 3H, OCH$$_{3})$$, 3.51 (s, 3H, OCH$$_{3})$$, 2.40 (s, 3H, CH$$_{3})$$, 2.22 (d, $$J$$ = 15.6 Hz, 1H, CH), 1.89 (t, $$J$$ = 15.6 Hz, 1H, CH); $$^{13}$$C NMR (150 MHz, CDCl$$_{3}) \quad \delta $$: 195.4, 190.3, 174.3, 165.3, 164.0, 144.4, 142.7, 134.6, 134.0, 129.6, 129.2, 128.8, 128.6, 127.7, 127.1, 127.0, 124.2, 123.9, 109.6, 107.3, 106.0, 59.0, 53.8, 52.0, 51.8, 45.9, 44.2, 36.1, 21.7; IR (KBr) $$\upsilon $$: 3453, 1752, 1715, 1664, 1637, 1593, 1487, 1466, 1453, 1367, 1326, 1292, 1254, 1218, 1183, 1129, 1106, 945, 904, 795, 754 cm$$^{-1}$$; MS ($$m$$/$$z)$$: HRMS (ESI) Calcd. for C$$_{35}$$H$$_{30}$$N$$_{2}$$NaO$$_{7}$$ ([M+Na]$$^{+})$$: 613.1945. Found: 613.1947.

#### Dimethyl 1-benzyl-5-chloro-2$^\prime$-(4-methylbenzoyl)-2,8$^\prime$-dioxo-2$^\prime$,8$^\prime$,9$^\prime$,9a$^\prime$-tetrahydrospiro[indo line-3,1$^\prime$-quinolizine]-3$^\prime$,4$^\prime$-dicarboxylate (***2b***)

White solid, m.p. 181.1–181.3 $$^{\circ }$$C; $$^{1}$$H NMR (600 MHz, DMSO-$$d_{6}) \quad \delta $$: 7.89 (br s, 2H, ArH), 7.59 (br s, 1H, ArH), 7.47 (br s, 1H, ArH), 7.29 (br s, 5H, ArH), 7.13 (br s, 4H, ArH, CH), 5.34 (s, 1H, CH), 4.91 (brs, 2H, CH), 4.66–4.63 (m, 2H, CH), 3.99 (s, 3H, OCH$$_{3})$$, 3.47 (s, 3H, OCH$$_{3})$$, 2.38 (s, 3H, CH$$_{3})$$, 1.87 (br s, 2H, CH); $$^{13}$$C NMR (150 MHz, CDCl$$_{3}) \quad \delta $$: 197.6, 190.3, 172.4, 164.9, 164.0, 144.7, 144.2, 144.0, 141.0, 135.1, 134.7, 129.8, 129.6, 129.4, 128.9, 128.7, 128.1, 127.6, 125.5, 110.7, 107.7, 102.6, 54.9, 53.8, 52.3, 51.0, 44.2, 43.3, 36.3, 21.7; IR (KBr) $$\upsilon $$: 3452, 2956, 1740, 1713, 1678, 1633, 1594, 1479, 1439, 1383, 1326, 1300, 1249, 1186, 1131, 1081, 1002, 956, 936, 898, 883, 855, 815, 781, 737, 704 cm$$^{-1}$$; MS ($$m$$/$$z)$$: HRMS (ESI) Calcd. for C$$_{35}$$H$$_{30}$$ClN$$_{2}$$O$$_{7}$$ ([M+H]$$^{+})$$: 625.1736. Found: 625.1745.

### General procedure for the three-component reaction of quinoline, DMAD, and 3-methyleneoxindoles

A mixture of quinoline (1.5 mmol), DMAD (1.5 mmol, 0.213 g), and 3-methyleneoxindole (1.0 mmol) in 10.0 mL of dimethoxyethane (DME) was stirred at room temperature for 6 h. Then, the solvent was removed by evaporation and the residue was quickly subjected to thin-layer chromatography (15 $\times$ 25 cm SiO$_{2}$ plate) with a mixture of light petroleum and ethyl acetate (V/V = 2:1) as the developing reagent.

#### Dimethyl 1-benzyl-5-chloro-3$^\prime$-(4-methylbenzoyl)-2-oxo- 3$^\prime$,4a$^\prime$-dihydrospiro[indoline-3,4$^\prime$-pyrido[1,2-a]quinoline]- 1$^\prime$,2$^\prime$-dicarboxylate (***3a***)

Yellow solid, 40 %, m.p. 183–186 $$^{\circ }$$C; $$^{1}$$H NMR (600 MHz, DMSO-$$d_{6}) \quad \delta $$: 7.36 (br s, 2H, ArH), 7.23 (b rs, 2H, ArH), 7.16 (br s, 4H, ArH), 6.98–6.93 (m, 2H, ArH), 6.86 (br s, 4H, ArH), 6.65 (br s, 1H, ArH), 6.57 (br s, 1H, ArH), 6.33 (d, $$J$$ = 5.4 Hz, 1H, CH), 5.42 (s, 1H, CH), 5.33 (brs, 1H, CH), 4.72–4.61 (br s, 3H, CH), 3.88 (s, 3H, OCH$$_{3})$$, 3.61 (s, 3H, OCH$$_{3})$$, 2.33 (s, 3H, CH$$_{3});\,^{13}$$C NMR (150 MHz, CDCl$$_{3}) \quad \delta $$: 195.4, 173.8, 166.0, 165.2, 146.1, 143.7, 141.5, 138.1, 134.7, 134.6, 129.9, 129.2, 129.1, 128.7, 128.5, 128.4, 128.1, 127.8, 127.7, 126.9, 126.7, 122.4, 121.6, 118.9, 118.0, 114.7, 109.2, 65.0, 59.3, 53.1, 52.3, 49.4, 44.1; IR (KBr) $$\upsilon $$: 3448, 2949, 1721, 1701, 1683, 1640, 1571, 1490, 1432, 1382, 1356, 1302, 1242, 1179, 1114, 972, 817 cm$$^{-1}$$; MS ($$m$$/$$z)$$: HRMS (ESI) Calcd. for C$$_{39}$$H$$_{32}$$ClN$$_{2}$$O$$_{6}$$ ([M+H]$$^{+})$$: 659.1943. Found: 659.1941.

#### Dimethyl 1-benzyl-5-chloro-3$^\prime$-(4-chlorobenzoyl)-2-oxo- 3$^\prime$,4a$^\prime$-dihydrospiro[indoline-3,4$^\prime$-pyrido[1,2-a]quinoline]- 1$^\prime$,2$^\prime$-dicarboxylate (***3b***)

Yellow solid, 52 %, m.p. 171–173 $$^{\circ }$$C; $$^{1}$$H NMR (600 MHz, DMSO-$$d_{6}) \quad \delta $$: 7.45 (br s, 4H, ArH), 7.22 (br s, 4H, ArH), 7.02 (br s, 1H, ArH), 6.87 (brs, 5H, ArH), 6.66 (br s, 2H, ArH), 6.35 (br s, 1H, CH), 5.45 (br s, 1H, CH), 5.34 (br s, 1H, CH), 4.76–4.64 (m, 3H, CH), 3.89 (s, 3H, OCH$$_{3})$$, 3.62 (s, 3H, OCH$$_{3});\,^{13}$$C NMR (150 MHz, DMSO-$$d_{6}) \quad \delta $$: 194.8, 172.6, 165.4, 164.4, 150.5, 144.9, 141.7, 138.6, 137.5, 135.9, 135.2, 135.0, 129.8, 129.5, 128.9, 128.5, 128.3, 128.1, 128.0, 127.9, 127.4, 127.0, 126.1, 125.8, 122.4, 121.4, 118.6, 118.3, 113.9, 110.1, 64.3, 58.0, 53.1, 48.7, 43.2; IR (KBr) $$\upsilon $$: 3448, 2949, 1722, 1702, 1641, 1614, 1490, 1433, 1401, 1382, 1356, 1304, 1241, 1190, 1130, 1090, 1015, 968, 917, 850, 817 cm$$^{-1}$$; MS ($$m$$/$$z)$$: HRMS (ESI) Calcd. for C$$_{38}$$H$$_{29}$$Cl$$_{2}$$N$$_{2}$$O$$_{6}$$ ([M+H]$$^{+})$$: 679.1397. Found: 679.1392.

#### Dimethyl 1-benzyl-5-fluoro-3$^\prime$-benzoyl-2-oxo-3$^\prime$,4a$^\prime$- dihydrospiro[indoline-3,4$^\prime$-pyrido[1,2-a]-quinoline]-1$^\prime$,2$^\prime$- dicarboxylate (***3c***)

Yellow solid, 55 %, m.p. 166–167 $$^{\circ }$$C; $$^{1}$$H NMR (600 MHz, DMSO-$$d_{6}) \quad \delta $$: 7.58 (br s, 1H, ArH), 7.41–7.36 (m, 4H, ArH), 7.21 (br s, 4H, ArH), 6.95–6.80 (m, 6H, ArH), 6.56 (br s, 1H, ArH), 6.41–6.40 (m, 1H, ArH), 6.34–6.33 (m, 1H, CH), 5.44 (s, 1H, CH), 5.32 (d, $$J$$ = 6.6 Hz, 1H, CH), 4.68–4.61 (m, 3H, CH), 3.89 (s, 3H, OCH$$_{3})$$, 3.63 (s, 3H, OCH$$_{3});\,^{13}$$C NMR (150 MHz, DMSO-$$d_{6})$$ $$\delta $$: 195.8, 172.9, 165.5, 164.5, 157.2 (d, $$J$$ = 236.4 Hz), 150.5, 145.0, 139.3, 137.5, 136.5, 136.0, 135.3, 133.4, 129.8, 128.6, 127.9, 127.8, 127.6, 127.4, 126.9, 126.2 (d, $$J$$ = 8.7 Hz), 122.3, 121.4, 118.7, 118.5, 115.5 (d, $$J$$ = 25.7 Hz), 114.8 (d, $$J$$ = 23.0 Hz), 113.9, 109.5 (d, $$J$$ = 8.3 Hz), 64.3, 58.4, 53.1, 52.3, 48.9, 43.3; IR (KBr) $$\upsilon $$: 3448, 2952, 1737, 1711, 1633, 1605, 1571, 1491, 1437, 1407, 1383, 1360, 1180, 1114, 1081, 1014, 967, 875, 819, 768 cm$$^{-1}$$; MS ($$m$$/$$z)$$: HRMS (ESI) Calcd. for C$$_{38}$$H$$_{30}$$FN$$_{2}$$O$$_{6}$$ ([M+H]$$^{+})$$: 629.2082. Found: 629.2078.

#### Dimethyl 1-benzyl-5-fluoro-3$^\prime$-(4-chlorobenzoyl)-2-oxo- 3$^\prime$,4a$^\prime$-dihydrospiro[indoline-3,4$^\prime$-pyrido[1,2-a]quinoline]- 1$^\prime$,2$^\prime$-dicarboxylate (***3d***)

Yellow solid, 53 %, m.p. 170–171 $$^{\circ }$$C; $$^{1}$$H NMR (600 MHz, DMSO-$$d_{6}) \quad \delta $$: 7.45 (br s, 4H, ArH), 7.22 (br s, 4H, ArH), 6.90 (br s, 6H, ArH), 6.65 (br s, 1H, ArH), 6.42–6.35 (m, 2H, ArH, CH), 5.44 (s, 1H, CH), 5.35 (brs, 1H, CH), 4.76–4.65 (m, 3H, CH), 3.88 (s, 3H, OCH$$_{3})$$, 3.62 (s, 3H, OCH$$_{3});\,^{13}$$C NMR (150 MHz, DMSO-$$d_{6})$$ $$\delta $$: 194.8, 172.8, 165.5, 164.4, 157.3 (d, $$J$$ = 239.1 Hz), 144.9, 139.2, 138.5, 137.5, 135.3, 135.1, 129.8, 129.5, 128.9, 128.5, 127.9, 127.8, 127.4, 127.0, 126.0 (d, $$J$$ = 9.2 Hz), 122.4, 121.5, 118.5, 115.6 (d, $$J$$ = 17.0 Hz), 115.0 (d, $$J$$ = 18.6 Hz), 113.9, 109.6, 64.3, 58.1, 53.1, 52.3, 48.7, 43.3; IR (KBr) $$\upsilon $$: 3450, 2949, 1712, 1634, 1570, 1491, 1454, 1431, 1405, 1382, 1351, 1245, 1178, 1092, 967, 852, 820, 776 cm$$^{-1}$$; MS ($$m$$/$$z)$$: HRMS (ESI) Calcd. for C$$_{38}$$H$$_{29}$$ClFN$$_{2}$$O$$_{6}$$ ([M+H]$$^{+})$$: 663.1693. Found: 663.1692.

#### Dimethyl 1-butyl-5-chloro-3$^\prime$-(4-methoxybenzoyl)-2-oxo- 3$^\prime$,4a$^\prime$-dihydrospiro[indoline-3,4$^\prime$-pyrido[1,2-a]quinoline]- 1$^\prime$,2$^\prime$-dicarboxylate (***3e***)

Yellow solid, 60 %, m.p. 186–189 $$^{\circ }$$C; $$^{1}$$H NMR (600 MHz, DMSO-$$d_{6}) \quad \delta $$: 7.34 (br s, 2H, ArH), 7.22 (br s, 1H, ArH), 7.05 (br s, 1H, ArH), 6.93 (brs, 1H, ArH), 6.87 (br s, 4H, ArH), 6.76 (br s, 1H, ArH), 6.62 (br s, 1H, ArH), 6.35 (br s, 1H, CH), 5.44 (s, 1H, CH), 5.32–5.29 (m, 2H, CH), 4.59 (s, 1H, CH), 3.88 (s, 3H, OCH$$_{3})$$, 3.76 (s, 3H, OCH$$_{3})$$, 3.61 (s, 3H, OCH$$_{3})$$, 3.44 (brs, 2H, CH), 1.03–1.02 (m, 4H, CH), 0.76 (brs, 3H, CH$$_{3});\,^{13}$$C NMR (150 MHz, DMSO-$$d_{6})$$ $$\delta $$: 194.1, 172.5, 165.5, 164.6, 163.2, 144.9, 142.2, 137.6, 129.9, 129.8, 129.5, 128.1, 128.0, 127.8, 126.5, 125.3, 122.2, 121.4, 118.9, 118.4, 113.8, 113.7, 109.4, 64.0, 58.3, 55.4, 53.0, 52.2, 48.9, 28.7, 19.4, 13.5; IR (KBr) $$\upsilon $$: 3452, 2947, 1739, 1716, 1680, 1603, 1572, 1490, 1434, 1381, 1355, 1309, 1252, 1210, 1179, 1135, 1022, 968, 883, 816, 779 cm$$^{-1}$$; MS ($$m$$/$$z)$$: HRMS (ESI) Calcd. for C$$_{36}$$H$$_{34}$$ClN$$_{2}$$O$$_{7}$$ ([M+H]$$^{+})$$: 641.2049. Found: 649.2045.

#### 3$^\prime$-Ethyl 1$^\prime$,2$^\prime$-dimethyl 1-benzyl-5-methyl-2-oxo- 3$^\prime$,4a$^\prime$-dihydrospiro[indoline-3,4$^\prime$-pyrido-[1,2-a]quinoline]- 1$^\prime$,2$^\prime$,3$^\prime$-tricarboxylate (***3f***)

Yellow solid, 50 %, m.p. 117–115 $$^{\circ }$$C; $$^{1}$$H NMR (600 MHz, DMSO-$$d_{6}) \quad \delta $$: 7.38–7.18 (m, 6H, ArH), 6.94–6.82 (m, 5H, ArH), 6.52 (br s, 1H, ArH), 6.29 (br s, 1H, CH), 5.35 (b rs, 1H, CH), 5.02 (br s, 1H, CH), 4.88 (br s, 1H, CH), 4.50 (s, 1H, CH), 4.26 (s, 1H, CH), 3.87 (s, 3H, OCH$$_{3})$$, 3.70 (s, 3H, OCH$$_{3})$$, 3.35–3.54 (m, 2H, CH$$_{2})$$, 1.74 (s, 3H, CH$$_{3})$$, 0.38–0.37 (m, 3H, CH$$_{3});\,^{13}$$C NMR (150 MHz, DMSO-$$d_{6}) \quad \delta $$: 173.2, 168.1, 165.3, 164.7, 144.8, 141.1, 137.7, 136.1, 130.0, 129.4, 128.7, 128.5, 127.8, 127.6, 127.5, 127.4, 124.6, 122.1, 121.5, 118.9, 115.8, 114.0, 108.4, 63.9, 60.2, 58.2, 53.0, 52.2, 47.0, 43.3, 20.0, 12.8; IR (KBr) $$\upsilon $$: 3452, 2951, 1742, 1709, 1604, 1572, 1496, 1457, 1435, 1381, 1352, 1307, 1253, 1218, 1184, 1161, 1119, 1088, 1019, 981, 810, 776 cm$$^{-1}$$; MS ($$m$$/$$z)$$: HRMS (ESI) Calcd. for C$$_{35}$$H$$_{33}$$N$$_{2}$$O$$_{7}$$ ([M+H]$$^{+})$$: 593.2282. Found: 593.2285.

#### 3$^\prime$-Ethyl 1$^\prime$,2$^\prime$-dimethyl 1-benzyl-2-oxo-3$^\prime$,4a$^\prime$- dihydrospiro[indoline-3,4$^\prime$-pyrido[1,2-a]quino-line]- 1$^\prime$,2$^\prime$,3$^\prime$-tricarboxylate (***3g***)

Yellow solid, 63 %, m.p. 176–177 $$^{\circ }$$C; $$^{1}$$H NMR (600 MHz, DMSO-$$d_{6}) \quad \delta $$: 7.42–7.37 (m, 4H, ArH), 7.30 (br s, 1H, ArH), 7.17 (br s, 1H, ArH), 7.05 (brs, 1H, ArH), 6.92 (br s, 2H, ArH), 6.85–6.82 (m, 2H, ArH), 6.69 (br s, 1H, ArH), 6.52 (br s, 1H, ArH), 6.32 (br s, 1H, CH), 5.38 (br s, 1H, CH), 5.06 (d, $$J$$ = 15.0 Hz, 1H, CH), 4.90 (d, $$J$$ = 15.0 Hz, 1H, CH), 4.56 (s, 1H, CH), 4.28 (s, 1H, CH), 3.86 (s, 3H, OCH$$_{3})$$, 3.70 (s, 3H, OCH$$_{3})$$, 3.53 (br s, 1H, CH), 3.48 (brs, 1H, CH), 0.33 (brs, 3H, CH$$_{3});\,^{13}$$C NMR (150 MHz, DMSO-$$d_{6}) \quad \delta $$: 173.4, 168.1, 165.3, 164.6, 145.0, 143.6, 137.3, 136.0, 129.6, 128.8, 128.5, 127.7, 127.6, 127.5, 126.5, 124.7, 122.3, 121.4, 121.1, 118.9, 115.3, 113.9, 109.0, 64.1, 60.3, 58.0, 53.0, 52.2, 48.0, 43.3, 12.8; IR (KBr) $$\upsilon $$: 3452, 2950, 1740, 1707, 1610, 1571, 1493, 1463, 1435, 1384, 1357, 1307, 1282, 1252, 1221, 1177, 1132, 1087, 1020, 981, 902, 875, 831, 813, 783 cm$$^{-1}$$; MS ($$m$$/$$z)$$: HRMS (ESI) Calcd. for C$$_{34}$$H$$_{31}$$N$$_{2}$$O$$_{7}$$ ([M+H]$$^{+})$$: 579.2126. Found: 579.2131.

#### 3$^\prime$-Ethyl 1$^\prime$,2$^\prime$-dimethyl 1-benzyl-5-chloro-2-oxo- 3$^\prime$,4a$^\prime$-dihydrospiro[indoline-3,4$^\prime$-pyrido-[1,2-a]quinoline]- 1$^\prime$,2$^\prime$,3$^\prime$-tricarboxylate (***3h***)

Yellow solid, 70 %, m.p. 173–174 $$^{\circ }$$C; $$^{1}$$H NMR (600 MHz, DMSO-$$d_{6}) \quad \delta $$: 7.39–7.36 (m, 4H, ArH), 7.31–7.30 (m, 1H, ArH), 7.20 (t, $$J$$ = 7.8 Hz, 1H, ArH), 7.13 (d, $$J$$ = 8.4 Hz, 1H, ArH), 6.95–6.90 (m, 3H, ArH), 6.84 (d, $$J$$ = 7.2 Hz, 1H, ArH), 6.60 (brs, 1H, ArH), 6.35 (d, $$J$$ = 9.6 Hz, 1H, CH), 5.37 (dd, $$J_{1}$$ = 9.6 Hz, $$J_{2}$$ = 3.6 Hz, 1H, CH), 5.07 (d, $$J$$ = 15.6 Hz, 1H, CH), 4.90 (d, $$J$$ = 15.6 Hz, 1H, CH), 4.58 (s, 1H, CH), 4.29 (s, 1H, CH), 3.87 (s, 3H, OCH$$_{3})$$, 3.71 (s, 3H, OCH$$_{3})$$, 3.61–3.56 (m, 2H, CH$$_{2})$$, 0.42 (t, $$J$$ = 7.2 Hz, 3H, CH$$_{3});\,^{13}$$C NMR (150 MHz, DMSO-$$d_{6}) \quad \delta $$: 172.9, 168.1, 165.2, 164.5, 144.6, 142.4, 137.3, 135.7, 129.8, 128.6, 128.0, 127.7, 127.6, 126.9, 126.5, 125.5, 122.4, 121.3, 118.5, 115.7, 113.7, 110.3, 63.7, 60.5, 58.3, 53.1, 52.3, 47.8, 43.4, 12.8; IR (KBr) $$\upsilon $$: 3448, 2949, 1735, 1712, 1603, 1570, 1493, 1457, 1434, 1364, 1341, 1311, 1250, 1212, 1179, 1140, 1087, 1016, 977, 812, 775 cm$$^{-1}$$; MS ($$m$$/$$z)$$: HRMS (ESI) Calcd. for C$$_{34}$$H$$_{30}$$ClN$$_{2}$$O$$_{7}$$ ([M+H]$$^{+})$$: 613.1736. Found: 613.1739.

#### 3$^\prime$-Ethyl 1$^\prime$,2$^\prime$-dimethyl 1-butyl-5-chloro-2-oxo-3$^\prime$,4a$^\prime$-dihydrospiro[indoline-3,4$^\prime$-pyrido[1,2-a] quinoline]-1$^\prime$,2$^\prime$,3$^\prime$-tricarboxylate (***3i***)

Yellow solid, 73 %, m.p. 158–159 $$^{\circ }$$C; $$^{1}$$H NMR (600 MHz, DMSO-$$d_{6}) \quad \delta $$: 7.20–7.18 (m, 2H, ArH), 7.05 (d, $$J$$ = 7.8 Hz, 1H, ArH), 6.92–6.91 (m, 2H, ArH), 6.86–6.85 (m, 1H, ArH), 6.65 (br s, 1H, ArH), 6.38 (d, $$J$$ = 9.6 Hz, 1H, CH), 5.37 (d, $$J$$ = 6.6 Hz, 1H, CH), 4.52 (s, 1H, CH), 4.21 (s, 1H, CH), 3.87 (s, 3H, OCH$$_{3})$$, 3.74 (br s, 2H, CH$$_{2})$$, 3.69 (s, 3H, OCH$$_{3})$$, 3.63–3.62 (m, 2H, CH$$_{2})$$, 1.57 (brs, 2H, CH$$_{2})$$, 1.35–1.34 (m, 2H, CH$$_{2})$$, 0.93 (br s, 3H, CH$$_{3})$$, 0.61 (brs, 3H, CH$$_{3});\,^{13}$$C NMR (150 MHz, DMSO-$$d_{6}) \quad \delta $$: 172.6, 172.6, 168.1, 165.2, 164.5, 144.6, 142.8, 137.4, 129.8, 128.6, 127.9, 127.6, 126.9, 126.6, 125.2, 122.4, 121.3, 118.5, 115.6, 113.8, 109.9, 63.5, 60.4, 58.2, 53.0, 52.2, 47.9, 29.0, 19.4, 13.5, 13.1; IR (KBr) $$\upsilon $$: 3454, 2956, 1744, 1713, 1639, 1614, 1598, 1570, 1490, 1433, 1378, 1355, 1325, 1303, 1247, 1212, 1183, 1135, 1116, 1021, 989, 969, 944, 914, 868, 822, 780 cm$$^{-1}$$; MS ($$m$$/$$z)$$: HRMS (ESI) Calcd. for C$$_{31}$$H$$_{32}$$ClN$$_{2}$$O$$_{7}$$ ([M+H]$$^{+})$$: 579.1893. Found: 579.1894.

#### 3$^\prime$-Ethyl 1$^\prime$,2$^\prime$-dimethyl 1-benzyl-5-fluoro-2-oxo-3$^\prime$,4a$^\prime$- dihydrospiro[indoline-3,4$^\prime$-pyrido-[1,2-a]quinoline]- 1$^\prime$,2$^\prime$,3$^\prime$-tricarboxylate (***3j***)

Yellow solid, 65 %, m.p. 160–163 $$^{\circ }$$C; $$^{1}$$H NMR (600 MHz, DMSO-$$d_{6}) \quad \delta $$: 7.40–7.37 (m, 4H, ArH), 7.30 (br s, 1H, ArH), 7.20 (br s, 1H, ArH), 7.94–7.63 (m, 4H, ArH), 6.85 (br s, 1H, ArH), 6.43 (br s, 1H, ArH), 6.36 (d, $$J$$ = 8.4 Hz, 1H, CH), 5.40 (br s, 1H, CH), 5.08 (d, $$J$$ = 15.6 Hz, 1H, CH), 4.90 (d, $$J$$ = 15.6 Hz, 1H, CH), 4.60 (s, 1H, CH), 4.30 (s, 1H, CH), 3.87 (s, 3H, OCH$$_{3})$$, 3.70 (s, 3H, OCH$$_{3})$$, 3.59–3.55 (m, 2H, CH$$_{2})$$, 0.38 (brs, 3H, CH$$_{3});\,^{13}$$C NMR (150 MHz, CDCl$$_{3}) \quad \delta $$: 174.0, 168.6, 165.8, 165.1, 158.1 (d, $$J$$ = 239.6 Hz), 145.6, 139.7, 137.9, 135.3, 129.8, 128.8, 128.2, 128.0, 127.9, 127.6, 127.1 (d, $$J$$ = 7.8 Hz), 122.5, 121.6, 118.3, 115.9, 115.6 (d, $$J$$ = 26.0 Hz), 115.0 (d, $$J$$ = 23.3 Hz), 114.4, 109.0 (d, $$J$$ = 8.0 Hz), 64.5, 60.9, 59.3, 53.1, 52.2, 48.4, 44.4, 13.3; IR (KBr) $$\upsilon $$: 3451, 2950, 1739, 1708, 1608, 1571, 1494, 1456, 1436, 1344, 1307, 1252, 1226, 1175, 1131, 1020, 979, 900, 875, 827, 775 cm$$^{-1}$$; MS ($$m$$/$$z)$$: HRMS (ESI) Calcd. for C$$_{34}$$H$$_{30}$$FN$$_{2}$$O$$_{7}$$ ([M+H]$$^{+})$$: 597.2032. Found: 597.2034.

### General procedure for the Diels–Alder reaction of spiro[indoline-3,1$^\prime$-quinolizines]

A mixture of spiro[indoline-3,1$^\prime$-quinolizine] (1.0 mmol) and $N$-substituted maleimide or maleic anhydride (1.5 mmol) in 10.0 mL of DME was refluxed for 12 h. Then, the solvent was removed by evaporation and the residue was subjected to thin-layer chromatography with a mixture of light petroleum and ethyl acetate (V/V = 2:1) as the developing reagent to give the pure spiro compound **4a**–**4g**.

#### Spiro compounds (***4a***)

White solid, 80 %, m.p. 297–298 $$^{\circ }$$C; $$^{1}$$H NMR (600 MHz, DMSO-$$d_{6}) \quad \delta $$: 7.60 (d, $$J$$ = 8.4 Hz, 2H, ArH), 7.44 (t, $$J$$ = 7.8 Hz, 2H, ArH), 7.38 (t, $$J$$ = 7.2 Hz, 1H, ArH), 7.19 (t, $$J$$ = 7.2 Hz, 1H, ArH), 7.14 (t, $$J$$ = 7.2 Hz, 2H, ArH), 7.05 (t, $$J$$ = 8.4 Hz, 1H, ArH), 6.99–6.97 (m, 6H, ArH), 6.79 (dd, $$J_{1}$$ = 9.0 Hz, $$J_{2}$$ = 4.2 Hz, 1H, ArH), 6.61–6.60 (m, 1H, ArH), 5.77 (d, $$J$$ = 5.4 Hz, 1H, CH), 5.20 (s, 1H, CH), 4.59 (br s, 2H, CH$$_{2})$$, 4.32–4.31 (m, 1H, CH), 4.15 (s, 1H, CH), 4.00 (s, 3H, OCH$$_{3})$$, 3.84 (s, 3H, OCH$$_{3})$$, 3.54 (dd, $$J_{1}$$ = 7.5 Hz, $$J_{2}$$ = 3.0 Hz, 1H, CH), 3.48 (dd, $$J_{1}$$ = 7.5 Hz, $$J_{2}$$ = 3.0 Hz, 1H, CH), 3.34 (br s, 3H, OCH$$_{3})$$, 2.66 (s, 1H, CH), 0.52 (s, 3H, CH$$_{3});\,^{13}$$C NMR (150 MHz, DMSO-$$d_{6}) \quad \delta $$: 194.6, 175.1, 174.5, 174.1, 165.3, 164.5, 163.1, 157.7 (d, $$J$$ = 236.4 Hz), 146.5, 140.6, 138.5, 135.6, 131.8, 130.1, 129.8, 129.0, 128.6, 128.5, 127.5, 127.4, 126.6, 126.0 (d, $$J$$ = 9.5 Hz), 122.6, 117.2 (d, $$J$$ = 24.8 Hz), 114.6 (d, $$J$$ = 23.6 Hz), 113.5, 109.8 (d, $$J$$ = 7.2 Hz), 94.6, 60.7, 55.5, 53.3, 51.9, 50.8, 49.8, 45.9, 43.1, 42.2, 38.5, 18.9; IR (KBr) $$\upsilon $$: 3450, 2951, 1778, 1711, 1670, 1590, 1488, 1459, 1434, 1376, 1345, 1324, 1244, 1176, 1123, 1033, 987, 950, 903, 843, 811 cm$$^{-1}$$; MS ($$m$$/$$z)$$: HRMS (ESI) Calcd. for C$$_{46}$$H$$_{39}$$FN$$_{3}$$O$$_{9}$$ ([M+H]$$^{+})$$: 796.2665. Found: 796.2670.

#### Spiro compounds (***4b***)

White solid, 77 %, m.p. $$>$$300 $$^{\circ }$$C; $$^{1}$$H NMR (600 MHz, DMSO-$$d_{6}) \quad \delta $$: 7.49 (d, $$J$$ = 7.8 Hz, 2H, ArH), 7.43 (t, $$J$$ = 7.8 Hz, 2H, ArH), 7.38 (t, $$J$$ = 7.8 Hz, 1H, ArH), 7.26–7.25 (m, 3H, ArH), 7.19 (t, $$J$$ = 7.2 Hz, 1H, ArH), 7.14 (t, $$J$$ = 7.2 Hz, 2H, ArH), 7.00–6.99 (m, 4H, ArH), 6.83–6.82 (m, 2H, ArH), 5.74 (d, $$J$$ = 5.4 Hz, 1H, CH), 5.21 (s, 1H, CH), 4.54 (br s, 2H, CH$$_{2})$$, 4.32–4.30 (m, 1H, CH), 4.15 (s, 1H, CH), 4.00 (s, 3H, OCH$$_{3})$$, 3.54 (dd, $$J_{1}$$ = 7.5 Hz, $$J_{2}$$ = 3.0 Hz, 1H, CH), 3.47 (dd, $$J_{1}$$ = 7.5 Hz, $$J_{2}$$ = 3.0 Hz, 1H, CH), 3.35 (s, 3H, OCH$$_{3})$$, 2.64 (s, 1H, CH), 2.37 (s, 3H, CH$$_{3})$$, 0.50 (s, 3H, CH$$_{3});\,^{13}$$C NMR (150 MHz, CDCl$$_{3}) \quad \delta $$: 196.0, 175.0, 174.7, 173.8, 166.0, 165.1, 146.8, 143.7, 141.7, 140.9, 135.0, 134.7, 131.3, 131.1, 129.3, 129.0, 128.9, 128.4, 128.3, 128.0, 127.4, 126.6, 126.1, 109.8, 96.5, 61.6, 53.7, 52.1, 51.3, 50.4, 46.0, 44.2, 42.6, 39.2, 21.6, 19.4; IR (KBr) $$\upsilon $$: 3452, 2952, 1781, 1712, 1641, 1483, 1430, 1384, 1322, 1233, 1186, 1129, 949, 887, 803 cm$$^{-1}$$; MS ($$m$$/$$z)$$: HRMS (ESI) Calcd. for C$$_{46}$$H$$_{39}$$ClN$$_{3}$$O$$_{8}$$ ([M+H]$$^{+})$$: 796.2420. Found: 796.2429.

#### Spiro compounds (***4c***)

White solid, 72 %, m.p. 173–175 $$^{\circ }$$C; $$^{1}$$H NMR (600 MHz, DMSO-$$d_{6}) \quad \delta $$: 7.46 (d, $$J$$ = 8.4 Hz, 2H, ArH), 7.22 (d, $$J$$ = 7.8 Hz, 2H, ArH), 7.09 (t, $$J$$ = 8.4 Hz, 1H, ArH), 6.91–6.86 (m, 5H, ArH), 6.57 (d, $$J$$ = 6.6 Hz, 1H, ArH), 5.81 (d, $$J$$ = 4.2 Hz, 1H, CH), 5.09 (s, 1H, CH), 4.31 (br s, 1H, CH), 4.04 (s, 1H, CH), 4.00 (s, 3H, OCH$$_{3})$$, 3.80 (s, 3H, OCH$$_{3})$$, 3.53–3.52 (m, 1H, CH), 3.46–3.45 (m, 1H, CH), 3.37 (brs, 1H, CH), 3.28 (s, 3H, OCH$$_{3})$$, 3.26 (br s, 1H, CH), 2.61 (s, 1H, CH), 2.29 (s, 3H, CH$$_{3})$$, 1.04–1.01 (m, 3H, CH), 0.84–0.82 (m, 1H, CH), 0.74–0.71 (m, 6H, CH$$_{3});\,^{13}$$C NMR (150 MHz, DMSO-$$d_{6}) \quad \delta $$: 196.1, 173.7, 170.9, 169.5, 165.3, 164.5, 157.2 (d, $$J$$ = 235.2 Hz), 146.3, 143.3, 139.3, 134.6, 138.7, 127.7, 124.5 (d, $$J$$ = 8.9 Hz), 116.4 (d, $$J$$ = 25.2 Hz), 114.8 (d, $$J$$ = 23.1 Hz), 109.3 (d, $$J$$ = 8.9 Hz), 95.1, 93.6, 58.4, 55.1, 53.4, 51.6, 50.9, 49.9, 47.8, 47.5, 43.0, 37.5, 28.5, 21.0, 19.2, 13.5; IR (KBr) $$\upsilon $$: 3457, 2956, 1712, 1600, 1514, 1489, 1455, 1386, 1322, 1237, 1178, 1131, 1025, 962, 808, 758 cm$$^{-1}$$; MS ($$m$$/$$z)$$: HRMS (ESI) Calcd. for C$$_{44}$$H$$_{42}$$FN$$_{3}$$NaO$$_{9}$$ ([M+Na]$$^{+})$$: 798.2797. Found: 798.2783.

#### Spiro compounds (***4d***)

White solid, 85 %, m.p. 182–185 $$^{\circ }$$C; $$^{1}$$H NMR (600 MHz, DMSO-$$d_{6}) \quad \delta $$: 7.51 (d, $$J$$ = 8.4 Hz, 2H, ArH), 7.46 (d, $$J$$ = 8.4 Hz, 2H, ArH), 7.11–7.08 (m, 1H, ArH), 7.05 (d, $$J$$ = 8.4 Hz, 2H, ArH), 6.91–6.90 (m, 3H, ArH), 6.57 (d, $$J$$ = 8.4 Hz, 1H, ArH), 5.82 (d, $$J$$ = 1.8 Hz, 1H, CH), 5.09 (s, 1H, CH), 4.32 (br s, 1H, CH), 4.06 (s, 1H, CH), 4.00 (s, 3H, OCH$$_{3})$$, 3.80 (s, 3H, OCH$$_{3})$$, 3.55–3.54 (m, 1H, CH), 3.48–3.47 (m, 1H, CH), 3.35 (br s, 5H, CH, OCH$$_{3})$$, 2.62 (s, 1H, CH), 1.03 (br s, 3H, CH), 0.82 (brs, 1H, CH), 0.73–0.72 (m, 6H, CH$$_{3});\,^{13}$$C NMR (150 MHz, CDCl$$_{3}) \quad \delta $$: 195.0, 174.7, 174.6, 173.6, 166.0, 165.1, 163.4, 158.5 (d, $$J$$ = 239.9 Hz), 146.6, 141.7, 138.8, 134.7, 130.4, 129.8, 129.4, 127.4, 126.2 (d, $$J$$ = 8.6 Hz), 123.0, 118.8 (d, $$J$$ = 25.8 Hz), 115.1 (d, $$J$$ = 23.1 Hz), 113.4, 108.4 (d, $$J$$ = 8.4 Hz), 96.6, 61.2, 55.4, 53.5, 51.9, 51.2, 50.5, 47.6, 46.0, 42.6, 40.2, 39.3, 29.0, 20.1, 19.7, 13.6; IR (KBr) $$\upsilon $$: 3458, 2957, 1781, 1714, 1677, 1598, 1491, 1455, 1384, 1325, 1240, 1280, 1130, 1093, 1020, 960, 908, 868, 835, 807 cm$$^{-1}$$; MS ($$m$$/$$z)$$: HRMS (ESI) Calcd. for C$$_{43}$$H$$_{40}$$ClFN$$_{3}$$O$$_{9}$$ ([M+H]$$^{+})$$: 796.2432. Found: 796.2432.

#### Spiro compounds (***4e***)

White solid, 86 %, m.p. 230–233 $$^{\circ }$$C; $$^{1}$$H NMR (600 MHz, DMSO-$$d_{6}) \quad \delta $$: 7.53–7.49 (m, 4H, ArH), 7.09–7.06 (m, 1H, ArH), 7.04 (d, $$J$$ = 8.4 Hz, 2H, ArH), 6.92–6.90 (m, 3H, ArH), 6.60 (d, $$J$$ = 8.4 Hz, 1H, ArH), 5.13 (s, 1H, CH), 4.63 (d, $$J$$ = 4.2 Hz, 1H, CH), 4.05 (br s, 1H, CH), 4.02 (s, 3H, OCH$$_{3})$$, 3.99 (s, 1H, CH), 3.80 (s, 3H, OCH$$_{3})$$, 3.58–3.56 (m, 1H, CH), 3.48–3.43 (m, 1H, CH), 3.40–3.39 (m, 1H, CH), 3.36 (s, 3H, OCH$$_{3})$$, 3.32 (br s, 1H, CH), 2.75 (s, 1H, CH), 1.46 (s, 3H, CH$$_{3})$$, 1.03–1.01 (m, 1H, CH), 0.93–0.91 (m, 2H, CH), 0.80–0.79 (m, 1H, CH), 0.67 (d, $$J$$ = 7.2 Hz, 3H, CH$$_{3});\,^{13}$$C NMR (150 MHz, CDCl$$_{3}) \quad \delta $$: 195.2, 175.1, 174.4, 173.4, 166.1, 165.2, 163.5, 158.3 (d, $$J$$ = 240.2 Hz), 146.6, 139.6, 138.0, 134.7, 130.6, 130.2, 129.8, 129.4, 127.3, 127.2 (d, $$J$$ = 8.9 Hz), 122.3, 117.4 (d, $$J$$ = 25.5 Hz), 115.0 (d, $$J$$ = 23.4 Hz), 113.4, 108.4 (d, $$J$$ = 8.0 Hz), 97.0, 61.8, 55.7, 55.4, 53.5, 51.3, 50.4, 46.6, 45.0, 42.7, 40.2, 34.6, 29.0, 20.0, 19.5, 13.6; IR (KBr) $$\upsilon $$: 3457, 2958, 1747, 1712, 1601, 1492, 1446, 1426, 1384, 1355, 1318, 1264, 1236, 1175, 1138, 1022, 956, 918, 896, 876, 825, 803 cm$$^{-1}$$; MS ($$m$$/$$z)$$: HRMS (ESI) Calcd. for C$$_{43}$$H$$_{30}$$ClFN$$_{3}$$O$$_{9}$$ ([M+H]$$^{+})$$: 796.2432. Found: 796.2429.

#### Spiro compounds (***4f***)

White solid, 90 %, m.p. $$>$$300 $$^{\circ }$$C; $$^{1}$$H NMR (600 MHz, DMSO-$$d_{6}) \quad \delta $$: 7.43 (d, $$J$$ = 4.8 Hz, 2H, ArH), 7.27 (d, $$J$$ = 7.2 Hz, 1H, ArH), 6.90 (br s, 3H, ArH), 6.84 (br s, 1H, ArH), 5.50 (s, 1H, CH), 4.89 (d, $$J$$ = 3.0 Hz, 1H, CH), 4.47 (brs, 1H, CH), 4.00 (s, 3H, OCH$$_{3})$$, 3.98 (s, 1H, CH), 3.79 (s, 3H, OCH$$_{3})$$, 3.77–3.75 (m, 1H, CH), 3.69 (br s, 1H, CH), 3.36 (br s, 4H, CH, OCH$$_{3})$$, 3.26–3.24 (m, 1H, CH), 2.74 (s, 3H, OCH$$_{3})$$, 2.54 (s, 1H, CH), 1.00 (br s, 3H, CH), 0.82–0.78 (m, 1H, CH), 0.70 (br s, 3H, CH$$_{3});$$ IR (KBr) $$\upsilon $$: 3453, 2957, 1861, 1779, 1699, 1675, 1641, 1584, 1511, 1483, 1455, 1433, 1368, 1342, 1321, 1269, 1232, 1203, 1170, 1129, 1086, 1049, 1017, 972, 942, 924, 879, 841, 811 cm$$^{-1}$$; MS ($$m$$/$$z)$$: HRMS (ESI) Calcd. for C$$_{37}$$H$$_{35}$$ClN$$_{2}$$NaO$$_{11}$$ ([M+Na]$$^{+})$$: 741.1822. Found: 741.1808.

#### Spiro compounds (***4g***)

White solid, 88 %, m.p. 283–285 $$^{\circ }$$C; $$^{1}$$H NMR (600 MHz, DMSO-$$d_{6}) \quad \delta $$: 7.30 (d, $$J$$ = 8.4 Hz, 2H, ArH), 7.17 (d, $$J$$ = 7.8 Hz, 2H, ArH), 7.08 (td, $$J_{1}$$ = 9.3 Hz, $$J_{2}$$ = 2.4 Hz, 1H, ArH), 6.88 (dd, $$J_{1}$$ = 8.4 Hz, $$J_{2}$$ = 4.2 Hz, 1H, ArH), 6.61 (dd, $$J_{1}$$ = 9.3 Hz, $$J_{2}$$ = 2.4 Hz, 1H, ArH), 5.06 (s, 1H, CH), 4.92 (dd, $$J_{1}$$ = 6.6 Hz, $$J_{2}$$ = 2.4 Hz, 1H, CH), 4.48 (dd, $$J_{1}$$ = 6.6 Hz, $$J_{2}$$ = 3.6 Hz, 1H, CH), 4.00–3.99 (m, 4H, CH, OCH$$_{3})$$, 3.76 (dd, $$J_{1}$$ = 7.5 Hz, $$J_{2}$$ = 3.6 Hz, 1H, CH), 3.67 (dd, $$J_{1}$$ = 7.5 Hz, $$J_{2}$$ = 3.6 Hz, 1H, CH), 3.36 (s, 3H, OCH$$_{3})$$, 3.28–3.27 (m, 1H, CH), 3.20–3.19 (m, 1H, CH), 2.71 (s, 3H, OCH$$_{3})$$, 2.56 (s, 1H, CH), 2.31 (s, 3H, CH$$_{3})$$, 1.02 (br s, 3H, CH), 0.81–0.79 (m, 1H, CH), 0.71 (d, $$J$$ = 7.2 Hz, 3H, CH$$_{3});\,^{13}$$C NMR (150 MHz, DMSO-$$d_{6}) \quad \delta $$: 194.8, 175.3, 174.2, 174.1, 165.4, 164.6, 163.1, 157.5 (d, $$J$$ = 236.1 Hz), 146.5, 140.6, 139.0, 138.2, 130.0, 129.8, 129.4, 129.1, 126.3, 125.9 (d, $$J$$ = 8.3 Hz), 122.7, 117.3 (d, $$J$$ = 22.5 Hz), 114.7 (d, $$J$$ = 23.3 Hz), 113.4, 109.3, 94.5, 60.2, 55.4, 53.3, 51.8, 50.8, 49.7, 47.0, 45.8, 42.1, 28.7, 20.6, 19.4, 19.2, 13.4; IR (KBr) $$\upsilon $$: 3456, 2956, 1864, 1780, 1740, 1697, 1641, 1585, 1489, 1455, 1435, 1382, 1339, 1320, 1267, 1232, 1202, 1177, 1120, 1086, 1046, 1017, 979, 929, 872, 816, 792 cm$$^{-1}$$; MS ($$m$$/$$z)$$: HRMS (ESI) Calcd. for C$$_{37}$$H$$_{35}$$FN$$_{2}$$NaO$$_{10}$$ ([M+Na]$$^{+})$$: 709.2168. Found: 709.2158.

## Supporting information

$^{1}$H and $^{13}$C NMR spectra and 2D NMR for all new compounds are available. Crystallographic data **1c** (CCDC 916455), **1h** (CCDC 916456), **1m** (CCDC 916457), **2b **(CCDC 916458), **3e** (CCDC 928874), **3i** (CCDC 928875), **4d** (CCDC 928873) have been deposited at the Cambridge Crystallographic Database Centre and are available on request (http://www.ccdc.cam.ac.uk).

## Electronic supplementary material

Below is the link to the electronic supplementary material.

Supplementary material 1 (doc 4383 KB)
