# Supplementary material for: Synthesis of spiro[indoline-3,1′-quinolizines] and spiro[indoline-3,4′-pyrido[1,2-a]quinolines] via three-component reactions of azaarenes, acetylenedicarboxylate, and 3-methyleneoxindoles
Source: Mol Divers. 2013 Jul 19;17(4):627–39. doi: 10.1007/s11030-013-9459-5 (PMC3836201; doi:10.1007/s11030-013-9459-5)

**Synthesis of spiro[indoline-3,1’-quinolizines] and spiro[indoline-3,4'-pyrido[1,2-a]quinolines] via three-component reactions of azaarenes, acetylenedicarboxylate and 3-methyleneoxindoles**

Jing Sun, Hui Gong, Yan Sun, Chao-Guo Yan*

**Supporting Information**

Figure s1-s3 2

General Experimental Methods and

Characterization of compounds 3-36

**X-Ray Crystallographic Data: CIF in separate file.**

Crystallographic data **1c** (CCDC 916455), **1h** (CCDC 916456), **1m** (CCDC 916457), **2b** (CCDC 916458), **3e** (CCDC 928874), **3i** (CCDC 928875), **4d** (CCDC 928873) have been deposited vat the Cambridge Crystallographic Database Centre and is available on request from the Director, CCDC, 12 Union Road, Cambridge, CB2 1EZ, UK (http//www.ccdc.cam.ac.uk).

**Figure s1 molecular Structure of compound 1h**

**Figure s2 molecular Structure of compound 1m**

**Figure s3 molecular Structure of compound 2a**

**dimethyl 1-benzyl-6'-methyl-2'-(4-methylbenzoyl)-2-oxo-2',9a'-dihydrospiro[indoline-3,1'-**

**quinolizine]-3',4'-dicarboxylate (1a)**: yellow solid, 66%, m.p. 173~175℃; ^1^H NMR (600 MHz, DMSO-*d_6_*) δ: 7.26 (brs, 2H, ArH), 7.20 (brs, 2H, ArH), 7.15~7.13 (m, 4H, ArH), 7.06 (brs, 2H, ArH), 6.89 (brs, 3H, ArH), 6.58 (brs, 1H, CH), 5.60 (brs, 1H, CH), 5.27 (s, 1H, CH), 4.98 (brs, 1H, CH), 4.61 (brs, 2H, CH), 4.50 (brs, 1H, CH), 3.87 (s, 3H, OCH_3_), 3.56 (s, 3H, OCH_3_), 2.32 (s, 3H, CH_3_), 1.93 (s, 3H, CH_3_); ^13^C NMR (150 MHz, CDCl_3_) δ: 196.5, 174.2, 166.3, 166.1, 146.7, 143.4, 136.8, 135.1, 134.9, 128.9, 128.6, 128.4, 128.2, 127.4, 127.1, 126.9, 125.4, 124.9, 122.1, 116.1, 113.9, 108.5, 103.1, 66.8, 58.4, 53.3, 52.2, 49.2, 43.9, 21.7, 20.6; IR (KBr) υ: 3447, 2946, 2025, 1732, 1710, 1685, 1655, 1611, 1578, 1489, 1465, 1437, 1405, 1368, 1291, 1232, 1177, 1129, 1080, 1017, 963, 815, 793, 738 cm^-1^; MS (*m*/*z*): HRMS (ESI) Calcd. for C_36_H_33_N_2_O_6_ ([M+H]^+^): 589.2347. Found: 589.2350.


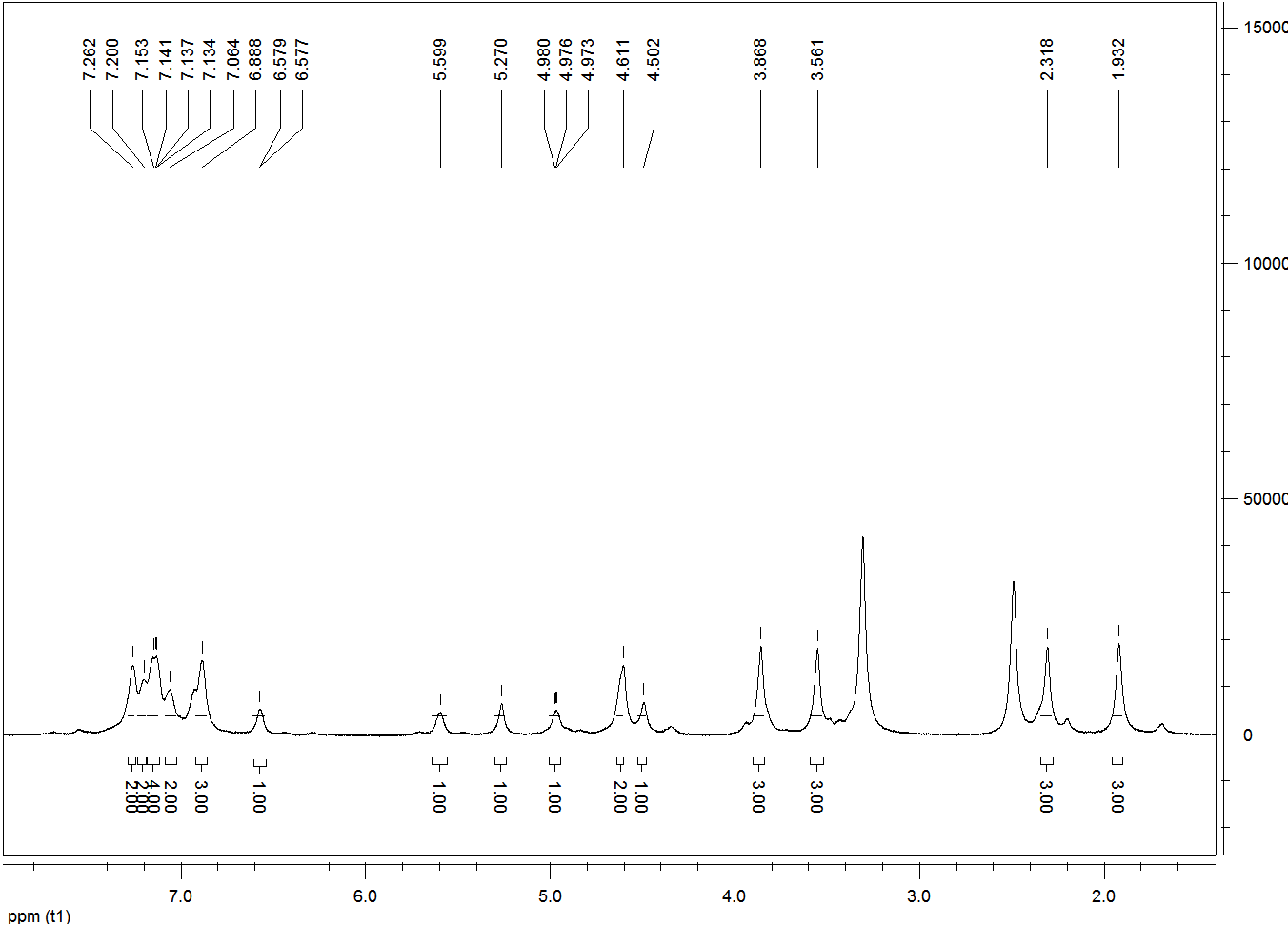


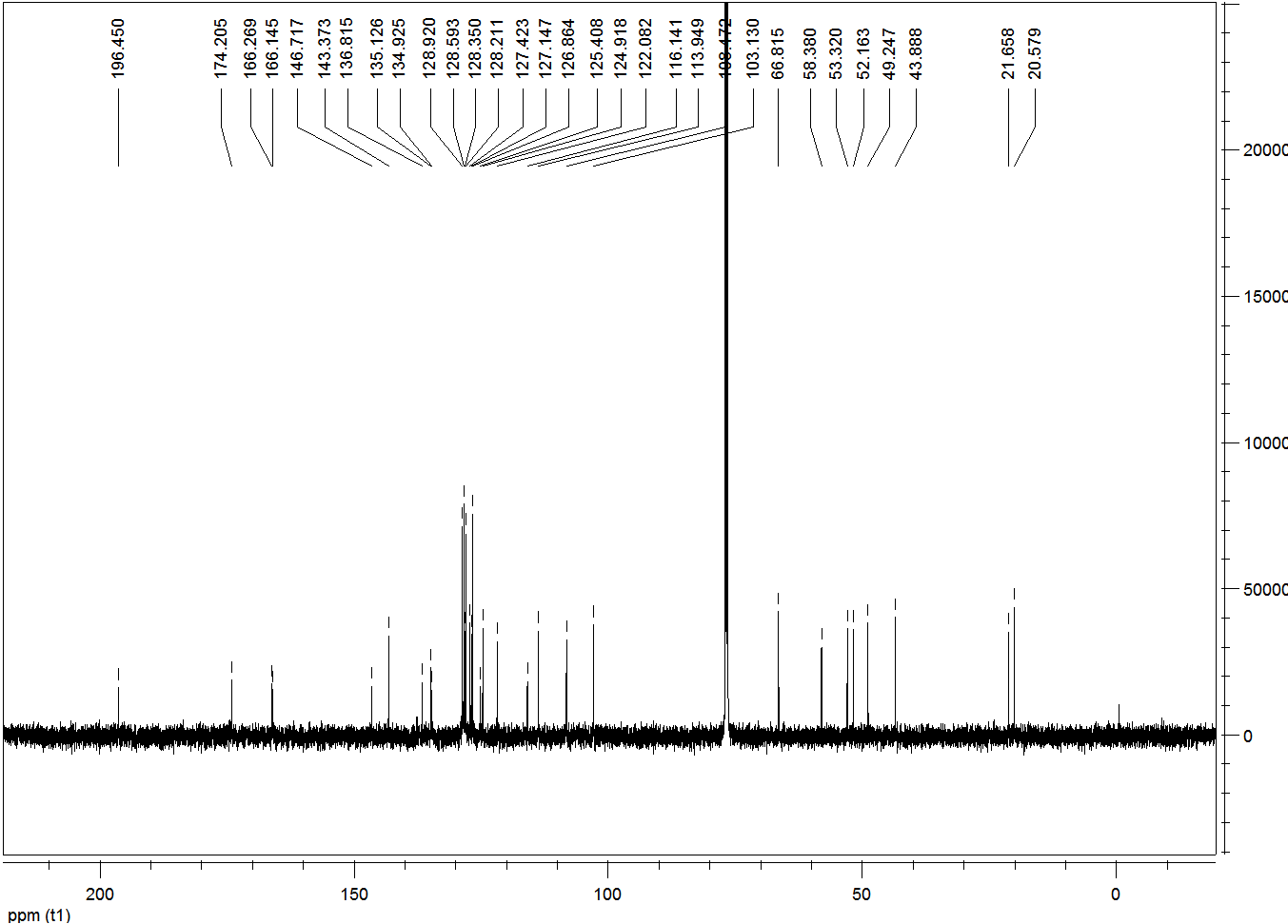


**dimethyl 1-butyl-5,6'-dimethyl-2'-(4-methylbenzoyl)-2-oxo-2',9a'-dihydrospiro[indoline-3,1'-**

**quinolizine]-3',4'-dicarboxylate (1b):** yellow solid, 53%, m.p. 161~163℃; ^1^H NMR (600 MHz, DMSO-*d_6_*) δ: 7.39 (d, *J* = 7.8Hz, 2H, ArH), 7.18 (d, *J* = 7.2Hz, 2H, ArH), 7.03~7.01 (m, 2H, ArH), 6.73 (d, *J* = 7.8Hz, 1H, ArH), 6.21 (d, *J* = 7.8Hz, 1H, CH), 5.50~5.47 (m, 1H, CH), 5.23 (s, 1H, CH), 4.93~4.91 (m, 1H, CH), 4.65 (d, *J* = 9.6Hz, 1H, CH), 3.95 (s, 3H, OCH_3_), 3.39 (s, 3H, OCH_3_), 3.24~3.21 (m, 1H, CH), 3.08~3.04 (m, 1H, CH), 2.32 (s, 3H, CH_3_), 2.24 (s, 3H, CH_3_), 1.67 (s, 3H, CH_3_), 0.95 (brs, 3H, CH), 0.69 (t, *J* = 7.2Hz, 3H, CH_3_); ^13^C NMR (150 MHz, DMSO-*d_6_*) δ: 196.9, 172.8, 165.1, 164.3, 144.4, 143.2, 139.4, 130.6, 128.9, 128.6, 128.0, 127.8, 127.7, 126.7, 121.1, 121.0, 107.9, 101.9, 63.7, 53.3, 53.0, 51.3, 44.5, 28.4, 21.1, 21.0, 19.4, 13.5; IR (KBr) υ: 3452, 2952, 2869, 2025, 1747, 1698, 1659, 1615, 1582, 1496, 1436, 1362, 1269, 1237, 1151, 1115, 1083, 1048, 936, 866, 827, 779, 740, 701 cm^-1^; MS (*m*/*z*): HRMS (ESI) Calcd. for C_34_H_37_N_2_O_6_ ([M+H]^+^): 569.2686. Found: 569.2671.


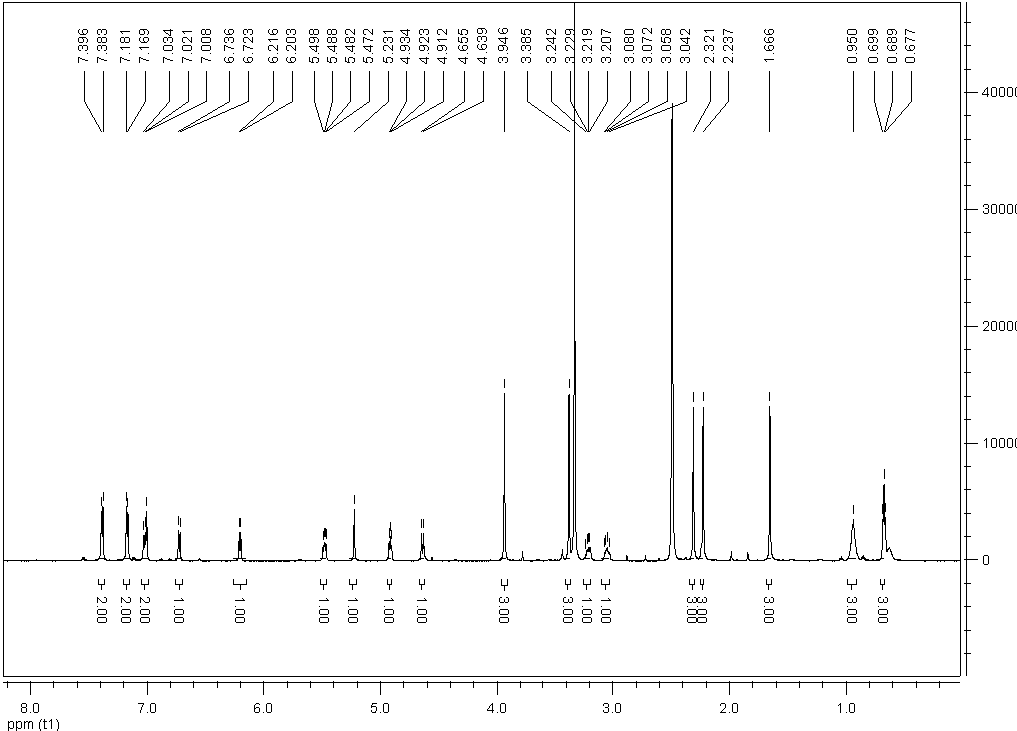


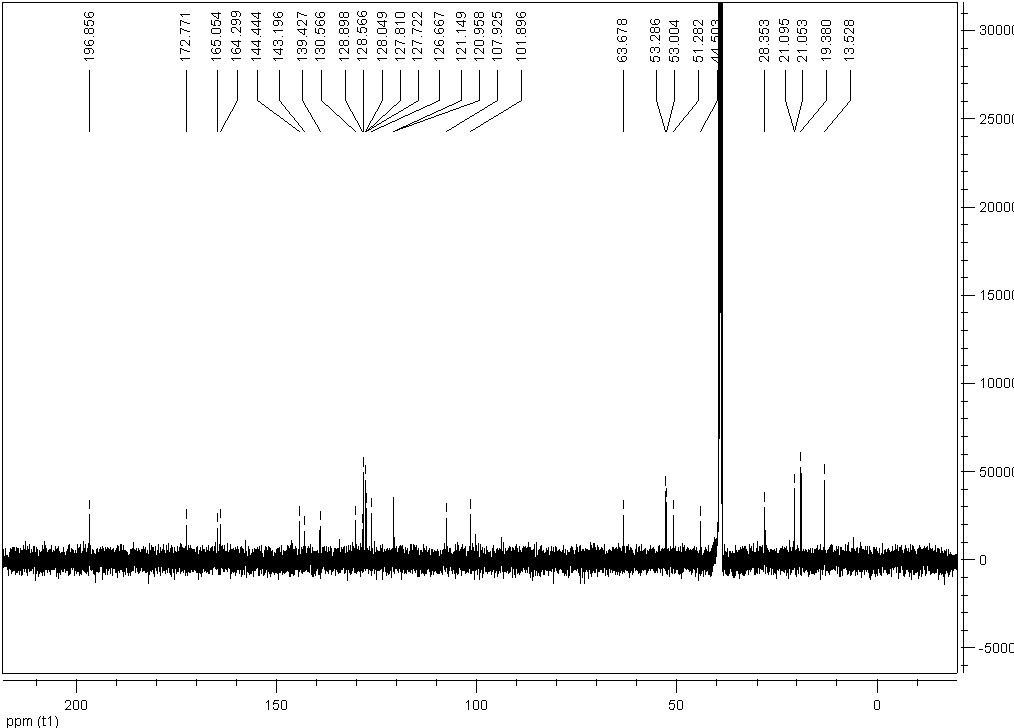


**dimethyl 1-benzyl-2'-(4-methoxybenzoyl)-7'-methyl-2-oxo-2',9a'-dihydrospiro[indoline-3,1'-**

**quinolizine]-3',4'-dicarboxylate (1c):** yellow solid, 61%, m.p. 190~192℃; ^1^H NMR (600 MHz, DMSO-*d_6_*) δ: 7.51 (d, *J* = 7.8Hz, 2H, ArH), 7.18 (t, *J* = 7.2Hz, 1H, ArH), 7.11 (t, *J* = 7.8Hz, 4H, ArH), 6.98~6.93 (m, 3H, ArH), 6.81 (d, *J* = 7.2Hz, 2H, ArH), 6.69 (d, *J* = 7.8Hz, 1H, ArH), 6.05 (s, 1H, CH), 5.54 (d, *J* = 8.4Hz, 1H, CH), 5.35 (s, 1H, CH), 4.91~4.90 (m, 2H, CH), 4.64 (d, *J* = 15.7Hz, 1H, CH), 4.49 (d, *J* = 15.7Hz, 1H, CH), 3.95 (s, 3H, OCH_3_), 3.82 (s, 3H, OCH_3_), 3.43 (s, 3H, OCH_3_), 1.43 (s, 3H, CH_3_); ^13^C NMR (150 MHz, CDCl_3_) δ: 195.8, 174.8, 166.1, 165.3, 163.3, 145.6, 142.6, 135.2, 130.5, 130.4, 128.6, 128.5, 128.2, 127.6, 127.5, 126.9, 125.9, 123.0, 122.5, 116.0, 113.4, 109.5, 108.6, 104.6, 62.7, 55.4, 54.1, 53.4, 51.6, 47.4, 43.9, 17.5; IR (KBr) υ: 3450, 2949, 2843, 2026, 1742, 1708, 1674, 1609, 1582, 1510, 1489, 1464, 1434, 1412, 1380, 1308, 1244, 1172, 1125, 1021, 983, 941, 899, 825, 776, 746 cm^-1^; MS (*m*/*z*): HRMS (ESI) Calcd. for C_36_H_33_N_2_O_7_ ([M+H]^+^): 605.2282. Found: 605.2290.


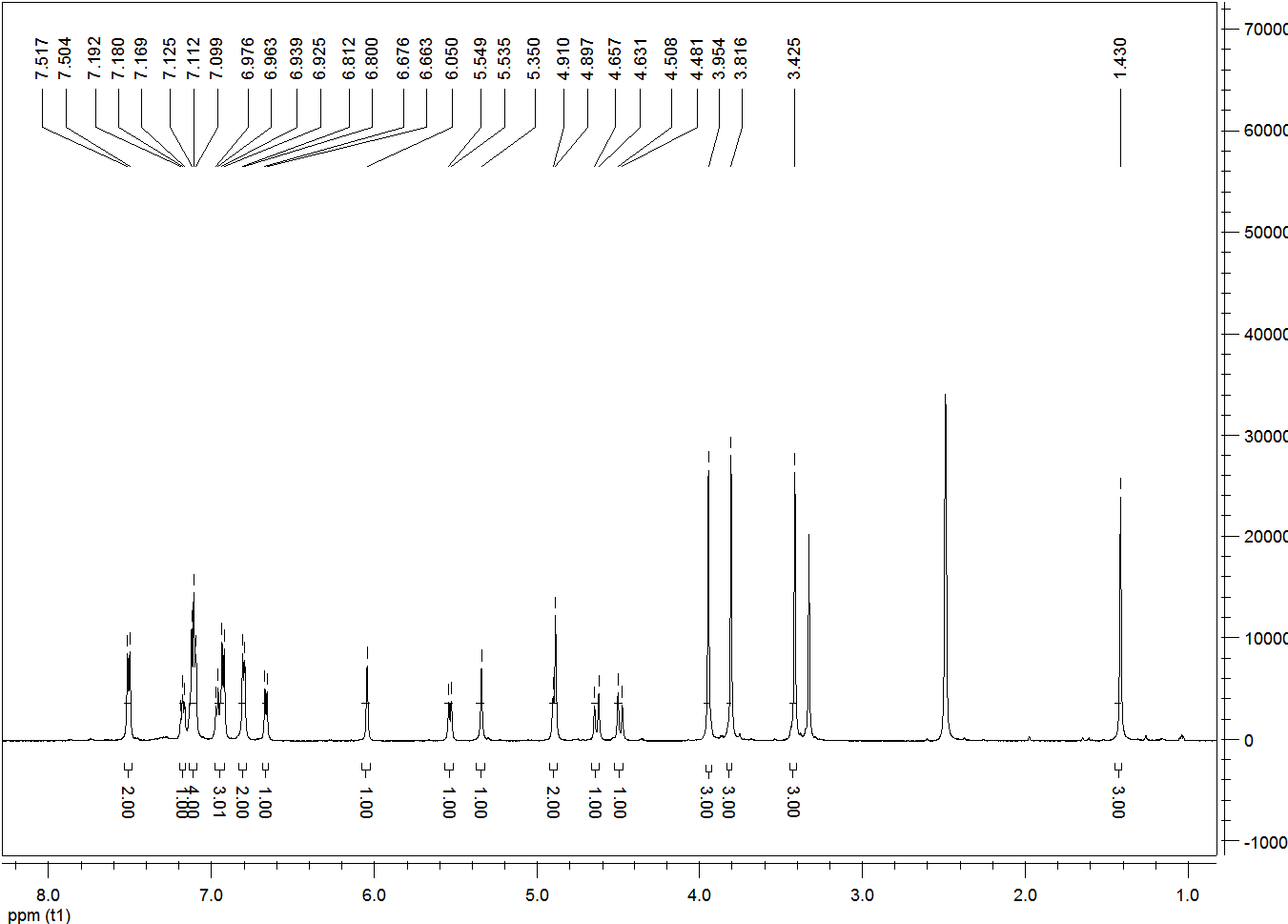


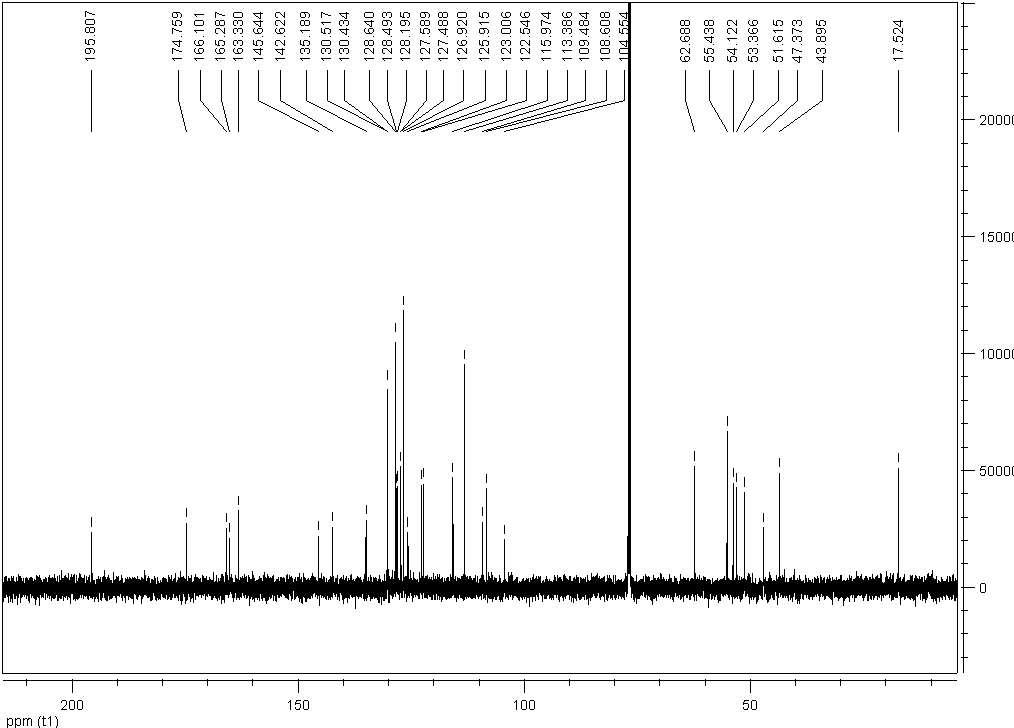


**dimethyl 1-benzyl-5-fluoro-2'-(3-methoxybenzoyl)-7'-methyl-2-oxo-2',9a'-dihydrospiro[indo-**

**line-3,1'-quinolizine]-3',4'-dicarboxylate (1d)**: yellow solid, 58%, m.p. 172~173℃; ^1^H NMR (600 MHz, DMSO-*d_6_*) δ: 7.36 (brs, 1H, ArH), 7.18~7.13 (m, 5H, ArH), 7.05~7.00 (m, 2H, ArH), 6.85 (brs, 3H, ArH), 6.74 (brs, 1H, ArH), 6.11 (s, 1H, CH), 5.59 (brs, 1H,CH), 5.41 (s, 1H, CH), 4.97~4.93 (m, 2H, CH), 4.60 (brs, 1H,CH), 4.48 (brs, 1H,CH), 3.97 (s, 3H, OCH_3_), 3.73 (s, 3H, OCH_3_), 3.45 (s, 3H, OCH_3_), 1.47 (s, 3H, CH_3_); ^13^C NMR (150 MHz, CDCl_3_) δ: 197.1, 174.4, 165.5 (d, *J* = 134.6Hz), 159.5, 145.7, 138.8, 138.7, 134.9, 129.3, 128.8, 128.5, 127.7, 126.9, 122.5, 120.8, 120.3, 115.7, 115.5 (d, *J* = 24.9Hz), 115.1 (d, *J* = 24.3Hz), 111.5, 109.7, 109.1 (d, *J* = 5.7Hz), 104.3, 62.6, 55.4, 54.4, 53.4, 51.7, 47.9, 44.1, 17.5; IR (KBr) υ: 3451, 2948, 2839, 2025, 1742, 1710, 1612, 1582, 1486, 1452, 1433, 1410, 1342, 1294, 1251, 1194, 1176, 1117, 1050, 1009, 983, 946, 896, 868, 841, 813, 788, 752 cm^-1^; MS (*m*/*z*): HRMS (ESI) Calcd. for C_36_H_32_FN_2_O_7_ ([M+H]^+^): 623.2201. Found: 623.2196.

**
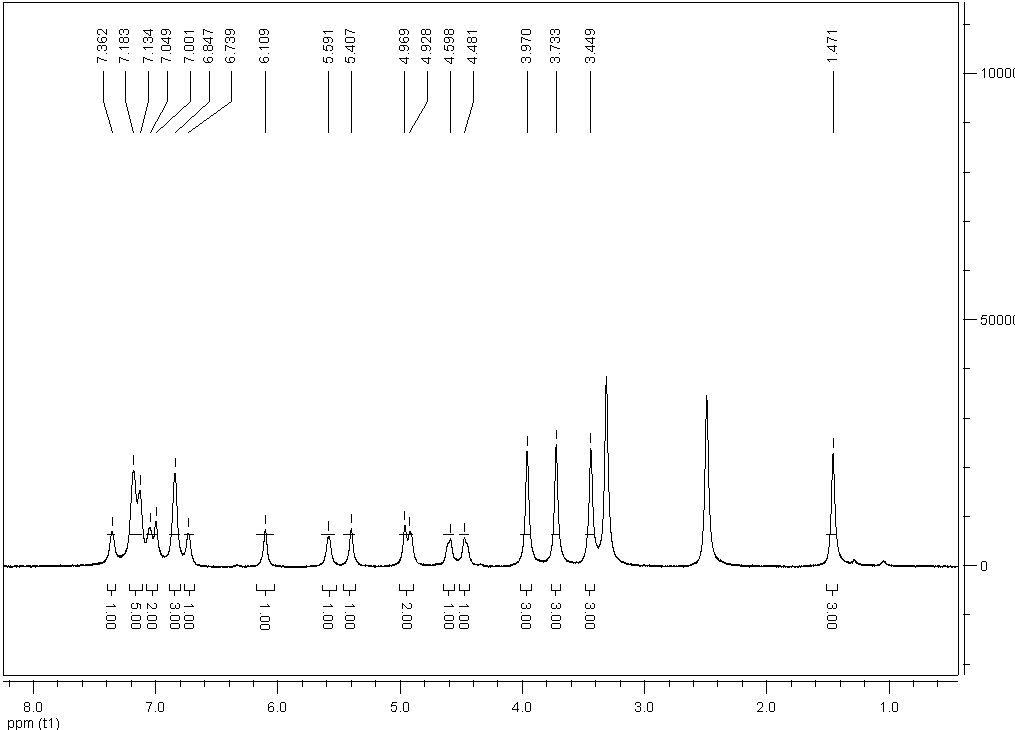
**

**
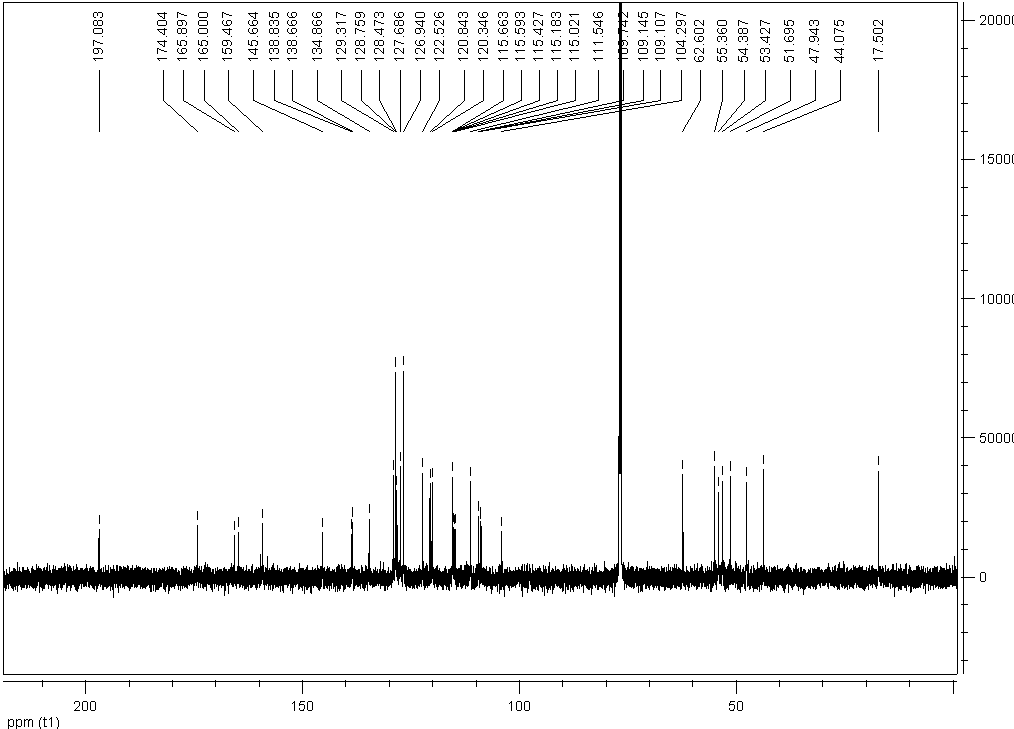
**

**dimethyl 1-benzyl-5-chloro-7'-methyl-2'-(4-methylbenzoyl)-2-oxo-2',9a'-dihydrospiro[indo-**

**line-3,1'-quinolizine]-3',4'-dicarboxylate (1e)**: yellow solid, 62%, m.p. 169~172℃; ^1^H NMR (600 MHz, DMSO-*d_6_*) δ: 7.48 (d, *J* = 7.8Hz, 2H, ArH), 7.24 (d, *J* = 7.8Hz, 3H, ArH), 7.19 (t, *J* = 7.2Hz, 1H, ArH), 7.12~7.09 (m, 3H, ArH), 6.76 (d, *J* = 7.2Hz, 2H, ArH), 6.72 (d, *J* = 8.4Hz, 1H, ArH), 6.11 (s, 1H, CH), 5.59 (d, *J* = 9.6Hz, 1H, CH), 5.42 (s, 1H, CH), 4.96 (s, 1H, CH), 4.91 (d, *J* = 9.6Hz, 1H, CH), 4.64 (d, *J* = 15.6Hz, 1H, CH), 4.45 (d, *J* = 15.6Hz, 1H, CH), 3.97 (s, 3H, OCH_3_), 3.43 (s, 3H, OCH_3_), 2.36 (s, 3H, CH_3_), 1.47 (s, 3H, CH_3_); ^13^C NMR (150 MHz, CDCl_3_) δ: 196.7, 174.2, 165.9, 165.1, 145.6, 143.7, 141.2, 134.8, 134.7, 129.0, 128.7, 128.6, 128.4, 127.8, 127.6, 126.9, 122.6, 115.5, 109.8, 109.6, 104.6, 62.6, 54.3, 53.5, 51.7, 47.7, 43.9, 21.7, 17.5; IR (KBr) υ: 3446, 3040, 2948, 2853, 2025, 1716, 1680, 1611, 1584, 1482, 1434, 1404, 1371, 1295, 1243, 1178, 1115, 1076, 983, 942, 899, 801, 743, 703 cm^-1^; MS (*m*/*z*): HRMS (ESI) Calcd. for C_36_H_32_ClN_2_O_6_ ([M+H]^+^): 623.1957. Found: 623.1958.


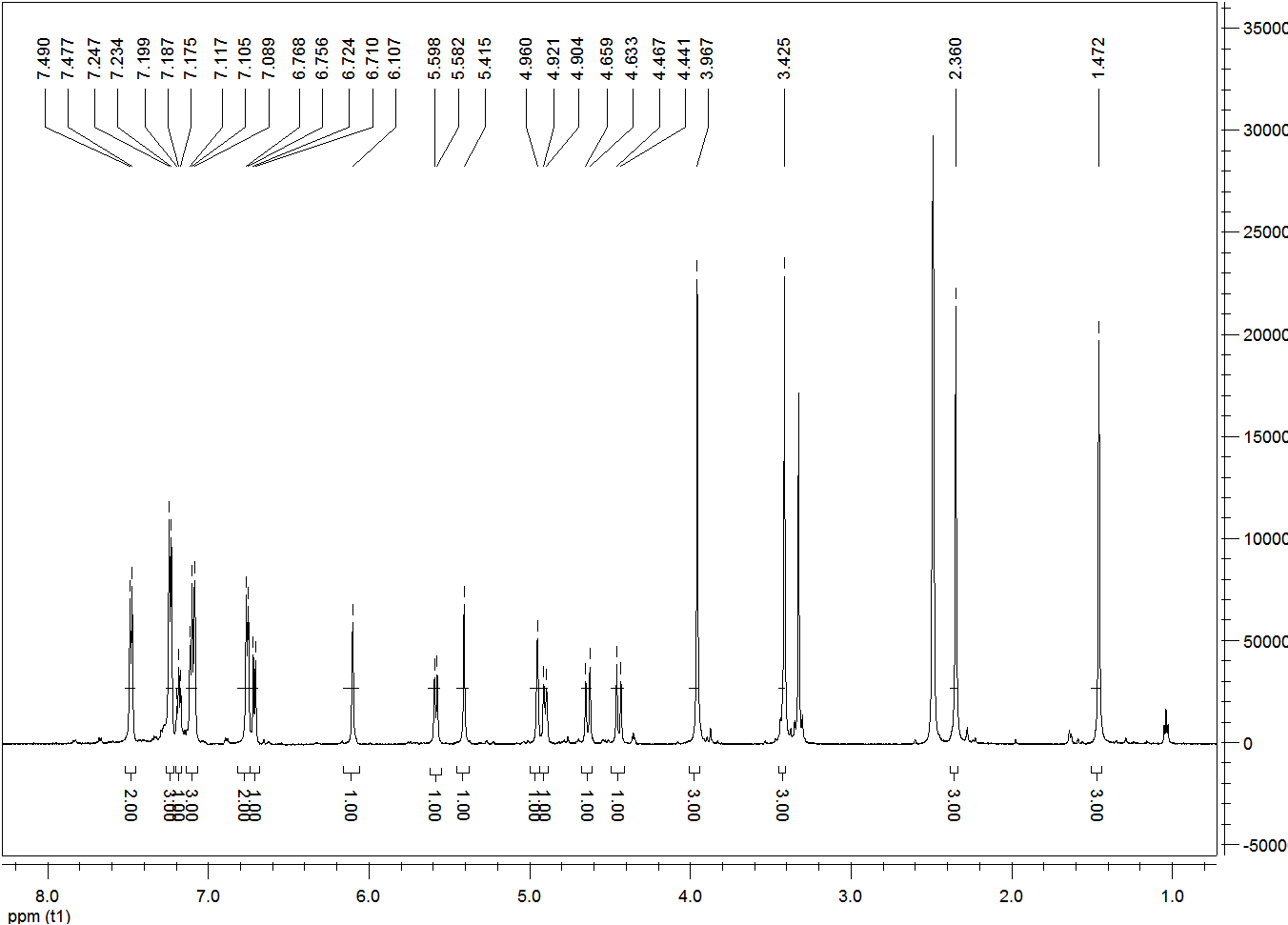


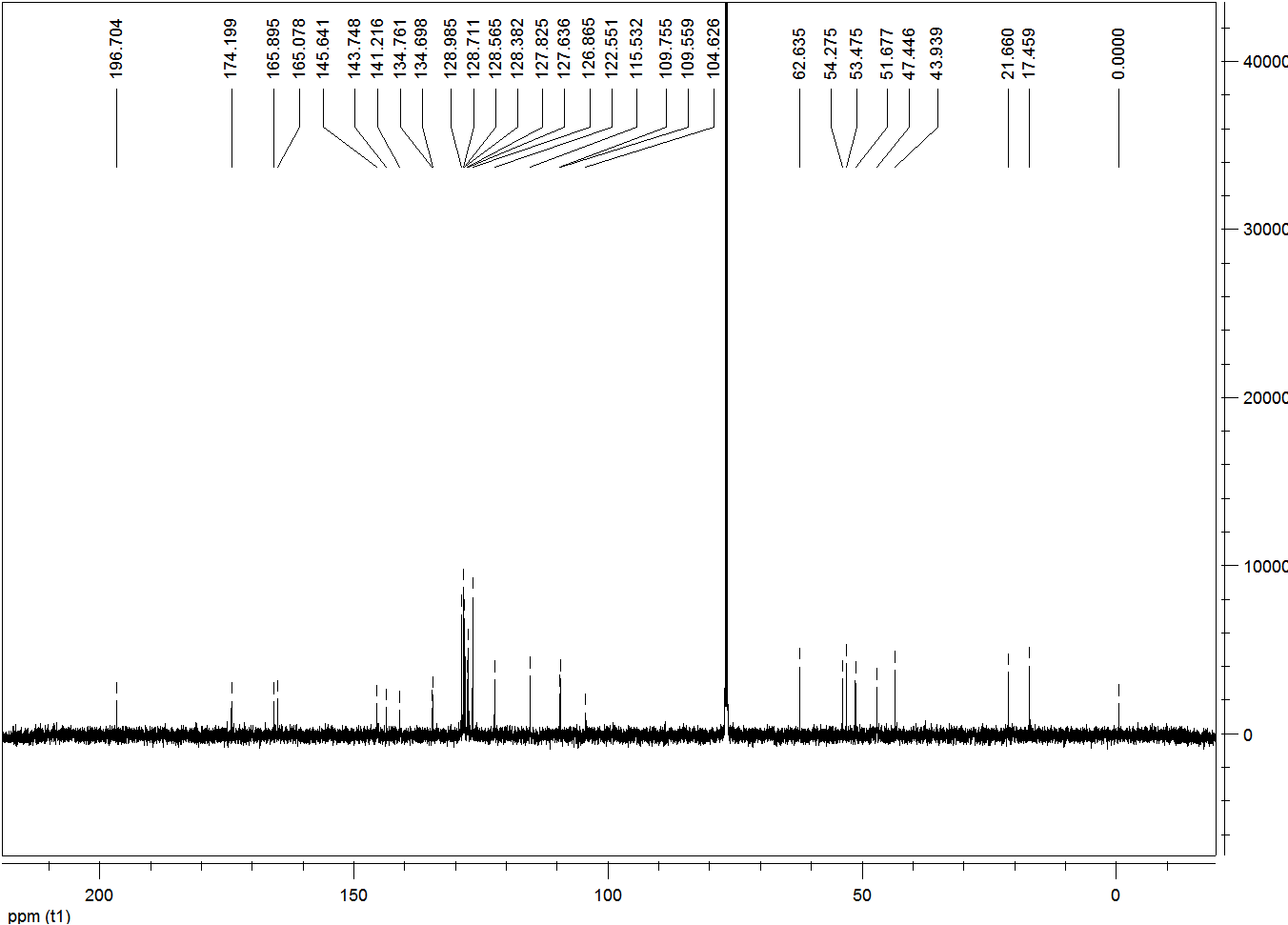


**dimethyl 1-butyl-5-fluoro-2'-(4-methoxybenzoyl)-7'-methyl-2-oxo-2',9a'-dihydrospiro[indo-**

**line-3,1'-quinolizine]-3',4'-dicarboxylate (1f)**: yellow solid, 74%, m.p. 162~163℃; ^1^H NMR (600 MHz, DMSO-*d_6_*) δ: 7.44 (d, *J* = 9.0Hz, 2H, ArH), 7.09 (td, *J_1_* = 9.0Hz, *J_2_* = 2.4Hz, 1H, ArH), 6.90~6.89 (m, 3H, ArH), 6.82 (dd, *J_1_* = 8.7Hz, *J_2_* = 2.4Hz, 1H, ArH), 6.09 (s, 1H, CH), 5.62 (d, *J* = 9.6Hz, 1H, CH), 5.28 (s, 1H, CH), 4.92~4.90 (m, 1H, CH), 4.88 (brs, 1H, CH), 3.96 (s, 3H, OCH_3_), 3.79 (s, 3H, OCH_3_), 3.42 (s, 3H, OCH_3_), 3.40~3.36 (m, 1H, CH), 3.25~3.21 (m, 1H, CH), 1.48 (s, 3H, CH_3_), 0.99~0.91 (m, 3H, CH), 0.79~0.73 (m, 1H, CH), 0.69 (t, *J* = 7.2Hz, 3H, CH_3_); ^13^C NMR (150 MHz, DMSO-*d_6_*) δ: 195.8, 174.0, 165.9, 165.1, 163.3, 158.9 (d, *J* = 119.5Hz), 145.5, 138.9, 130.5, 130.4, 128.2, 127.9, 127.8, 122.5, 115.8, 115.6 (d, *J* = 25.2Hz), 114.9 (d, *J* = 23.9Hz), 113.3, 109.6, 108.1 (d, *J* = 8.3Hz), 104.3, 62.2, 55.3, 54.1, 53.3, 51.6, 47.4, 40.0, 29.1, 20.0, 17.5, 13.6; IR (KBr) υ: 3453, 2955, 2924, 2867, 2025, 1746, 1708, 1680, 1600, 1582, 1490, 1455, 1403, 1368, 1329, 1305, 1262, 1237, 1172, 1107, 1076, 1027, 1000, 981, 952, 895, 849, 815, 758 cm^-1^; MS (*m*/*z*): HRMS (ESI) Calcd. for C_33_H_34_FN_2_O_7_ ([M+H]^+^): 589.2358. Found: 589.2353.


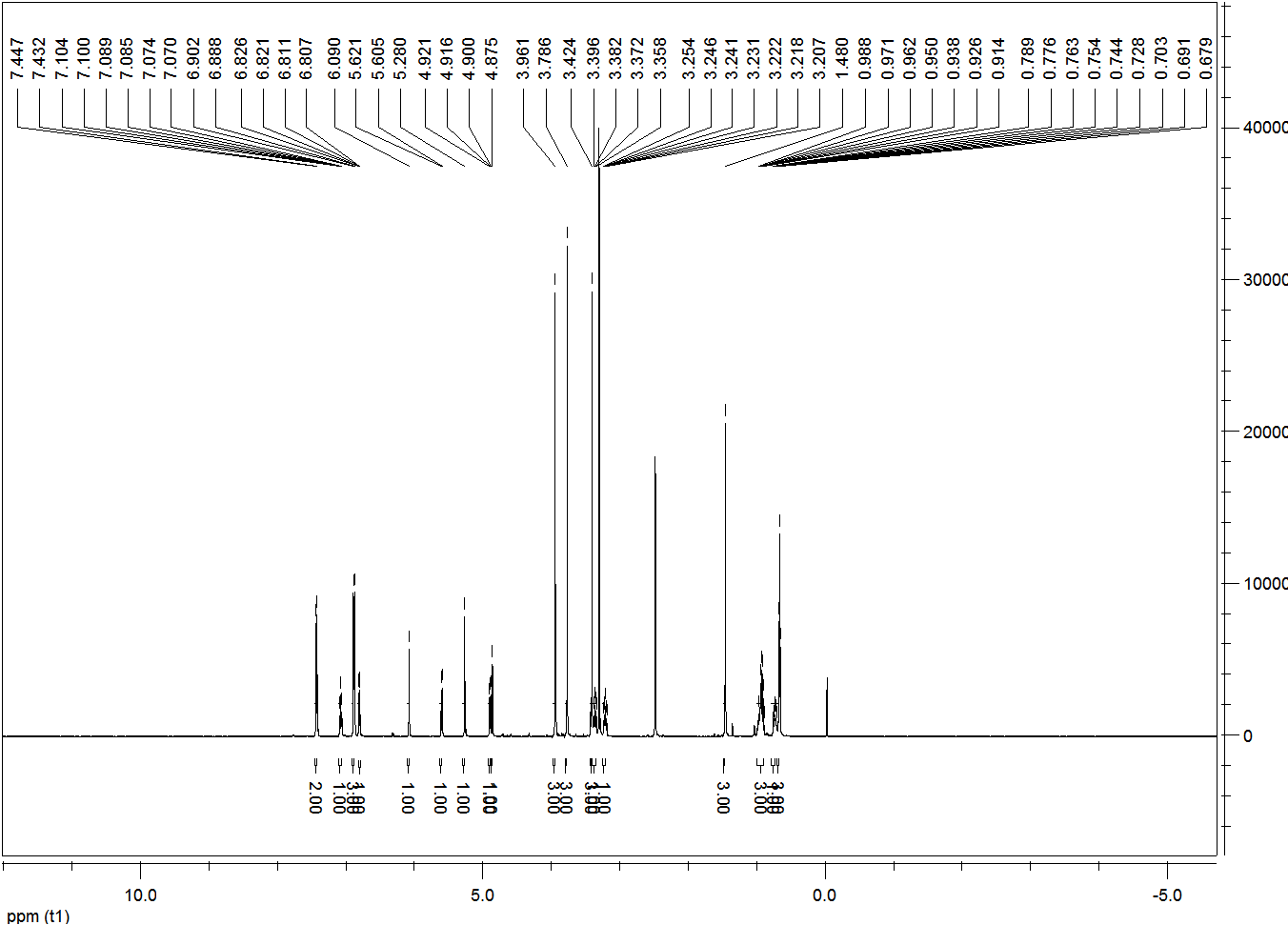


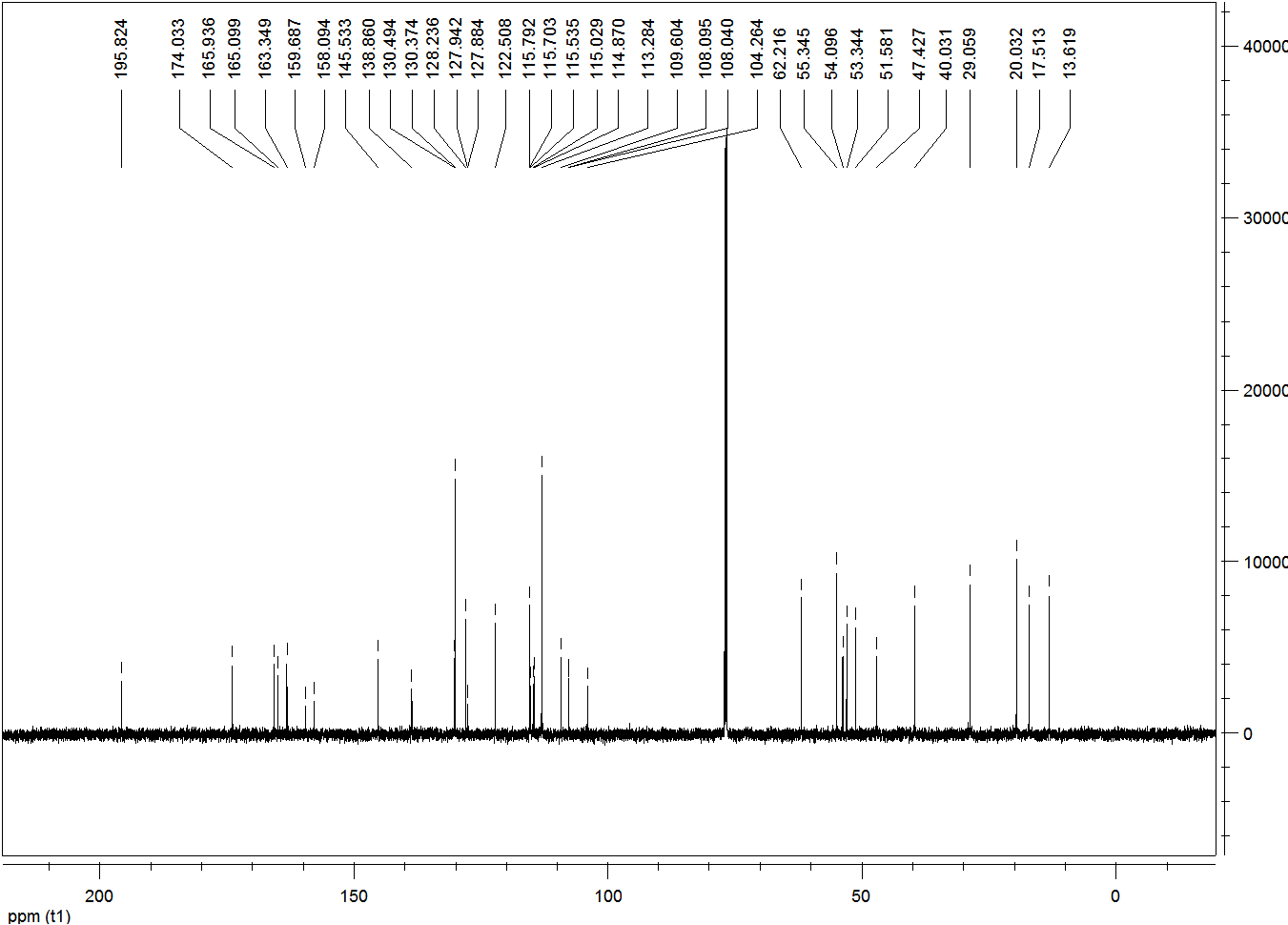


**dimethyl 1-butyl-5-fluoro-2'-(4-methoxybenzoyl)-8'-methyl-2-oxo-2',9a'-dihydrospiro[indo-**

**line-3,1'-quinolizine]-3',4'-dicarboxylate (1g)**: yellow solid, 77%, m.p. 164~166℃; ^1^H NMR (600 MHz, DMSO-*d_6_*) δ: 7.42 (d, *J* = 7.2Hz, 2H, ArH), 7.08 (brs, 1H, ArH), 6.89 (d, *J* = 7.2Hz, 3H, ArH), 6.79 (d, *J* = 7.8Hz, 1H, ArH), 6.33 (d, *J* = 7.2Hz, 1H, CH), 5.26 (s, 1H, CH), 4.88 (s, 1H, CH), 4.73 (d, *J* = 7.2Hz, 1H, CH), 4.61 (s, 1H, CH), 3.95 (s, 3H, OCH_3_), 3.79 (s, 3H, OCH_3_), 3.44 (s, 3H, OCH_3_), 3.36 (brs, 1H, CH), 3.29 (brs, 1H, CH), 1.38 (s, 3H, CH_3_), 1.06 (brs, 1H, CH), 0.98~0.97 (m, 2H, CH), 0.86 (brs, 1H, CH), 0.71 (brs, 3H, CH_3_); ^13^C NMR (150 MHz, CDCl_3_) δ: 195.9, 174.1, 165.8, 164.9, 163.3, 145.4, 139.0, 132.6, 130.5, 130.4, 128.0, 127.9, 126.6, 115.5 (d, *J* = 25.2Hz), 114.9 (d, *J* = 24.3Hz), 113.6, 113.5, 113.3, 112.1, 110.2, 108.0 (d, *J* = 8.3Hz), 105.8, 104.7, 63.1, 56.9, 55.4, 54.4, 53.4, 51.7, 47.5, 40.0, 29.4, 29.1, 20.8, 20.5, 20.1, 20.0, 13.7; IR (KBr) υ: 3449, 2953, 2927, 2867, 2026, 1734, 1700, 1671, 1595, 1490, 1437, 1377, 1313, 1278, 1245, 1200, 1174, 1141, 1120, 1023, 952, 885, 853, 816, 790, 755, 729 cm^-1^; MS (*m*/*z*): HRMS (ESI) Calcd. for C_33_H_34_FN_2_O_7_ ([M+H]^+^): 589.2358. Found: 589.2366.


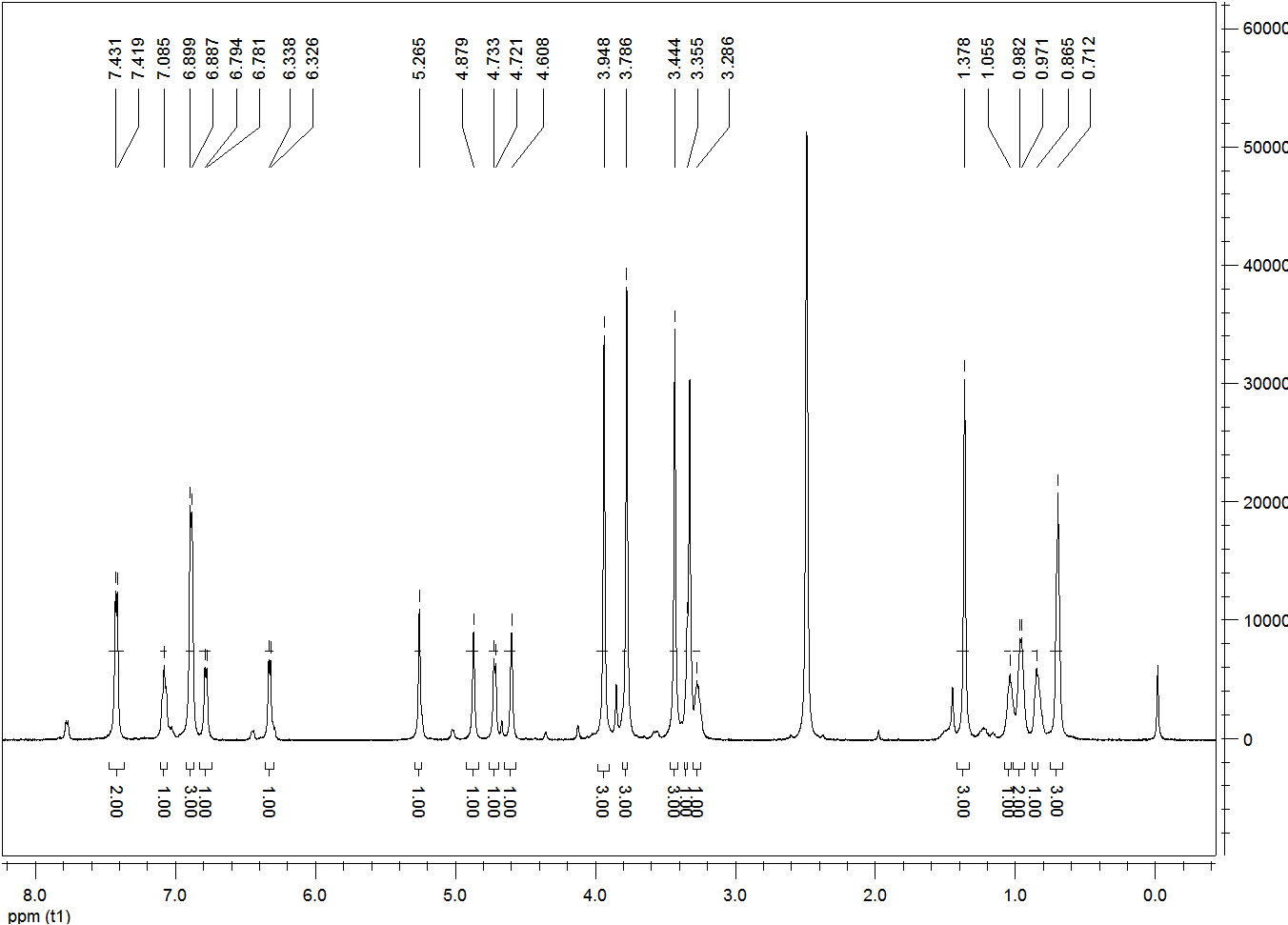


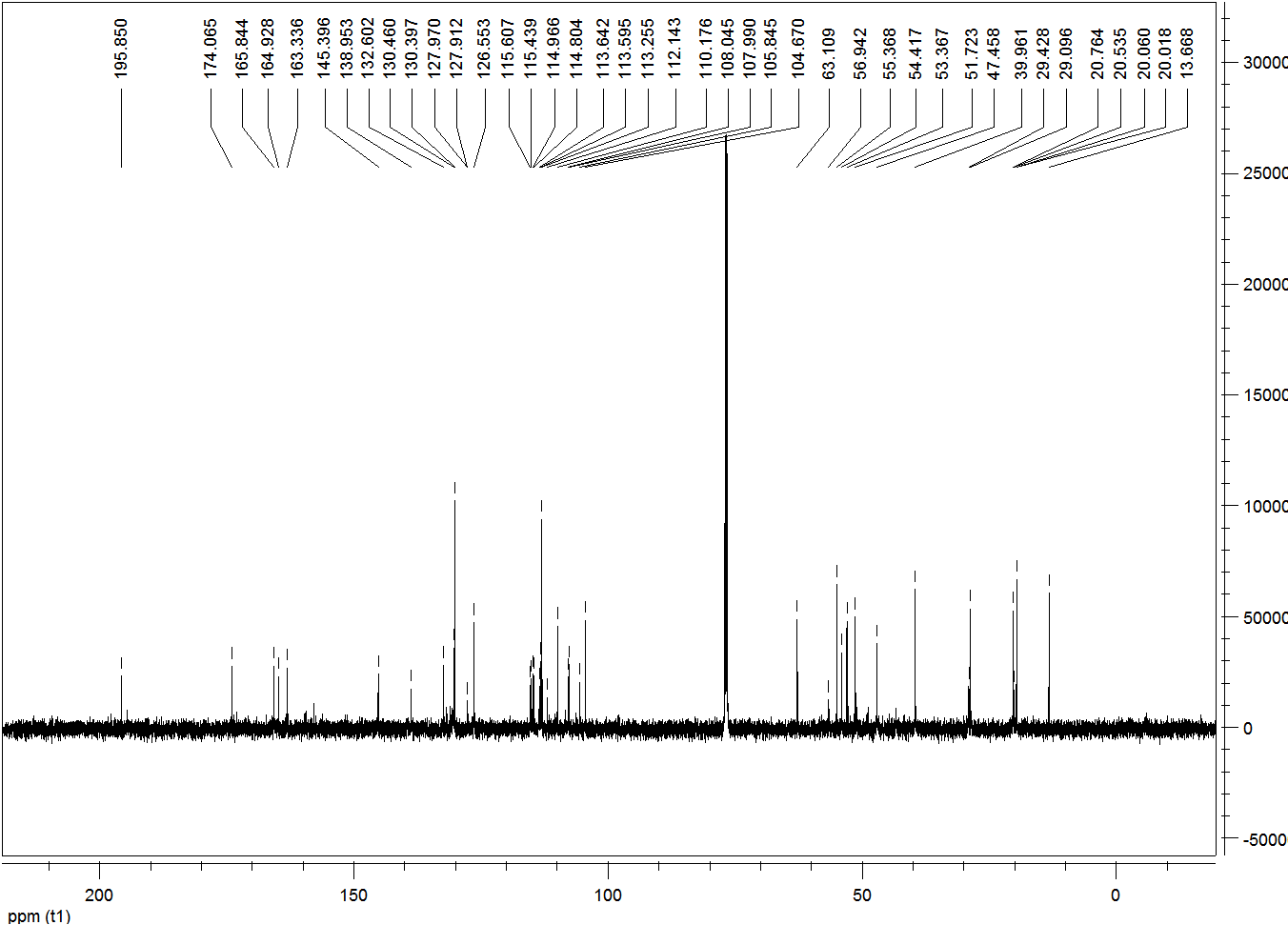


**dimethyl 1-butyl-5-chloro-2'-(4-methoxybenzoyl)-8'-methyl-2-oxo-2',9a'-dihydrospiro[indo-**

**line-3,1'-quinolizine]-3',4'-dicarboxylate (1h)**: yellow solid, 75%, m.p. 168~171℃; ^1^H NMR (600 MHz, DMSO-*d_6_*) δ: 7.43 (d, *J* = 7.8Hz, 2H, ArH), 7.29 (d, *J* = 7.8Hz, 1H, ArH), 7.02 (s, 1H, ArH), 6.90 (t, *J* = 8.4Hz, 3H, ArH), 6.33 (d, *J* = 7.8Hz, 1H, CH), 5.27 (s, 1H, CH), 4.89 (s, 1H, CH), 4.73 (d, *J* = 7.2Hz, 1H, CH), 4.60 (s, 1H, CH), 3.95 (s, 3H, OCH_3_), 3.79 (s, 3H, OCH_3_), 3.44 (s, 3H, OCH_3_), 3.37 (brs, 1H, CH), 3.29~3.26 (m, 1H, CH), 1.38 (s, 3H, CH_3_), 1.05 (brs, 1H, CH), 0.99~0.95 (m, 2H, CH), 0.86 (brs, 1H, CH), 0.71 (t, *J* = 7.2Hz, 3H, CH_3_); ^13^C NMR (150 MHz, CDCl_3_) δ: 195.7, 173.9, 165.8, 164.9, 163.3, 145.4, 141.6, 132.7, 130.4, 128.4, 127.9, 127.8, 127.7, 126.5, 113.3, 110.1, 108.5, 106.0, 104.7, 63.2, 55.4, 54.4, 53.4, 51.7, 47.5, 39.9, 29.1, 20.8, 20.0, 13.7; IR (KBr) υ: 3456, 3071, 2952, 2869, 2587, 2027, 1742, 1710, 1672, 1605, 1579, 1511, 1483, 1432, 1381, 1324, 1243, 1173, 1127, 1045, 1023, 982, 955, 913, 869, 836, 810, 730 cm^-1^; MS (*m*/*z*): HRMS (ESI) Calcd. for C_33_H_34_ClN_2_O_7_ ([M+H]^+^): 605.2062. Found: 605.2067.


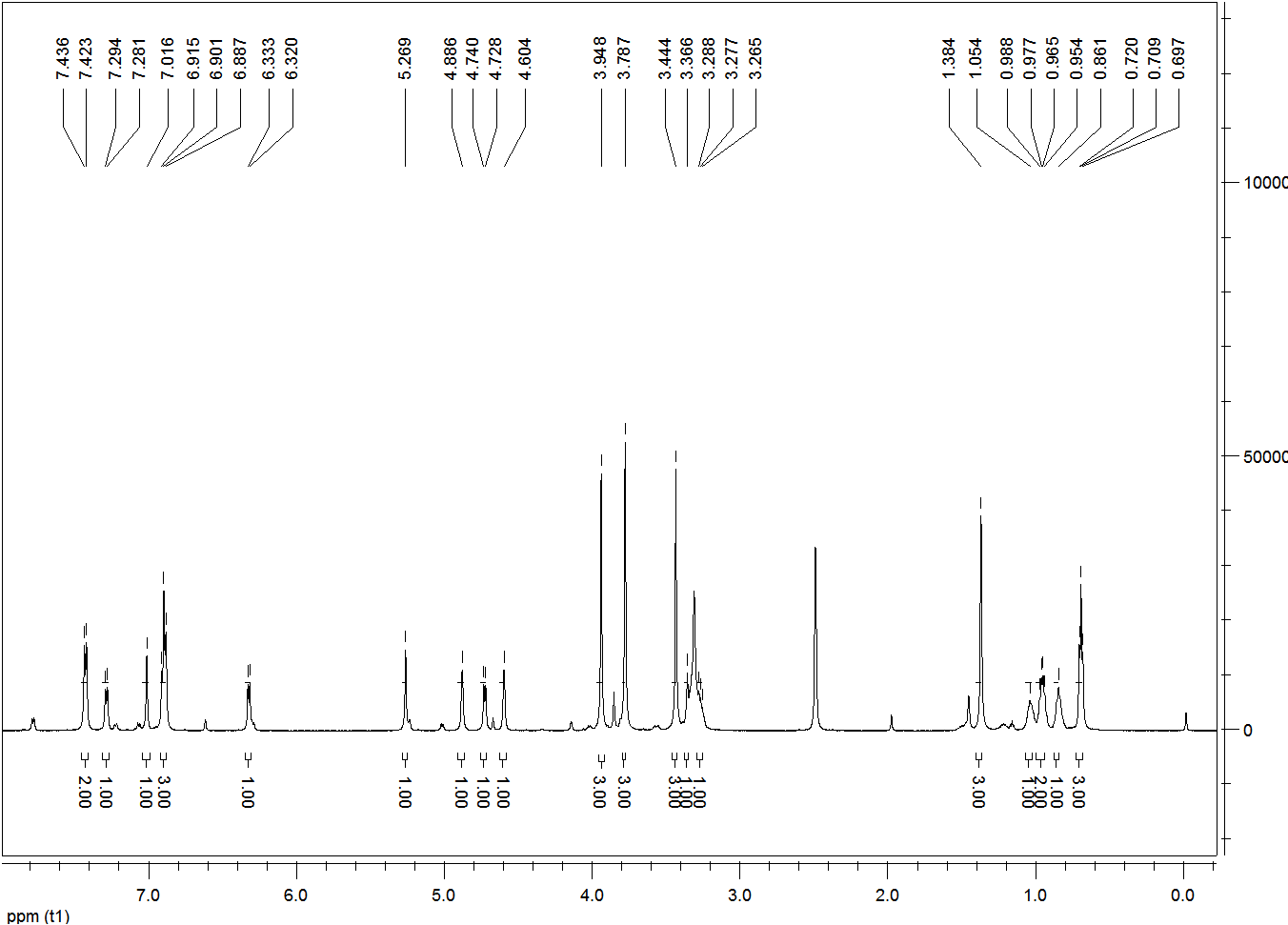


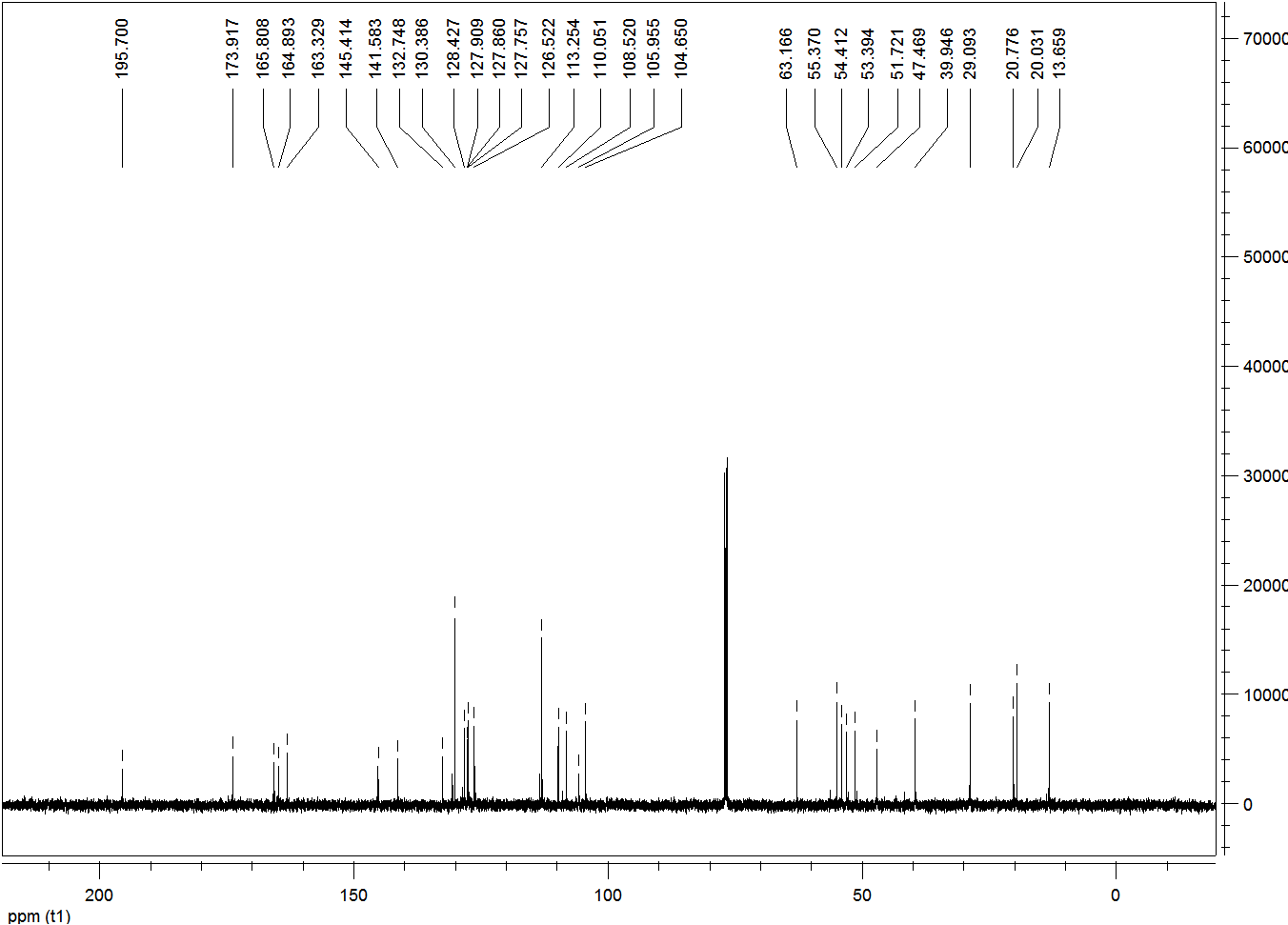


**dimethyl 1-benzyl-5-chloro-8'-methoxy-2'-(4-methoxybenzoyl)-2-oxo-2',9a'-dihydrospiro-**

**[indoline-3,1'-quinolizine]-3',4'-dicarboxylate (1i)**: yellow solid, 89%, m.p. 150.0~150.3℃; ^1^H NMR (600 MHz, DMSO-*d_6_*) δ: 7.54 (d, *J* = 7.2Hz, 2H, ArH), 7.25 (d, *J* = 6.6Hz, 1H, ArH), 7.18 (d, *J* = 6.6Hz, 1H, ArH), 7.13 (brs, 2H, ArH), 7.01 (s, 1H, ArH), 6.97~6.95 (m, 4H, ArH), 6.82 (d, *J* = 7.8Hz, 1H, ArH), 6.43 (d, *J* = 7.8Hz, 1H, CH), 5.36 (s, 1H, CH), 5.05 (s, 1H, CH), 4.66 (d, *J* = 7.2Hz, 1H, CH), 4.60~4.54 (m, 2H, CH_2_), 3.95 (s, 3H, OCH_3_), 3.82 (s, 3H, OCH_3_), 3.77 (brs, 1H, CH), 3.47 (s, 3H, OCH_3_), 2.93 (s, 3H, OCH_3_); ^13^C NMR (150 MHz, CDCl_3_) δ: 195.5, 174.5, 165.7, 164.8, 163.5, 153.7, 145.2, 141.4, 135.0, 130.7, 130.6, 130.2, 128.8, 128.6, 128.4, 127.8, 127.7, 127.5, 127.1, 113.5, 113.4, 109.1, 100.2, 83.0, 64.0, 58.4, 55.5, 55.3, 54.1, 53.5, 53.4, 51.9, 47.3, 43.9, 18.4, 15.3; IR (KBr) υ: 3457, 2954, 1745, 1707, 1671, 1628, 1600, 1511, 1484, 1455, 1435, 1377, 1339, 1251, 1226, 1178, 1136, 979, 944, 902, 868, 845, 808, 757cm^-1^; MS (*m*/*z*): HRMS (ESI) Calcd. for C_36_H_32_Cl_2_N_2_O_8_ ([M+H]^+^): 655.1842. Found: 655.1841.


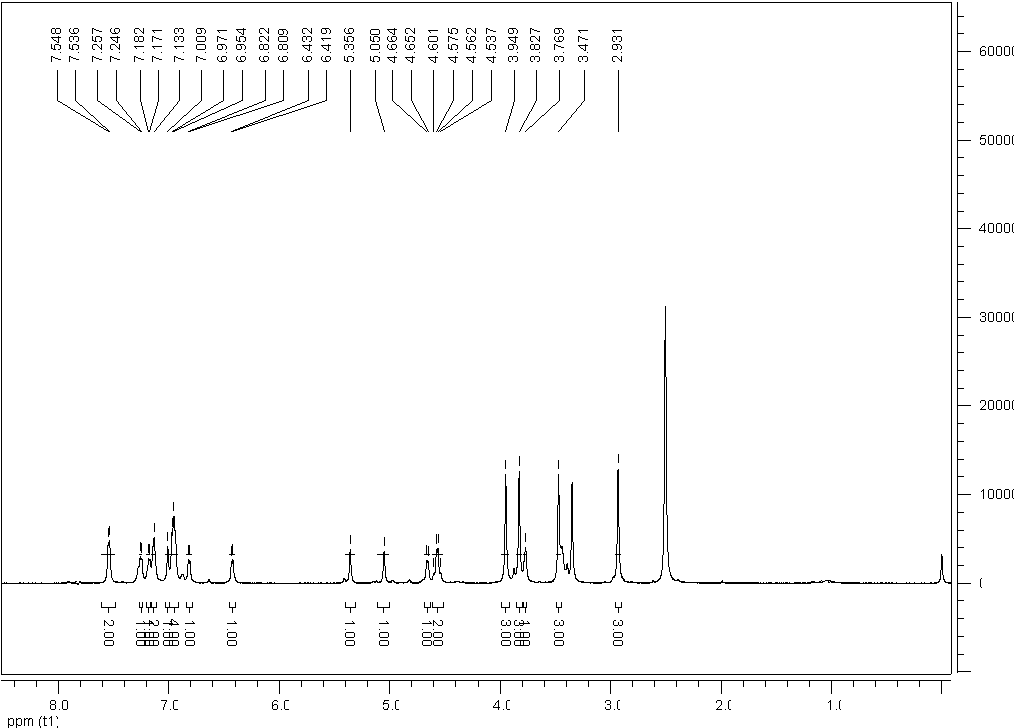


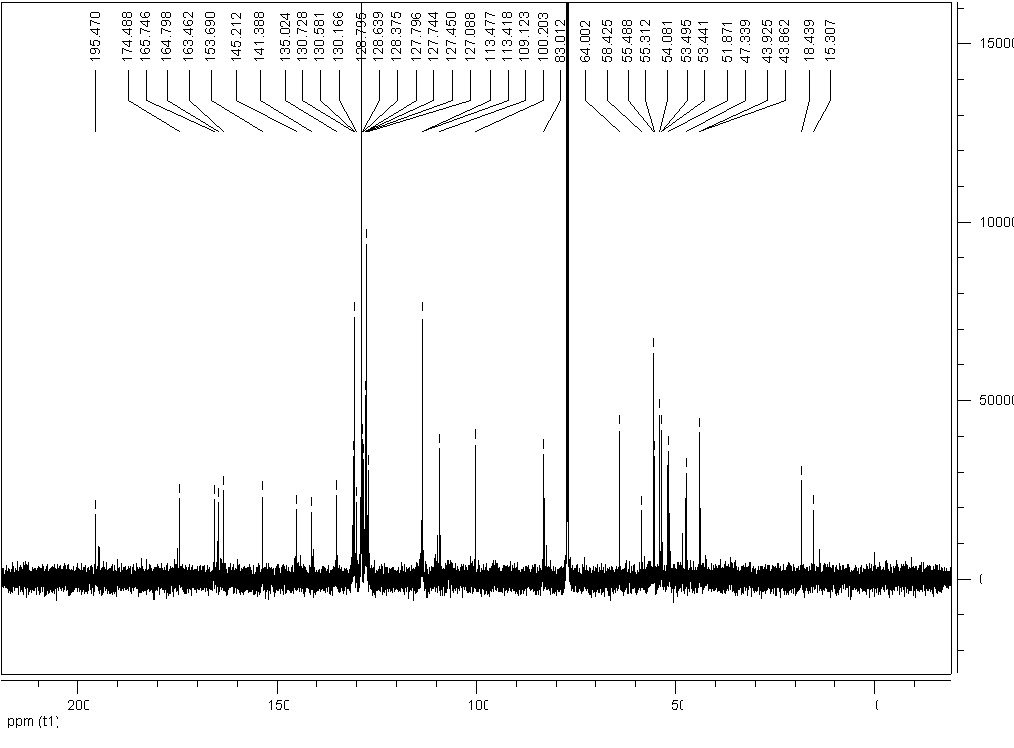


**dimethyl 1-benzyl-5-chloro-8'-methoxy-2'-(4-methylbenzoyl)-2-oxo-2',9a'-dihydrospiro-**

**[indoline-3,1'-quinolizine]-3',4'-dicarboxylate (1j)**: yellow solid, 84%, m.p. 162.3~163.1℃; ^1^H NMR (600 MHz, DMSO-*d_6_*) δ: 7.42 (d, *J* = 7.8Hz, 2H, ArH), 7.27~7.24 (m, 3H, ArH), 7.18 (d, *J* = 7.2Hz, 1H, ArH), 7.14 (t, *J* = 7.2Hz, 2H, ArH), 7.00 (s, 1H, ArH), 6.96 (d, *J* = 7.8Hz, 2H, ArH), 6.82 (d, *J* = 8.4Hz, 1H, ArH), 6.43 (d, *J* = 8.4Hz, 1H, CH), 5.37 (s, 1H, CH), 5.06 (s, 1H, CH), 4.66 (d, *J* = 7.2Hz, 1H, CH), 4.49 (brs, 2H, CH_2_), 3.95 (s, 3H, OCH_3_), 3.75 (brs, 1H, CH), 3.48 (s, 3H, OCH_3_), 2.91 (s, 3H, OCH_3_), 2.36 (s, 3H, CH_3_); ^13^C NMR (150 MHz, CDCl_3_) δ: 196.8, 174.4, 173.4, 165.7, 164.8, 153.7, 145.2, 143.7, 141.4, 135.1, 135.0, 134.8, 129.2, 128.9, 128.8, 128.7, 128.5, 128.3, 127.9, 127.8, 127.7, 127.5, 127.3, 126.9, 100.1, 83.0, 63.9, 55.1, 54.0, 53.5, 51.9, 47.8, 43.9, 21.7, 21.6; IR (KBr) υ: 3455, 2945, 1744, 1716, 1672, 1626, 1600, 1480, 1456, 1432, 1388, 1370, 1317, 1246, 1231, 1175, 1135, 1043, 977, 947, 918, 869, 838, 814, 784cm^-1^; MS (*m*/*z*): HRMS (ESI) Calcd. for C_36_H_32_ClN_2_O_7_ ([M+H]^+^): 639.1893. Found: 639.1898.


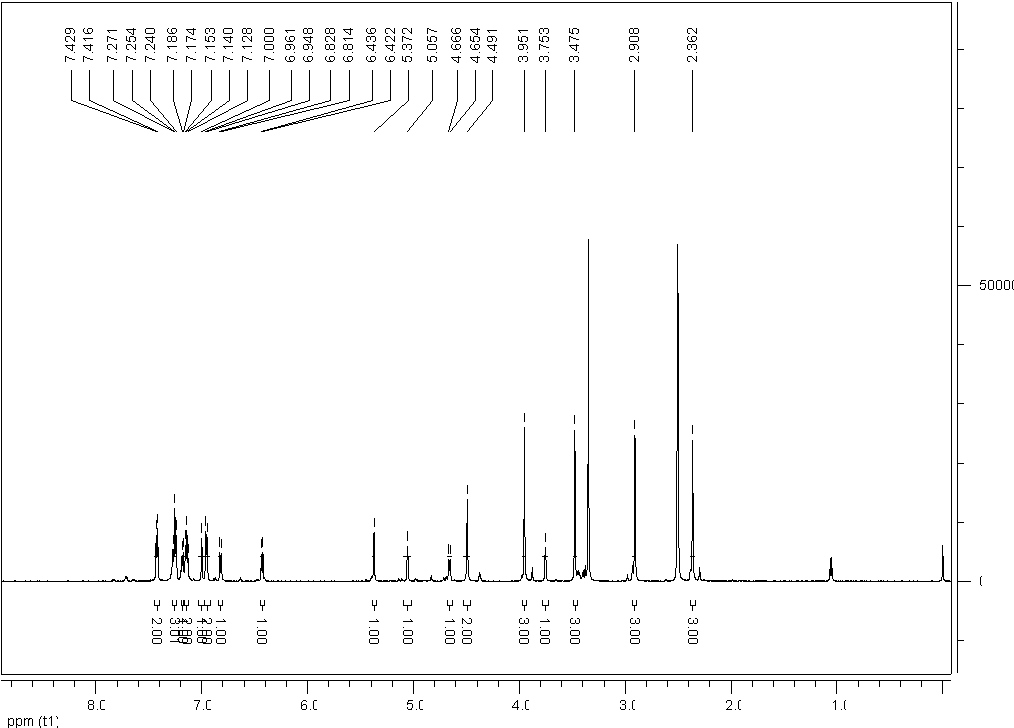


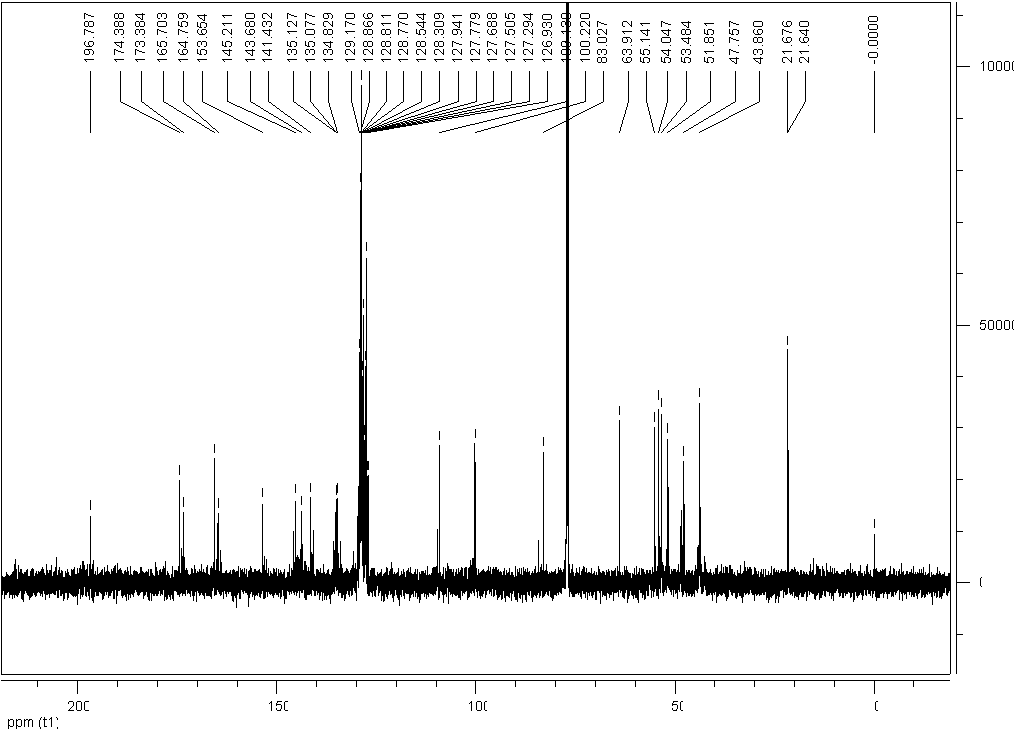


**dimethyl 1-benzyl-5-fluoro-8'-methoxy-2'-(4-methoxybenzoyl)-2-oxo-2',9a'-dihydrospiro-**

**[indoline-3,1'-quinolizine]-3',4'-dicarboxylate (1k)**: yellow solid, 91%, m.p. 147.1~148.0℃; ^1^H NMR (600 MHz, DMSO-*d_6_*) δ: 7.54 (d, *J* = 8.4Hz, 2H, ArH), 7.18 (t, *J* = 7.2Hz, 1H, ArH), 7.13 (d, *J* = 7.2Hz, 2H, ArH), 7.04 (t, *J* = 8.4Hz, 1H, ArH), 6.96 (t, *J* = 7.8Hz, 4H, ArH), 6.79~6.78 (m, 2H, ArH), 6.43 (d, *J* = 7.8Hz, 1H, CH), 5.35 (s, 1H, CH), 5.04 (d, *J* = 3.0Hz, 1H, CH), 4.65 (d, *J* = 6.6Hz, 1H, CH), 4.56 (brs, 2H, CH_2_), 3.95 (s, 3H, OCH_3_), 3.83 (s, 3H, OCH_3_), 3.78 (brs, 1H, CH), 3.47 (s, 3H, OCH_3_), 2.93 (s, 3H, OCH_3_); ^13^C NMR (150 MHz, DMSO-*d_6_*) δ: 195.3, 173.6, 165.0, 164.0, 163.3, 157.9 (d, *J* = 236.9Hz), 153.0, 144.8, 139.1, 135.6, 130.2, 129.6, 129.3, 128.5, 127.4, 127.3, 127.2, 115.1 (d, *J* = 23.1Hz), 114.3 (d, *J* = 28.2Hz), 113.6, 109.4 (d, *J* = 6.2Hz), 106.1, 99.7, 82.8, 63.3, 56.0, 55.5, 54.5, 53.8, 53.4, 51.6, 46.4, 43.0, 18.5; IR (KBr) υ: 3450, 1737, 1641, 1488, 1422, 1369, 1285, 1232, 1187, 1111, 952, 865, 816, 774cm^-1^; MS (*m*/*z*): HRMS (ESI) Calcd. for C_36_H_32_FN_2_O_8_ ([M+H]^+^): 639.2137. Found: 639.2142.


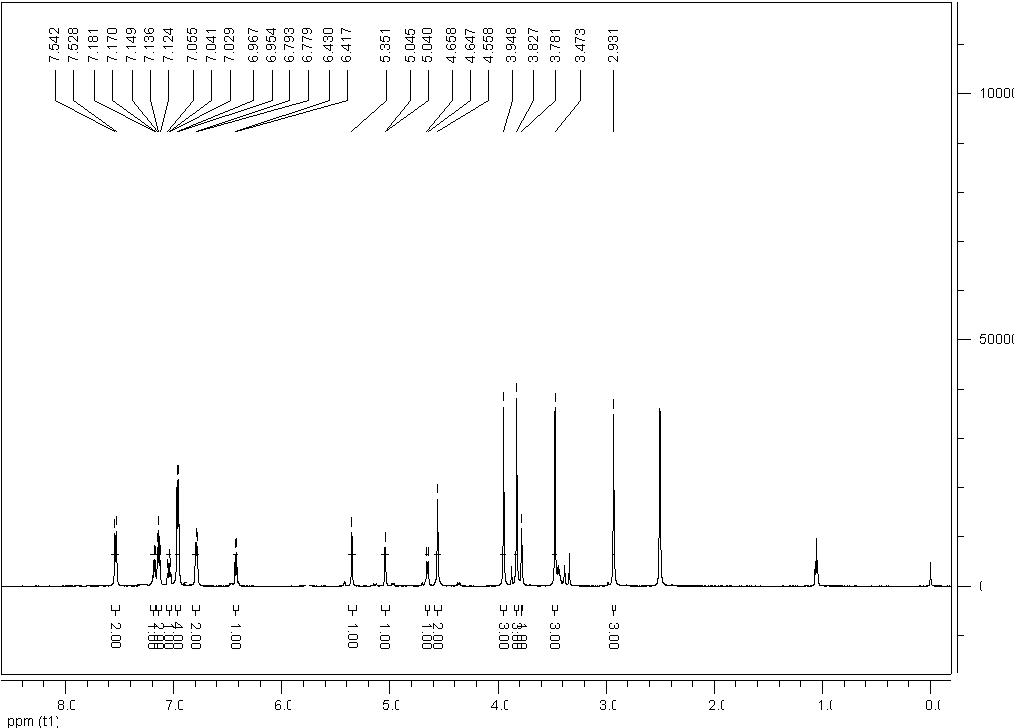


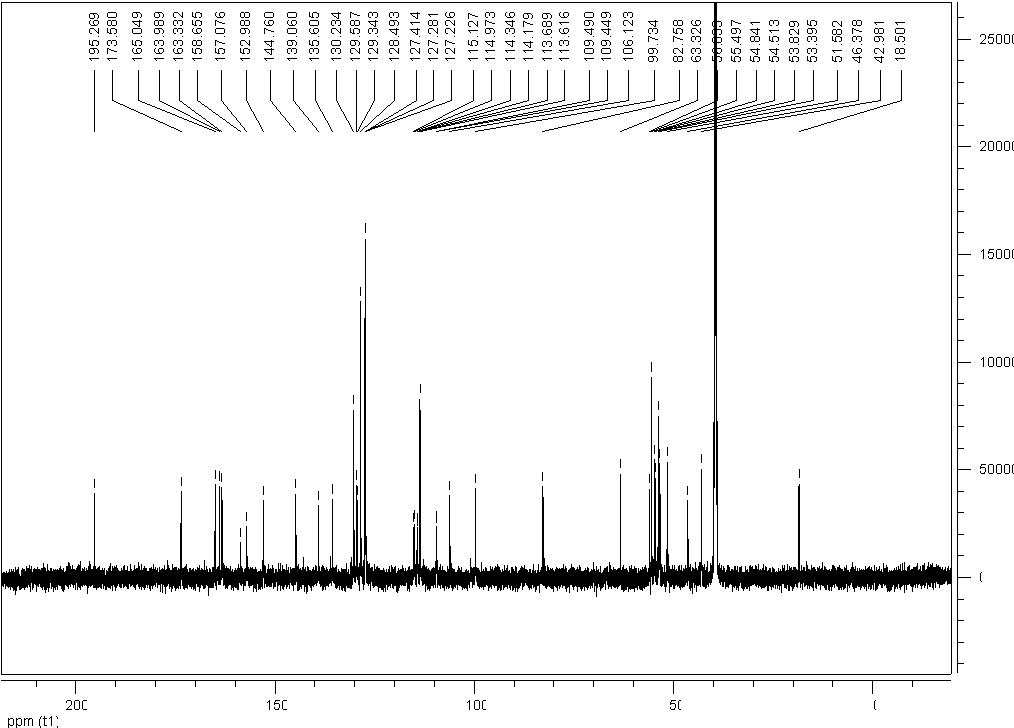


**dimethyl 1-benzyl-5-fluoro-8'-methoxy-2'-(4-methylbenzoyl)-2-oxo-2',9a'-dihydrospiro[indo-**

**line-3,1'-quinolizine]-3',4'-dicarboxylate (1l)**: yellow solid, 87%, m.p. 158.5~159.0℃; ^1^H NMR (600 MHz, DMSO-*d_6_*) δ: 7.42 (d, *J* = 7.8Hz, 2H, ArH), 7.24 (t, *J* = 7.8Hz, 2H, ArH), 7.18 (t, *J* = 7.2Hz, 1H, ArH), 7.14 (t, *J* = 7.2Hz, 2H, ArH), 7.06 (t, *J* = 8.4Hz, 1H, ArH), 6.97 (t, *J* = 7.2Hz, 2H, ArH), 6.81~6.77 (m, 2H, ArH), 6.43 (d, *J* = 8.4Hz, 1H, CH), 5.37 (s, 1H, CH), 5.05 (d, *J* = 8.4Hz, 1H, CH), 4.66 (d, *J* = 7.8Hz, 1H, CH), 4.48 (brs, 2H, CH_2_), 3.95 (s, 3H, OCH_3_), 3.76 (brs, 1H, CH), 3.48 (s, 3H, OCH_3_), 2.91 (s, 3H, OCH_3_), 2.36 (s, 3H, CH_3_); ^13^C NMR (150 MHz, CDCl_3_) δ: 196.9, 174.6, 165.7, 164.8, 159.0 (d, *J* = 240Hz), 153.6, 145.2, 143.7, 138.8, 135.2 134.8, 128.9, 128.8, 128.3, 127.7, 115.4 (d, *J* = 25.4Hz), 115.1 (d, *J* = 23.4Hz), 108.7 (d, *J* = 7.5Hz), 106.5, 100.2, 83.1, 63.9, 55.2, 54.1, 53.5, 51.8, 47.7, 43.9, 21.6; IR (KBr) υ: 3454, 2946, 1745, 1715, 1675, 1625, 1597, 1487, 1454, 1434, 1387, 1317, 1297, 1243, 1227, 1179, 1153, 1128, 1045, 981, 947, 892, 868, 843, 809, 767cm^-1^; MS (*m*/*z*): HRMS (ESI) Calcd. for C_36_H_32_FN_2_O_7_ ([M+H]^+^): 623.2188. Found: 623.2190.


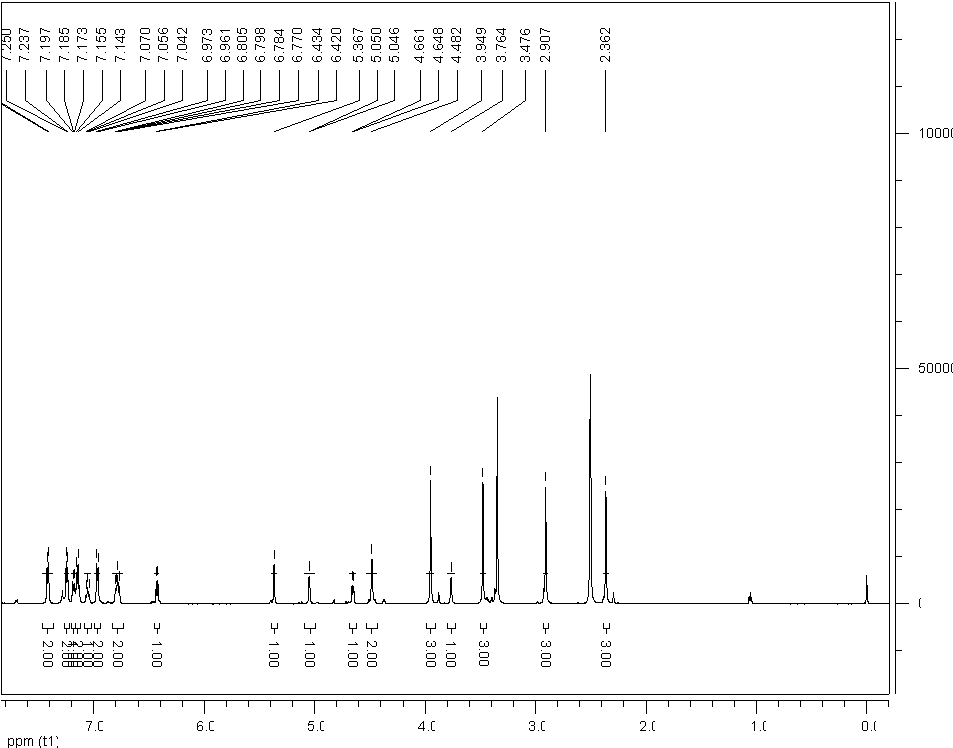


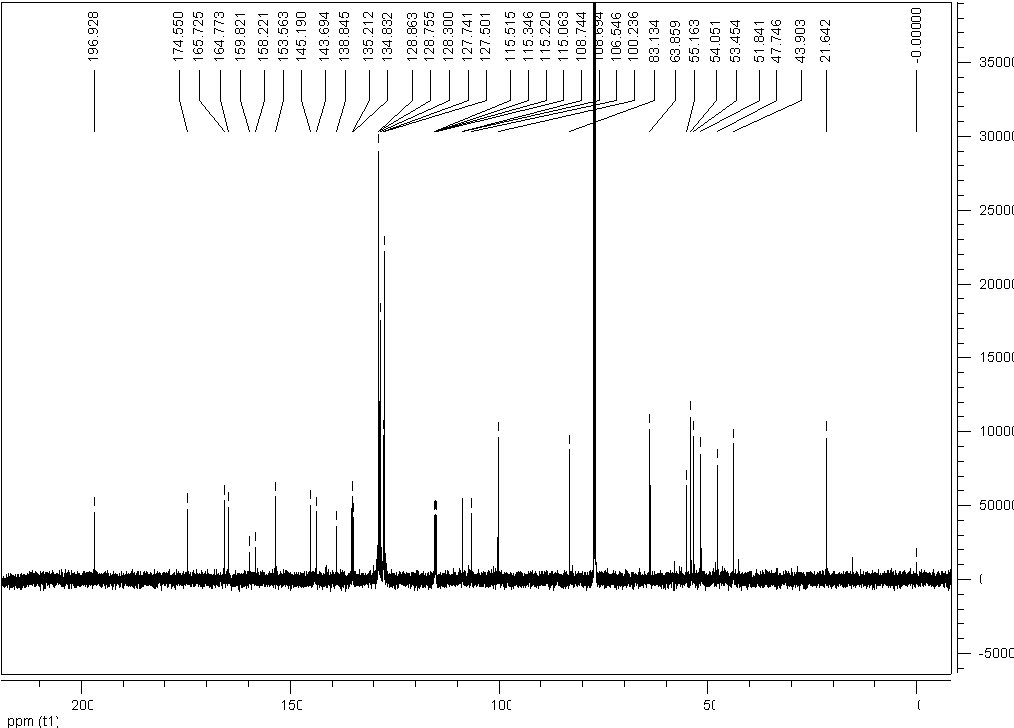


**dimethyl 1-benzyl-5-fluoro-8'-methoxy-2'-benzoyl-2-oxo-2',9a'-dihydrospiro[indoline-3,1'-**

**quinolizine]-3',4'-dicarboxylate (1m)**: ellow solid, 81%, m.p. 137.2~137.6℃; ^1^H NMR (600 MHz, DMSO-*d_6_*) δ: 7.63 (brs, 1H, ArH), 7.46~7.44 (m, 4H, ArH), 7.17 (brs, 3H, ArH), 7.08 (brs, 1H, ArH), 7.02 (brs, 2H, ArH), 6.83~6.77 (m, 2H, ArH), 6.44 (d, *J* = 8.4Hz, 1H, CH), 5.39 (s, 1H, CH), 5.06 (brs, 1H, CH), 4.66 (d, *J* = 5.4Hz, 1H, CH), 4.46 (d, *J* = 15.0Hz, 1H, CH), 4.36 (d, *J* = 15.0Hz, 1H, CH), 3.96 (s, 3H, OCH_3_), 3.75 (brs, 1H, CH), 3.50 (s, 3H, OCH_3_), 2.88 (s, 3H, OCH_3_); ^13^C NMR (150 MHz, CDCl_3_) δ: 197.5, 174.5, 165.7, 164.7, 159.0 (d, *J* = 239.9Hz), 153.6, 145.2, 138.9, 137.5, 135.3, 132.9, 128.8, 128.7, 128.2, 128.1, 127.8, 127.6, 115.4 (d, *J* = 21.2Hz), 115.2 (d, *J* = 19.7Hz), 108.7 (d, *J* = 8.4Hz), 106.3, 100.3, 83.1, 63.8, 55.1, 54.0, 53.5, 51.9, 48.1, 43.9; IR (KBr) υ: 3450, 2948, 1752, 1712, 1647, 1631, 1598, 1488, 1436, 1388, 1335, 1296, 1227, 1155, 1131, 1051, 980, 940, 908, 894, 862, 826, 768cm^-1^; MS (*m*/*z*): HRMS (ESI) Calcd. for C_35_H_30_FN_2_O_7_ ([M+H]^+^): 609.2032. Found: 609.2034.


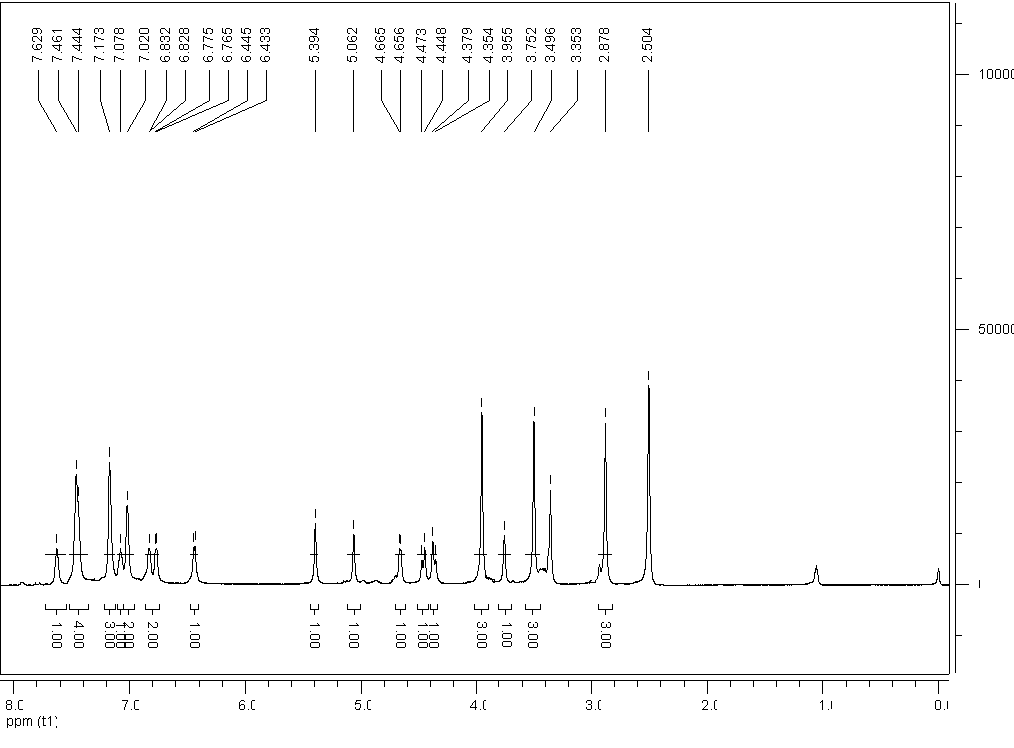


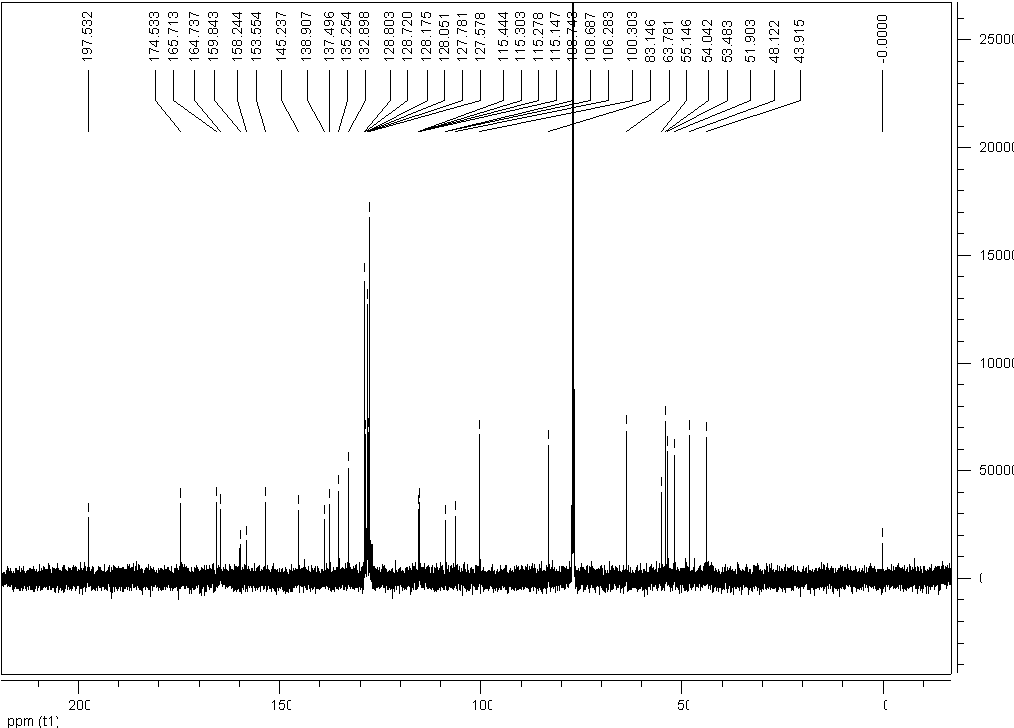


**dimethyl 1-butyl-5-chloro-8'-methoxy-2'-(4-methylbenzoyl)-2-oxo-2',9a'-dihydrospiro[indo-**

**line-3,1'-quinolizine]-3',4'-dicarboxylate (1n)**: yellow solid, 93%, m.p. 162.1~163.0℃; ^1^H NMR (600 MHz, DMSO-*d_6_*) δ: 7.41 (d, *J* = 8.4Hz, 2H, ArH), 7.30 (dd, *J_1_* = 8.4Hz, *J_2_* = 1.8Hz, 1H, ArH), 6.98 (d, *J* = 1.8Hz, 1H, ArH), 6.90 (dd, *J_1_* = 5.7Hz, *J_2_* = 3.0Hz, 3H, ArH), 6.43 (d, *J* = 7.8Hz, 1H, CH), 5.26 (s, 1H, CH), 4.98 (d, *J* = 3.0Hz, 1H, CH), 4.70 (dd, *J_1_* = 7.8Hz, *J_2_* = 1.8Hz, 1H, CH), 3.95 (s, 3H, OCH_3_), 3.85 (brs, 1H, CH), 3.79 (s, 3H, OCH_3_), 3.47 (s, 3H, OCH_3_), 3.38~3.33 (m, 1H, CH), 3.30~3.27 (m, 1H, CH), 3.18 (s, 3H, OCH_3_), 1.10~1.05 (m, 1H, CH), 1.00~0.96 (m, 2H, CH), 0.94~0.89 (m, 1H, CH), 0.71 (t, *J* = 7.2Hz, 3H, CH_3_); ^13^C NMR (150 MHz, CDCl_3_) δ: 195.7, 174.1, 165.7, 164.8, 163.3, 163.2, 153.6, 145.2, 141.8, 130.4, 128.8, 128.6, 128.0, 127.7, 125.8, 113.6, 113.3, 108.4, 106.8, 102.5, 100.1, 83.1, 63.7, 57.6, 55.4, 55.2, 54.1, 53.4, 51.8, 47.5, 39.9, 29.3, 20.1, 13.6; IR (KBr) υ: 3450, 2953, 1737, 1713, 1670, 1626, 1601, 1574, 1510, 1483, 1459, 1432, 1384, 1323, 1246, 1175, 1133, 1116, 1027, 979, 940, 915, 873, 848, 812, 780cm^-1^; MS (*m*/*z*): HRMS (ESI) Calcd. for C_33_H_34_ClN_2_O_8_ ([M+H]^+^): 621.1998. Found: 621.1997.


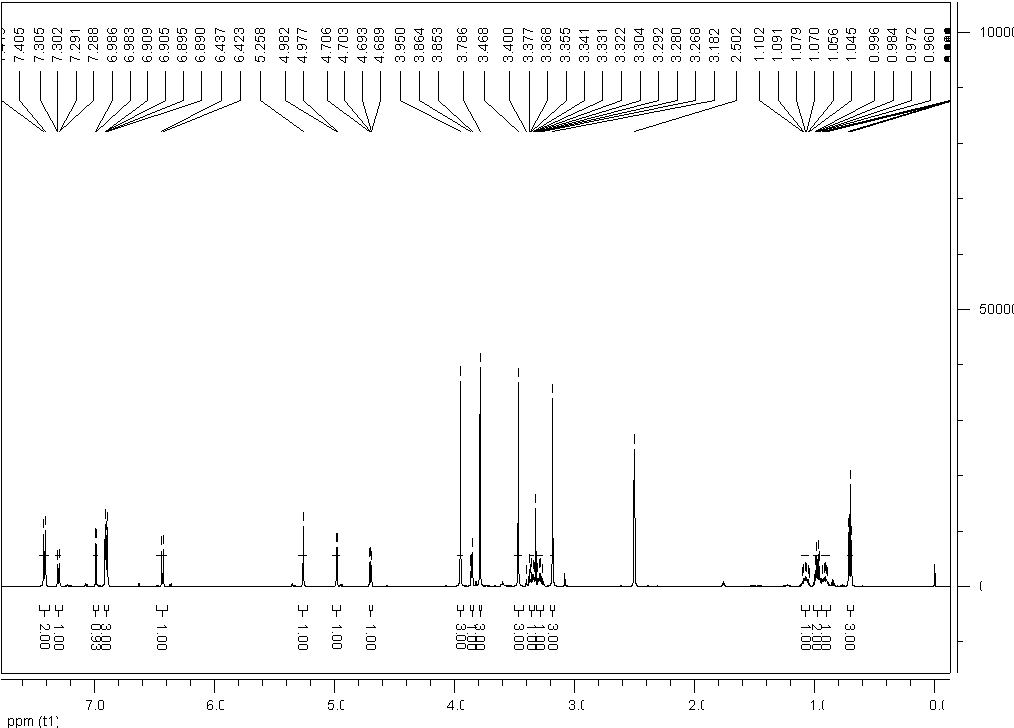


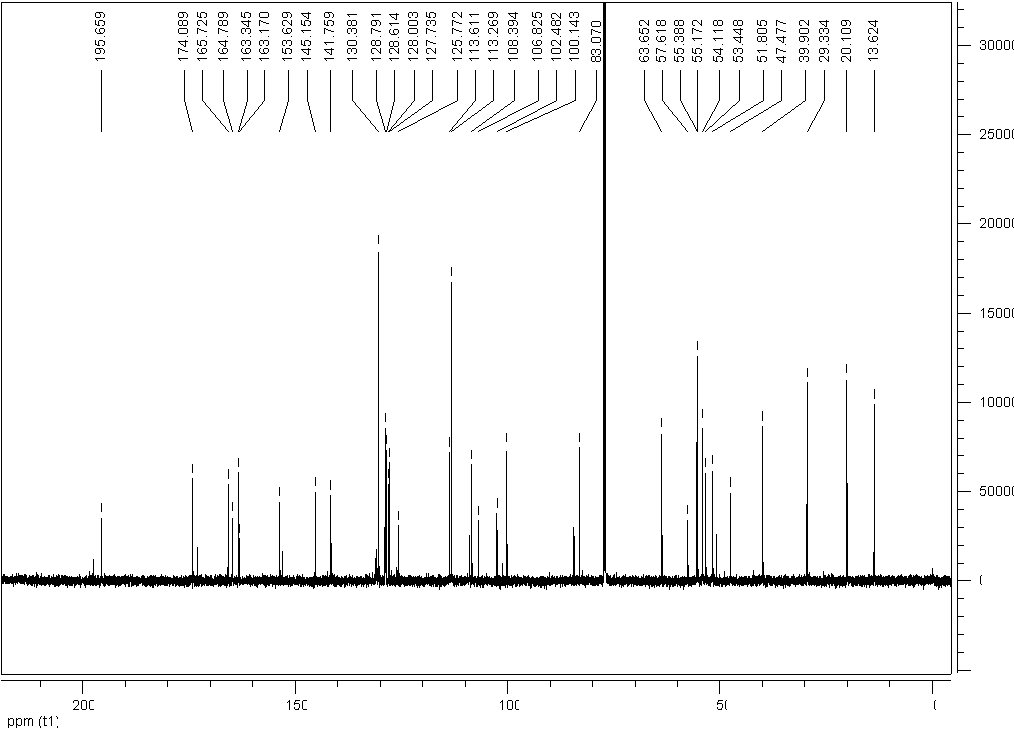


**dimethyl 1-butyl-5-fluoro-8'-methoxy-2'-(4-methylbenzoyl)-2-oxo-2',9a'-dihydrospiro[indo-**

**line-3,1'-quinolizine]-3',4'-dicarboxylate (1o)**: yellow solid, 90%, m.p. 176.7~177.2℃; ^1^H NMR (600 MHz, DMSO-*d_6_*) δ: 7.28 (d, *J* = 8.4Hz, 2H, ArH), 7.17 (d, *J* = 7.8Hz, 2H, ArH), 7.10 (td, *J_1_* = 9.0Hz, *J_2_* = 2.4Hz, 1H, ArH), 6.87 (dd, *J_1_* = 8.4Hz, *J_2_* = 4.2Hz, 1H, ArH), 6.75 (dd, *J_1_* = 8.4Hz, *J_2_* = 2.4Hz, 1H, ArH), 6.43 (d, *J* = 7.8Hz, 1H, CH), 5.27 (s, 1H, CH), 4.97 (d, *J* = 3.6Hz, 1H, CH), 4.70 (dd, *J_1_* = 8.4Hz, *J_2_* = 2.4Hz, 1H, CH), 3.95 (s, 3H, OCH_3_), 3.86 (brs, 1H, CH), 3.48 (s, 3H, OCH_3_), 3.30~3.20 (m, 2H, CH), 3.18 (s, 3H, OCH_3_), 2.31 (s, 3H, CH_3_), 1.09~1.03 (m, 1H, CH), 1.02~0.97 (m, 2H, CH), 0.92~0.85 (m, 1H, CH), 0.72 (t, *J* = 7.2Hz, 3H, CH_3_); ^13^C NMR (150 MHz, CDCl_3_) δ: 197.2, 174.1, 165.7, 164.8, 158.9 (d, *J* = 239.4Hz), 153.5, 145.2, 143.5, 139.2, 135.0, 129.1, 128.8, 128.3, 128.1, 115.4 (d, *J* = 25.1Hz), 107.9 (d, *J* = 8.3Hz), 106.6, 102.6, 100.2, 83.2, 63.6, 57.6, 55.1, 54.1, 53.4, 51.8, 47.8, 39.9, 29.2, 21.6, 20.1, 13.7; IR (KBr) υ: 3452, 2951, 1751, 1709, 1676, 1629, 1599, 1491, 1456, 1437, 1378, 1322, 1275, 1229, 1197, 1181, 1159, 1134, 1048, 1005, 975, 940, 901, 868, 839, 823, 761cm^-1^; MS (*m*/*z*): HRMS (ESI) Calcd. for C_33_H_34_FN_2_O_7_ ([M+H]^+^): 589.2345. Found: 589.2345.


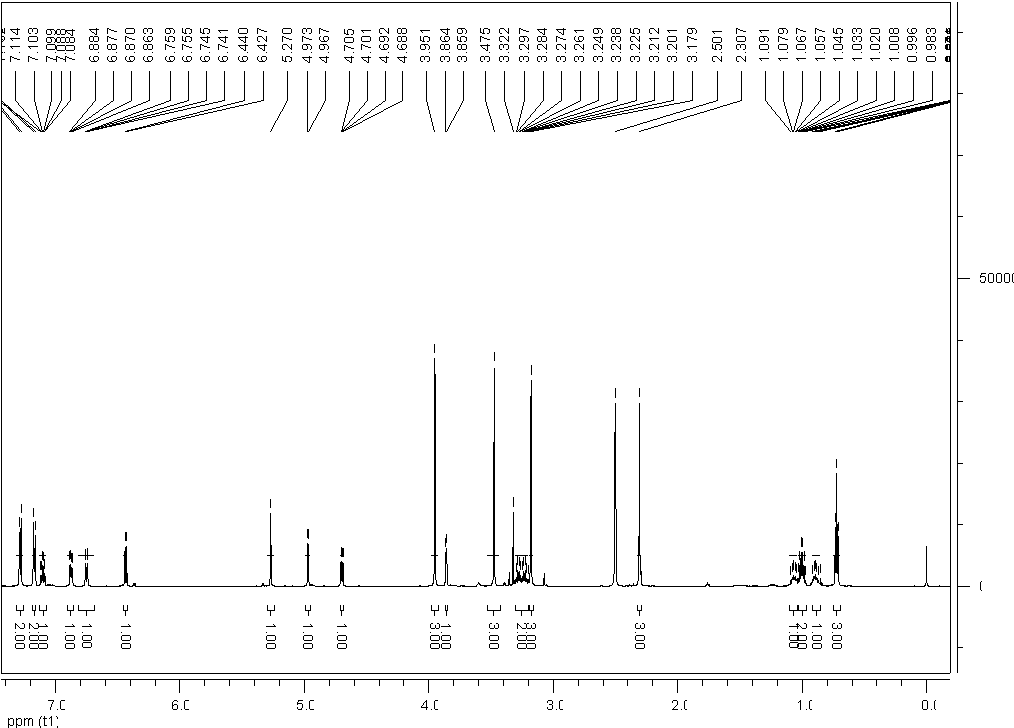


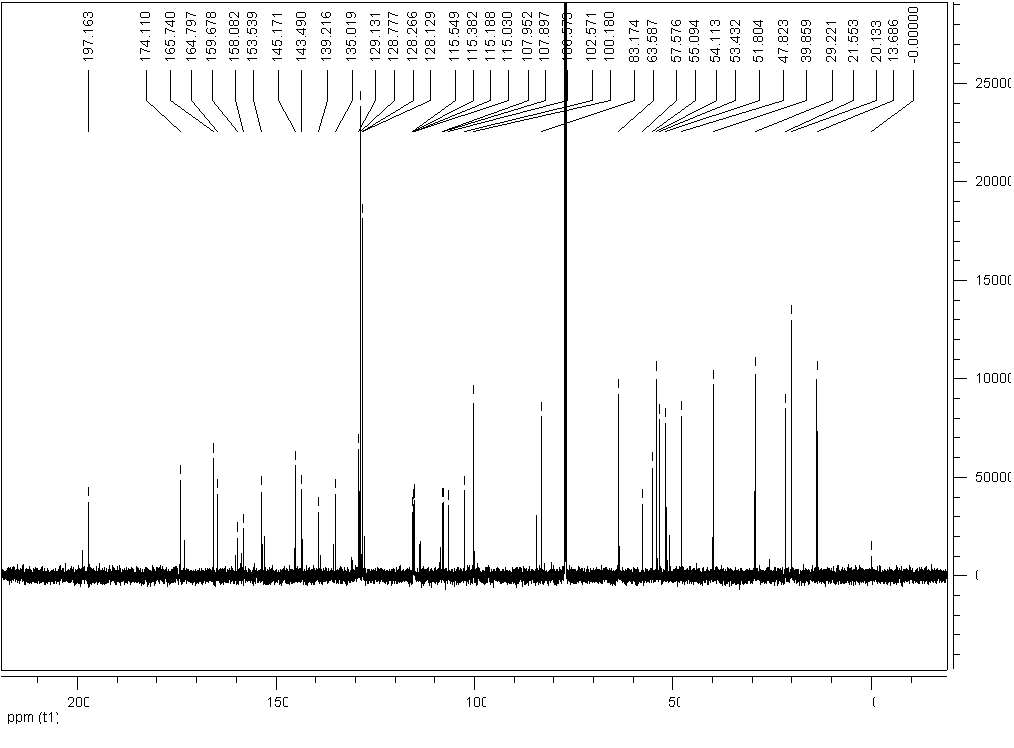


**dimethyl 1-benzyl-2'-(4-methylbenzoyl)-2,8'-dioxo-2',8',9',9a'-tetrahydrospiro[indoline-3,1'-**

**quinolizine]-3',4'-dicarboxylate (2a)**: white solid, m.p. 188.8~188.9℃; ^1^H NMR (600 MHz, DMSO-*d_6_*) δ: 7.59 (brs, 2H, ArH), 7.36 (brs, 1H, ArH), 7.19~7.13 (m, 8H, ArH), 6.80 (brs, 2H, ArH), 6.56 (brs, 1H, CH), 5.28 (brs, 2H, CH), 4.46 (d, *J* = 13.2Hz, 1H, CH), 4.39 (d, *J* = 15.6Hz, 1H, CH), 4.29 (d, *J* = 15.6Hz, 1H, CH), 4.06 (s, 3H, OCH_3_), 3.51 (s, 3H, OCH_3_), 2.40 (s, 3H, CH_3_), 2.22 (d, *J* = 15.6Hz, 1H, CH), 1.89 (t, *J* = 15.6Hz, 1H, CH); ^13^C NMR (150 MHz, CDCl_3_) δ: 195.4, 190.3, 174.3, 165.3, 164.0, 144.4, 142.7, 134.6, 134.0, 129.6, 129.2, 128.8, 128.6, 127.7, 127.1, 127.0, 124.2, 123.9, 109.6, 107.3, 106.0, 59.0, 53.8, 52.0, 51.8, 45.9, 44.2, 36.1, 21.7; IR (KBr) υ: 3453, 1752, 1715, 1664, 1637, 1593, 1487, 1466, 1453, 1367, 1326, 1292, 1254, 1218, 1183, 1129, 1106, 945, 904, 795, 754cm^-1^; MS (*m*/*z*): HRMS (ESI) Calcd. for C_35_H_30_N_2_NaO_7_ ([M+Na]^+^): 613.1945. Found: 613.1947.


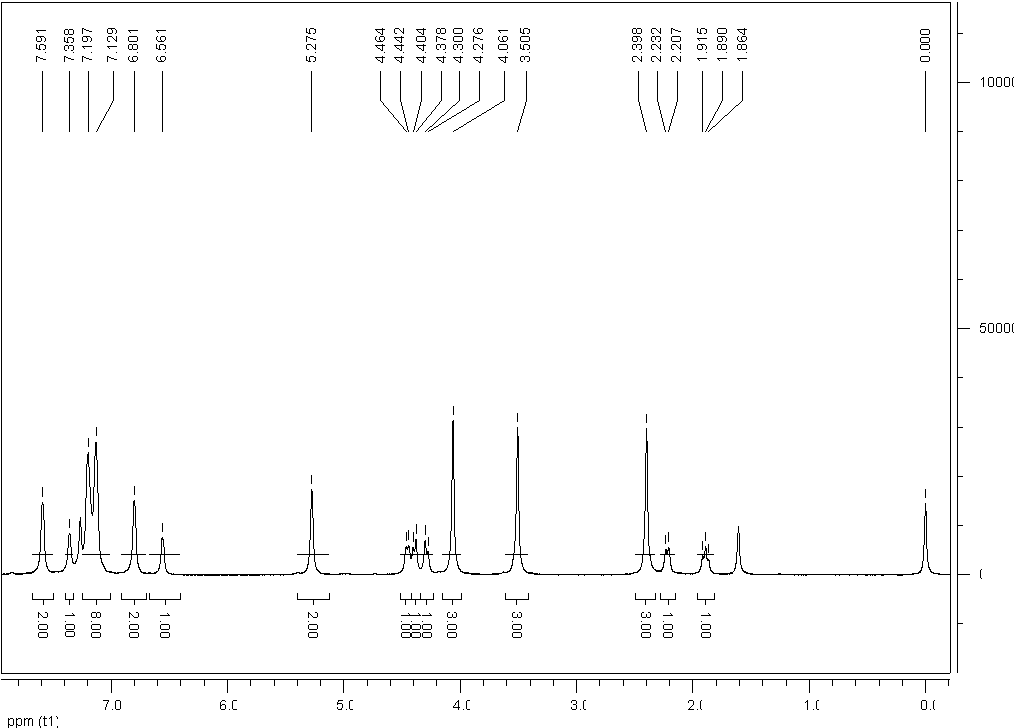


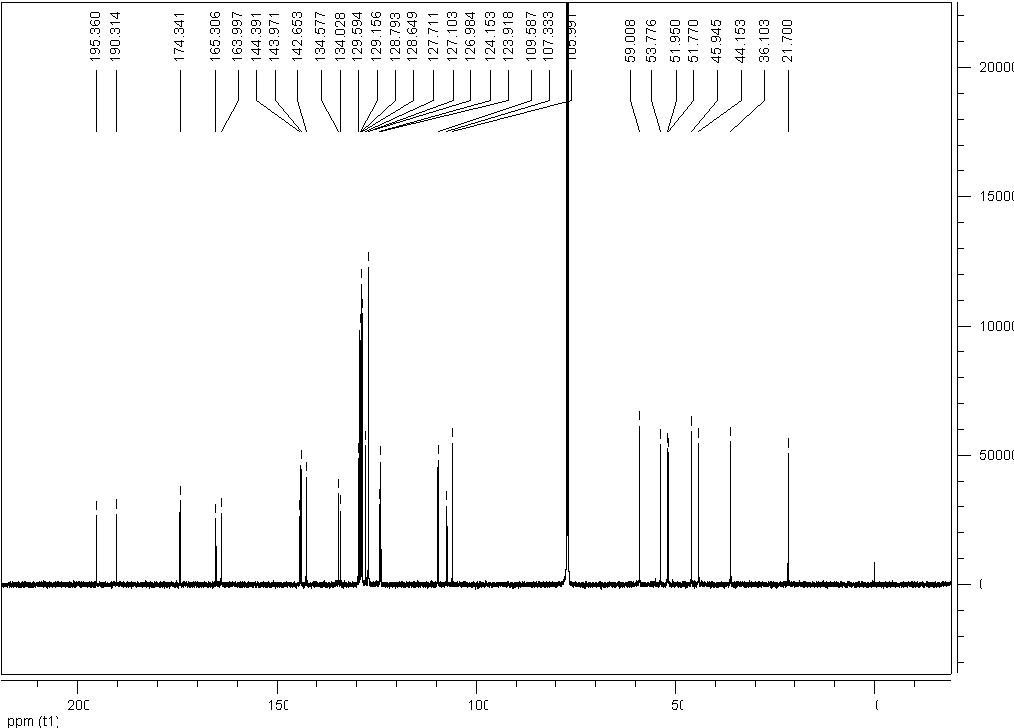


**dimethyl 1-benzyl-5-chloro-2'-(4-methylbenzoyl)-2,8'-dioxo-2',8',9',9a'-tetrahydrospiro[indo-**

**line-3,1'-quinolizine]-3',4'-dicarboxylate (2b)**: white solid, m.p. 181.1~181.3℃; ^1^H NMR (600 MHz, DMSO-*d_6_*) δ: 7.89 (brs, 2H, ArH), 7.59 (brs, 1H, ArH), 7.47 (brs, 1H, ArH), 7.29 (brs, 5H, ArH), 7.13 (brs, 4H, ArH, CH), 5.34 (s, 1H, CH), 4.91 (brs, 2H, CH), 4.66~4.63 (m, 2H, CH), 3.99 (s, 3H, OCH_3_), 3.47 (s, 3H, OCH_3_), 2.38 (s, 3H, CH_3_), 1.87 (brs, 2H, CH); ^13^C NMR (150 MHz, CDCl_3_) δ: 197.6, 190.3, 172.4, 164.9, 164.0, 144.7, 144.2, 144.0, 141.0, 135.1, 134.7, 129.8, 129.6, 129.4, 128.9, 128.7, 128.1, 127.6, 125.5, 110.7, 107.7, 102.6, 54.9, 53.8, 52.3, 51.0, 44.2, 43.3, 36.3, 21.7; IR (KBr) υ: 3452, 2956, 1740, 1713, 1678, 1633, 1594, 1479, 1439, 1383, 1326, 1300, 1249, 1186, 1131, 1081, 1002, 956, 936, 898, 883, 855, 815, 781, 737, 704cm^-1^; MS (*m*/*z*): HRMS (ESI) Calcd. for C_35_H_30_ClN_2_O_7_ ([M+H]^+^): 625.1736. Found: 625.1745.


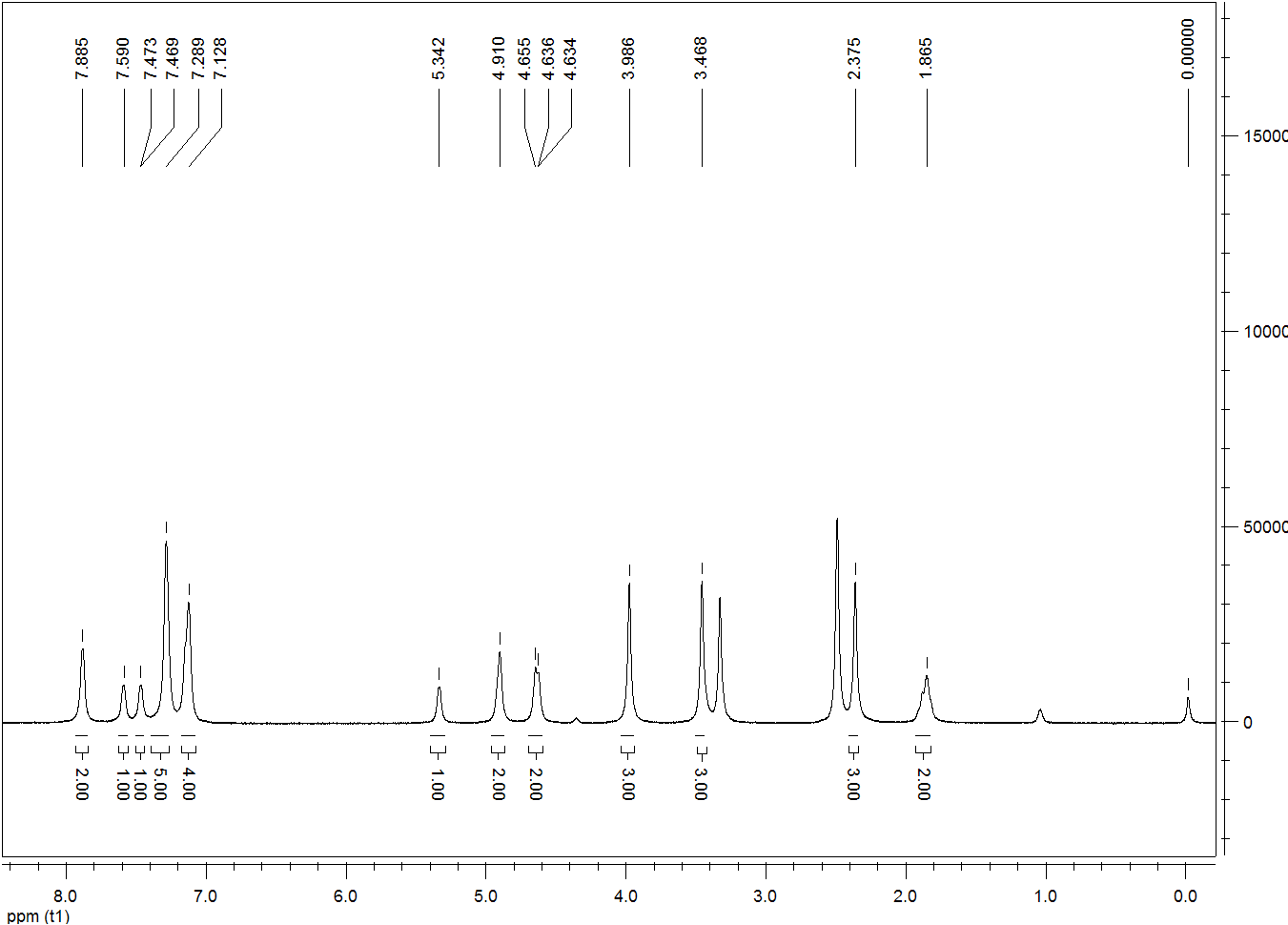


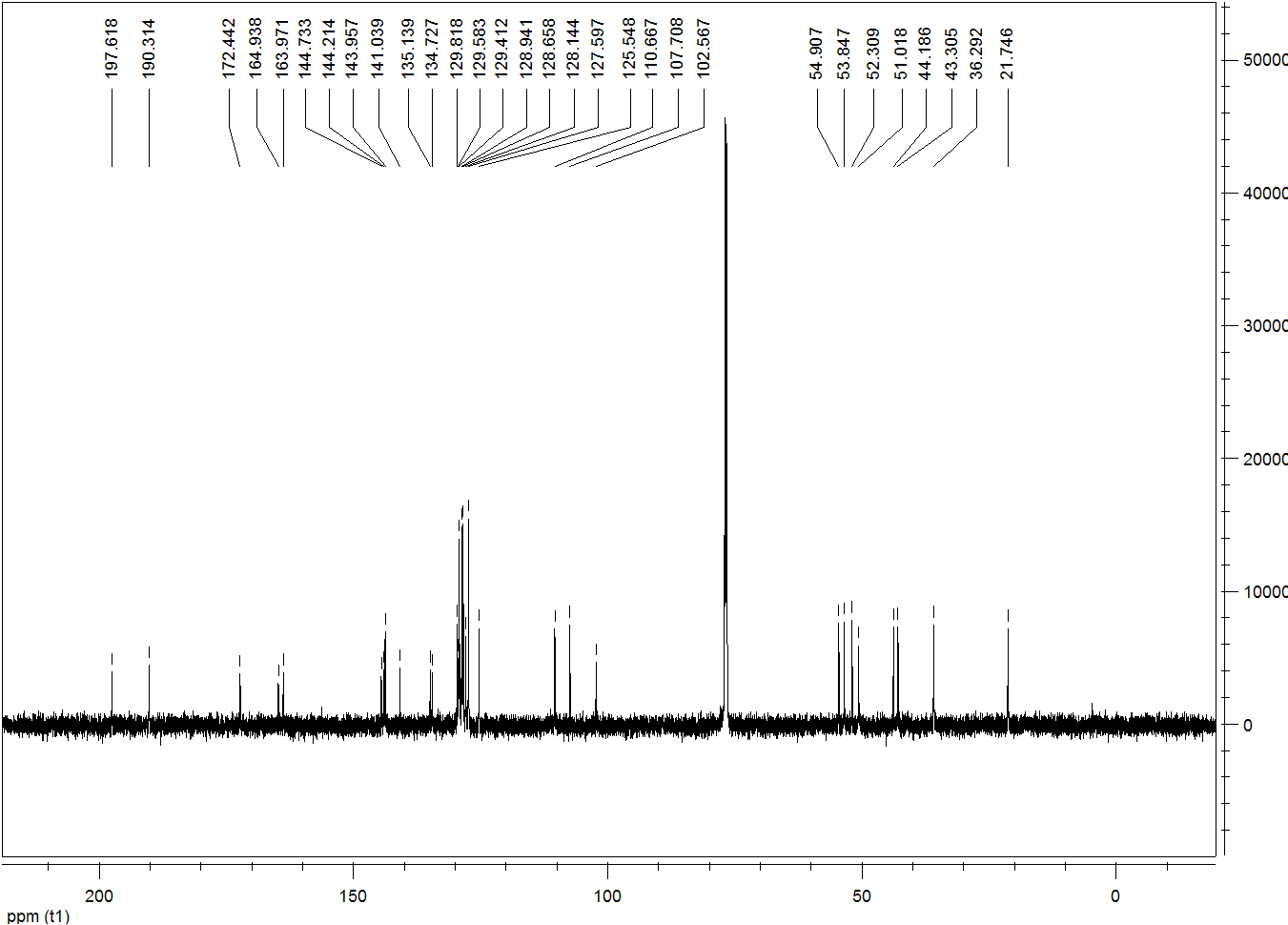


**dimethyl 1-benzyl-5-chloro-3'-(4-methylbenzoyl)-2-oxo-3',4a'-dihydrospiro[indoline-3,4'-**

**pyrido[1,2-a]quinoline]-1',2'-dicarboxylate (3a)**: yellow solid, 40%, m.p. 183~186℃; ^1^H NMR (600 MHz, DMSO-*d_6_*) δ: 7.36 (brs, 2H, ArH), 7.23 (brs, 2H, ArH), 7.16 (brs, 4H, ArH), 6.98~6.93 (m, 2H, ArH), 6.86 (brs, 4H, ArH), 6.65 (brs, 1H, ArH), 6.57 (brs, 1H, ArH), 6.33 (d, *J* = 5.4Hz, 1H, CH), 5.42 (s, 1H, CH), 5.33 (brs, 1H, CH), 4.72~4.61 (brs, 3H, CH), 3.88 (s, 3H, OCH_3_), 3.61 (s, 3H, OCH_3_), 2.33 (s, 3H, CH_3_); ^13^C NMR (150 MHz, CDCl_3_) δ: 195.4, 173.8, 166.0, 165.2, 146.1, 143.7, 141.5, 138.1, 134.7, 134.6, 129.9, 129.2, 129.1, 128.7, 128.5, 128.4, 128.1, 127.8, 127.7, 126.9, 126.7, 122.4, 121.6, 118.9, 118.0, 114.7, 109.2, 65.0, 59.3, 53.1, 52.3, 49.4, 44.1; IR (KBr) υ: 3448, 2949, 1721, 1701, 1683, 1640, 1571, 1490, 1432, 1382, 1356, 1302, 1242, 1179, 1114, 972, 817 cm^-1^; MS (*m*/*z*): HRMS (ESI) Calcd. for C_39_H_32_ClN_2_O_6_ ([M+H]^+^): 659.1943. Found: 659.1941.

**
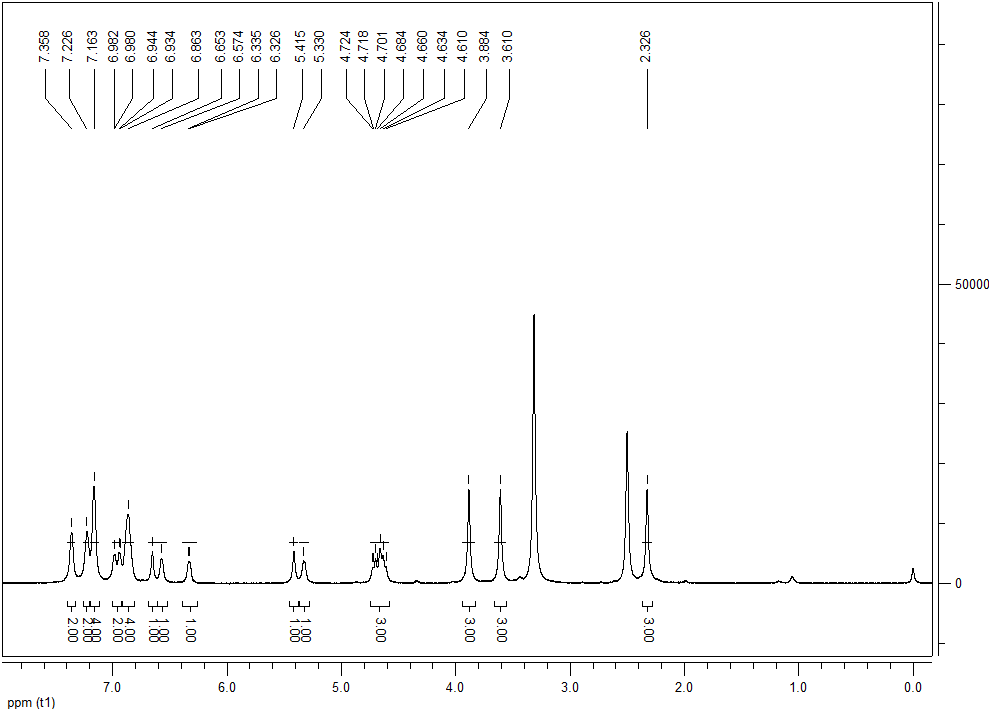
**

**
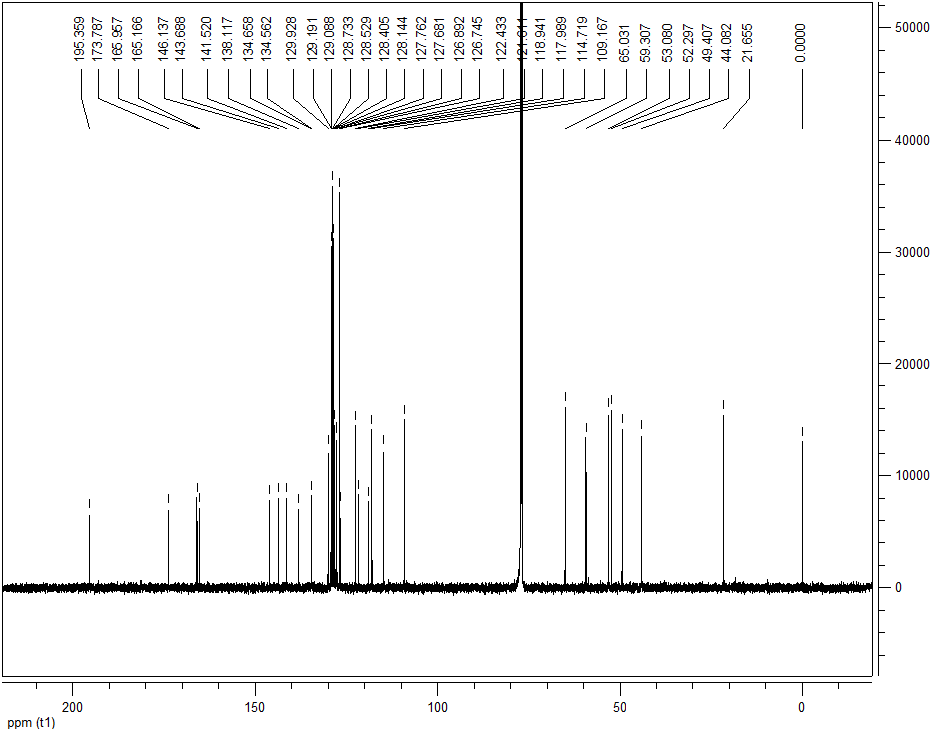
**

**dimethyl 1-benzyl-5-chloro-3'-(4-chlorobenzoyl)-2-oxo-3',4a'-dihydrospiro[indoline-3,4'-**

**pyrido[1,2-a]quinoline]-1',2'-dicarboxylate (3b)**：yellow solid, 52%, m.p. 171~173℃; ^1^H NMR (600 MHz, DMSO-*d_6_*) δ: 7.45 (brs, 4H, ArH), 7.22 (brs, 4H, ArH), 7.02 (brs, 1H, ArH), 6.87 (brs, 5H, ArH), 6.66 (brs, 2H, ArH), 6.35 (brs, 1H, CH), 5.45 (brs, 1H, CH), 5.34 (brs, 1H, CH), 4.76~4.64 (brs, 3H, CH), 3.89 (s, 3H, OCH_3_), 3.62 (s, 3H, OCH_3_); ^13^C NMR (150 MHz, DMSO-*d_6_*) δ: 194.8, 172.6, 165.4, 164.4, 150.5, 144.9, 141.7, 138.6, 137.5, 135.9, 135.2, 135.0, 129.8, 129.5, 128.9, 128.5, 128.3, 128.1, 128.0, 127.9, 127.4, 127.0, 126.1, 125.8, 122.4, 121.4, 118.6, 118.3, 113.9, 110.1, 64.3, 58.0, 53.1, 48.7, 43.2; IR (KBr) υ: 3448, 2949, 1722, 1702, 1641, 1614, 1490, 1433, 1401, 1382, 1356, 1304, 1241, 1190, 1130, 1090, 1015, 968, 917, 850, 817 cm^-1^; MS (*m*/*z*): HRMS (ESI) Calcd. for C_38_H_29_Cl_2_N_2_O_6_ ([M+H]^+^): 679.1397. Found: 679.1392.

**
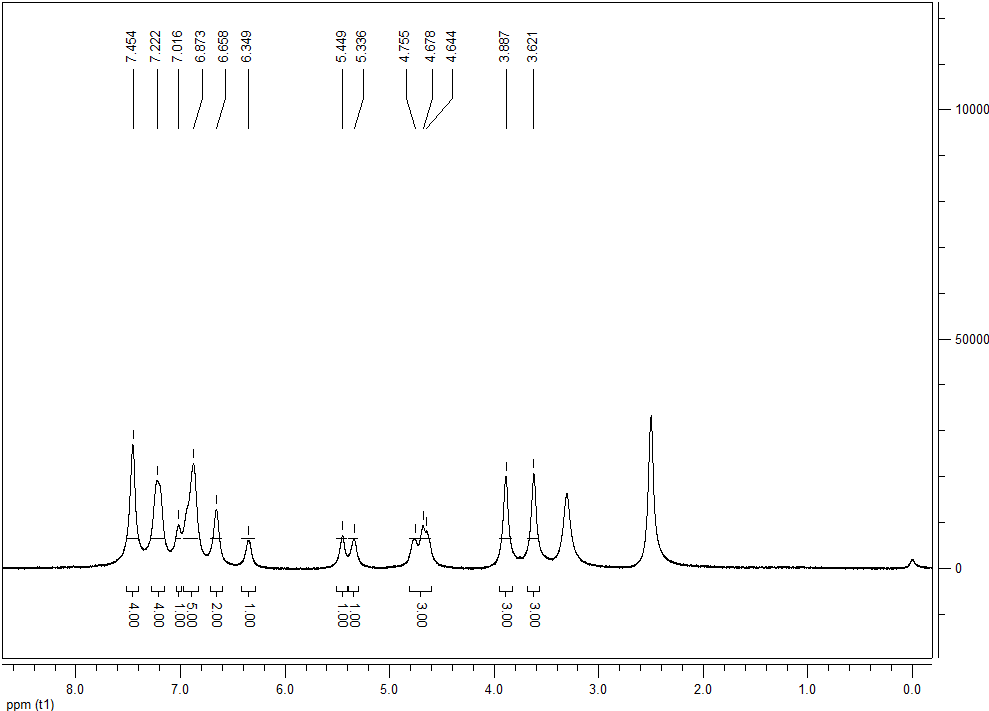
**

**
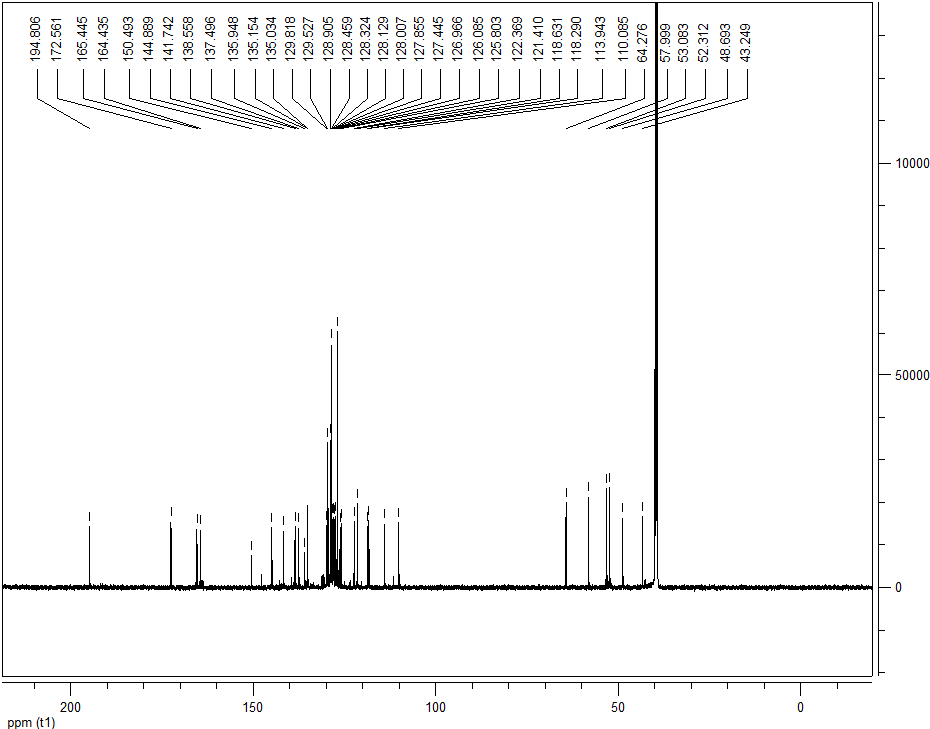
**

**dimethyl 1-benzyl-5-fluoro-3'-benzoyl-2-oxo-3',4a'-dihydrospiro[indoline-3,4'-pyrido[1,2-a]-**

**quinoline]-1',2'-dicarboxylate (3c)**：yellow solid, 55%, m.p. 166~167℃; ^1^H NMR (600 MHz, DMSO-*d_6_*) δ: 7.58 (brs, 1H, ArH), 7.41~7.36 (m, 4H, ArH), 7.21 (brs, 4H, ArH), 6.95~6.80 (m, 6H, ArH), 6.56 (brs, 1H, ArH), 6.41~6.40 (m, 1H, ArH), 6.34~6.33 (m, 1H, CH), 5.44 (s, 1H, CH), 5.32 (d, *J* = 6.6Hz, 1H, CH), 4.68~4.61 (m, 3H, CH), 3.89 (s, 3H, OCH_3_), 3.63 (s, 3H, OCH_3_); ^13^C NMR (150 MHz, DMSO-*d_6_*) δ: 195.8, 172.9, 165.5, 164.5, 157.2 (d, *J* = 236.4Hz), 150.5, 145.0, 139.3, 137.5, 136.5, 136.0, 135.3, 133.4, 129.8, 128.6, 127.9, 127.8, 127.6, 127.4, 126.9, 126.2 (d, *J* = 8.7Hz), 122.3, 121.4, 118.7, 118.5, 115.5 (d, *J* = 25.7Hz), 114.8 (d, *J* = 23.0Hz), 113.9, 109.5 (d, *J* = 8.3Hz), 64.3, 58.4, 53.1, 52.3, 48.9, 43.3; IR (KBr) υ: 3448, 2952, 1737, 1711, 1633, 1605, 1571, 1491, 1437, 1407, 1383, 1360, 1180, 1114, 1081, 1014, 967, 875, 819, 768 cm^-1^; MS (*m*/*z*): HRMS (ESI) Calcd. for C_38_H_30_FN_2_O_6_ ([M+H]^+^): 629.2082. Found: 629.2078.

**
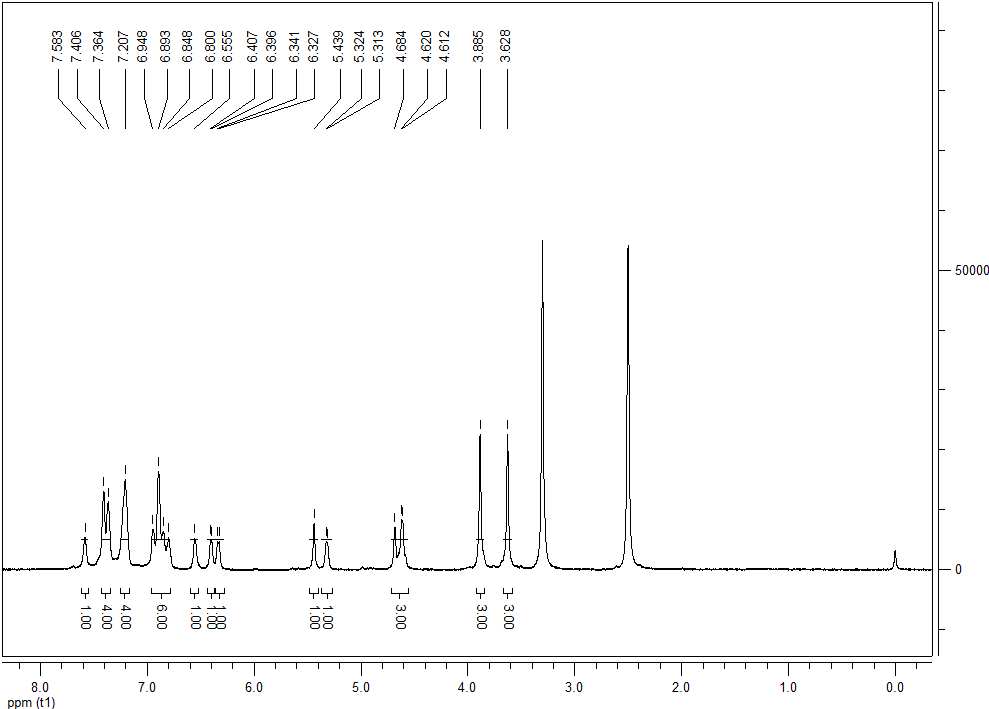
**

**
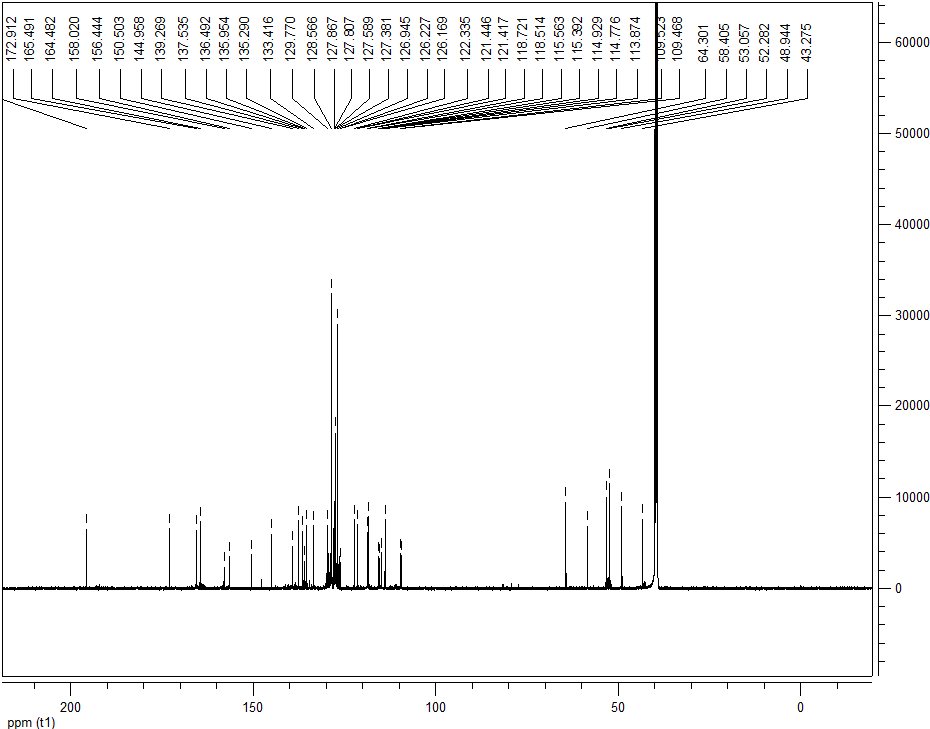
**

**dimethyl 1-benzyl-5-fluoro-3'-(4-chlorobenzoyl)-2-oxo-3',4a'-dihydrospiro[indoline-3,4'-pyrido[1,2-a]quinoline]-1',2'-dicarboxylate (3d)**：yellow solid, 53%, m.p. 170~171℃; ^1^H NMR (600 MHz, DMSO-*d_6_*) δ: 7.45 (brs, 4H, ArH), 7.22 (brs, 4H, ArH), 6.90 (brs, 6H, ArH), 6.65 (brs, 1H, ArH), 6.42~6.35 (m, 2H, ArH, CH), 5.44 (s, 1H, CH), 5.35 (brs, 1H, CH), 4.76~4.65 (m, 3H, CH), 3.88 (s, 3H, OCH_3_), 3.62 (s, 3H, OCH_3_); ^13^C NMR (150 MHz, DMSO-*d_6_*) δ: 194.8, 172.8, 165.5, 164.4, 157.3 (d, *J* = 239.1Hz), 144.9, 139.2, 138.5, 137.5, 135.3, 135.1, 129.8, 129.5, 128.9, 128.5, 127.9, 127.8, 127.4, 127.0, 126.0 (d, *J* = 9.2Hz), 122.4, 121.5, 118.5, 115.6 (d, *J* = 17.0Hz), 115.0 (d, *J* = 18.6Hz), 113.9, 109.6, 64.3, 58.1, 53.1, 52.3, 48.7, 43.3; IR (KBr) υ: 3450, 2949, 1712, 1634, 1570, 1491, 1454, 1431, 1405, 1382, 1351, 1245, 1178, 1092, 967, 852, 820, 776 cm^-1^; MS (*m*/*z*): HRMS (ESI) Calcd. for C_38_H_29_ClFN_2_O_6_ ([M+H]^+^): 663.1693. Found: 663.1692.

**
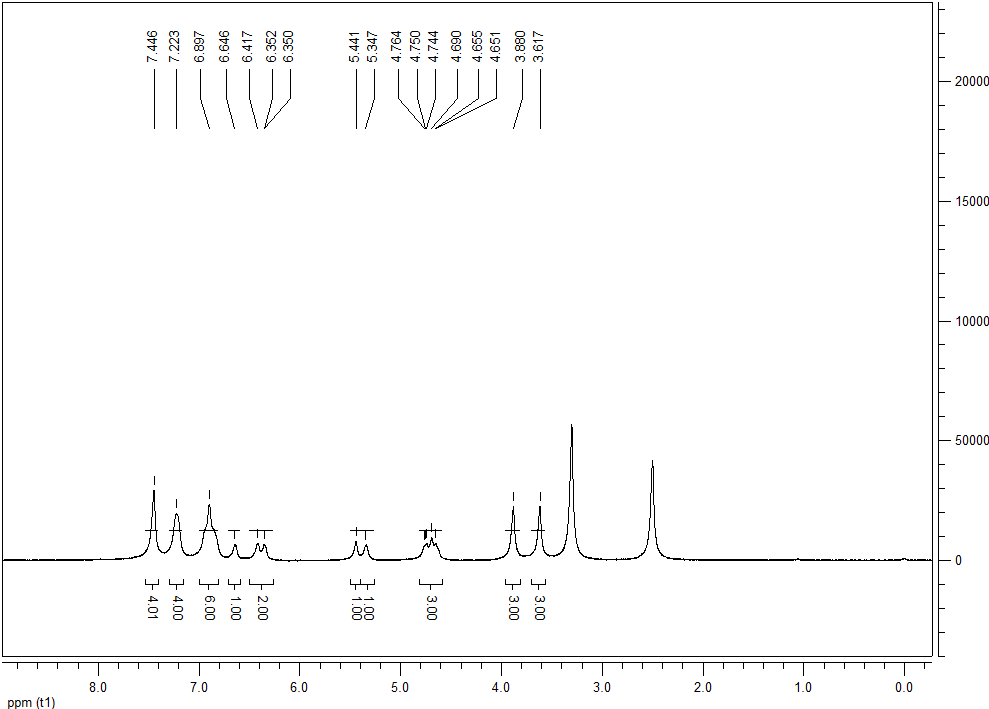
**

**
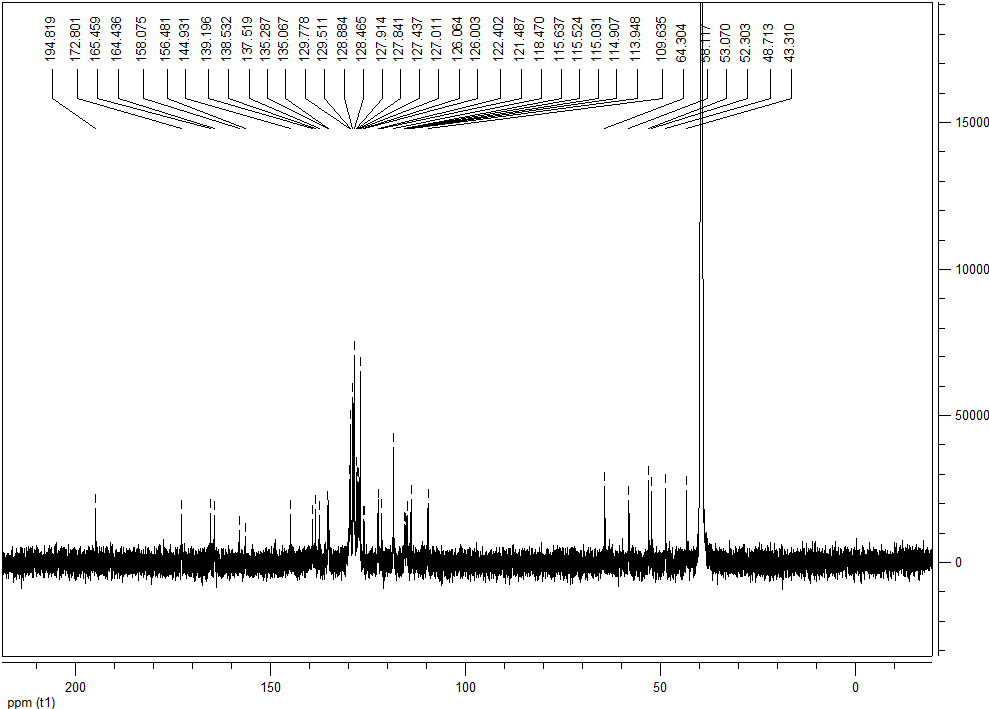
**

**dimethyl 1-butyl-5-chloro-3'-(4-methoxybenzoyl)-2-oxo-3',4a'-dihydrospiro[indoline-3,4'-**

**pyrido[1,2-a]quinoline]-1',2'-dicarboxylate (3e)**：yellow solid, 60%, m.p. 186~189℃; ^1^H NMR (600 MHz, DMSO-*d_6_*) δ: 7.34 (brs, 2H, ArH), 7.22 (brs, 1H, ArH), 7.05 (brs, 1H, ArH), 6.93 (brs, 1H, ArH), 6.87 (brs, 4H, ArH), 6.76 (brs, 1H, ArH), 6.62 (brs, 1H, ArH), 6.35 (brs, 1H, CH), 5.44 (s, 1H, CH), 5.32~5.29 (m, 2H, CH), 4.59 (s, 1H, CH), 3.88 (s, 3H, OCH_3_), 3.76 (s, 3H, OCH_3_), 3.61 (s, 3H, OCH_3_), 3.44 (brs, 2H, CH), 1.03~1.02 (m, 4H, CH), 0.76 (brs, 3H, CH_3_); ^13^C NMR (150 MHz, DMSO-*d_6_*) δ: 194.1, 172.5, 165.5, 164.6, 163.2, 144.9, 142.2, 137.6, 129.9, 129.8, 129.5, 128.1, 128.0, 127.8, 126.5, 125.3, 122.2, 121.4, 118.9, 118.4, 113.8, 113.7, 109.4, 64.0, 58.3, 55.4, 53.0, 52.2, 48.9, 28.7, 19.4, 13.5; IR (KBr) υ: 3452, 2947, 1739, 1716, 1680, 1603, 1572, 1490, 1434, 1381, 1355, 1309, 1252, 1210, 1179, 1135, 1022, 968, 883, 816, 779 cm^-1^; MS (*m*/*z*): HRMS (ESI) Calcd. for C_36_H_34_ClN_2_O_7_ ([M+H]^+^): 641.2049. Found: 649.2045.

**
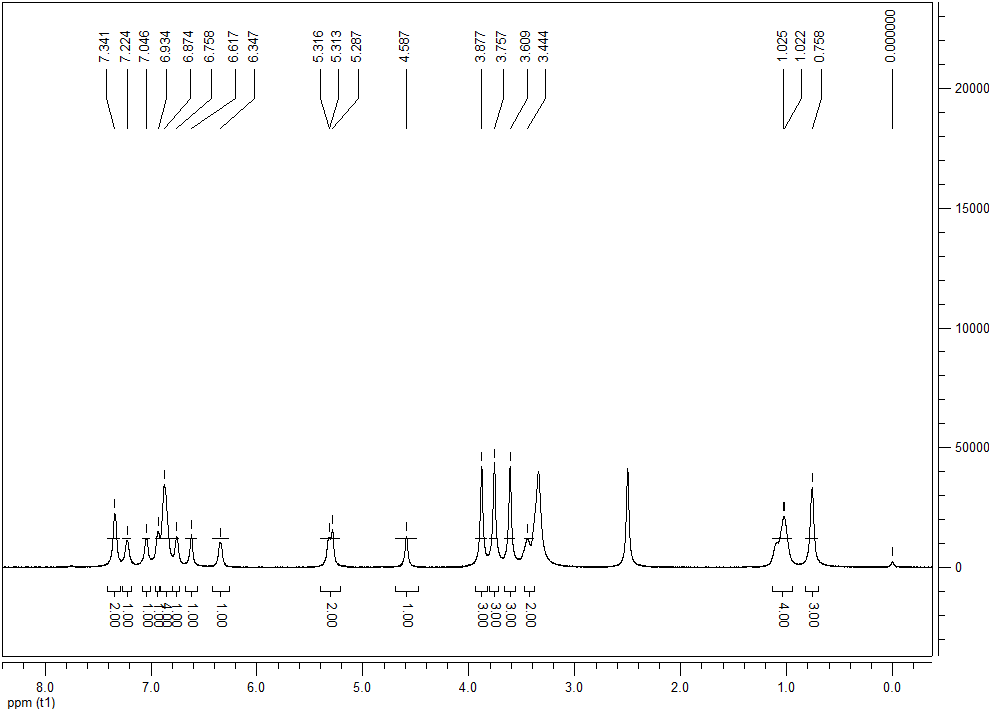
**

**
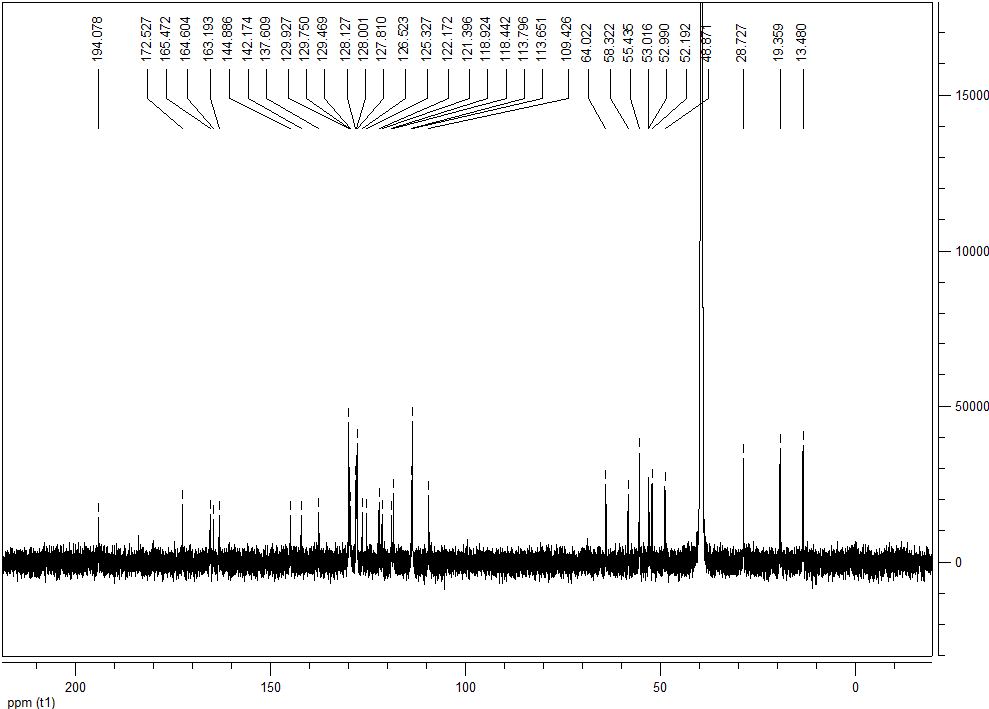
**

**3'-ethyl 1',2'-dimethyl 1-benzyl-5-methyl-2-oxo-3',4a'-dihydrospiro[indoline-3,4'-pyrido-**

**[1,2-a]quinoline]-1',2',3'-tricarboxylate (3f)**: yellow solid, 50%, m.p. 117~115℃; ^1^H NMR (600 MHz, DMSO-*d_6_*) δ: 7.38~7.18 (m, 6H, ArH), 6.94~6.82 (m, 5H, ArH), 6.52 (brs, 1H, ArH), 6.29 (brs, 1H, CH), 5.35 (brs, 1H, CH), 5.02 (brs, 1H, CH), 4.88 (brs, 1H, CH), 4.50 (s, 1H, CH), 4.26 (s, 1H, CH), 3.87 (s, 3H, OCH_3_), 3.70 (s, 3H, OCH_3_), 3.35~3.54 (m, 2H, CH_2_), 1.74 (s, 3H, CH_3_), 0.38~0.37 (m, 3H, CH_3_); ^13^C NMR (150 MHz, DMSO-*d_6_*) δ: 173.2, 168.1, 165.3, 164.7, 144.8, 141.1, 137.7, 136.1, 130.0, 129.4, 128.7, 128.5, 127.8, 127.6, 127.5, 127.4, 124.6, 122.1, 121.5, 118.9, 115.8, 114.0, 108.4, 63.9, 60.2, 58.2, 53.0, 52.2, 47.0, 43.3, 20.0, 12.8; IR (KBr) υ: 3452, 2951, 1742, 1709, 1604, 1572, 1496, 1457, 1435, 1381, 1352, 1307, 1253, 1218, 1184, 1161, 1119, 1088, 1019, 981, 810, 776 cm^-1^; MS (*m*/*z*): HRMS (ESI) Calcd. for C_35_H_33_N_2_O_7_ ([M+H]^+^): 593.2282. Found: 593.2285.


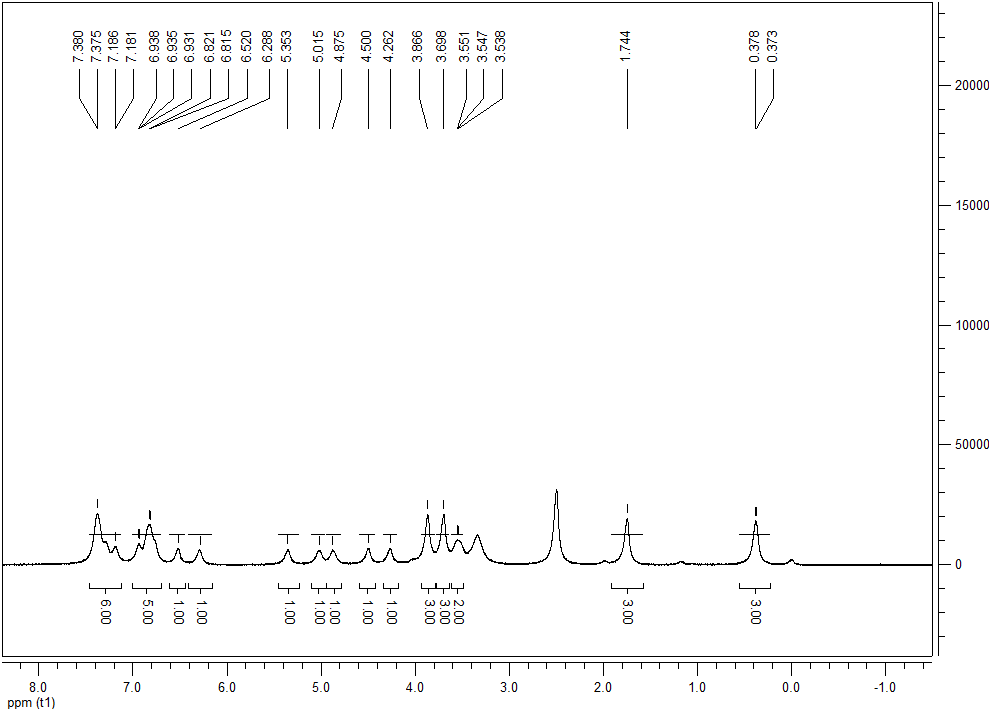


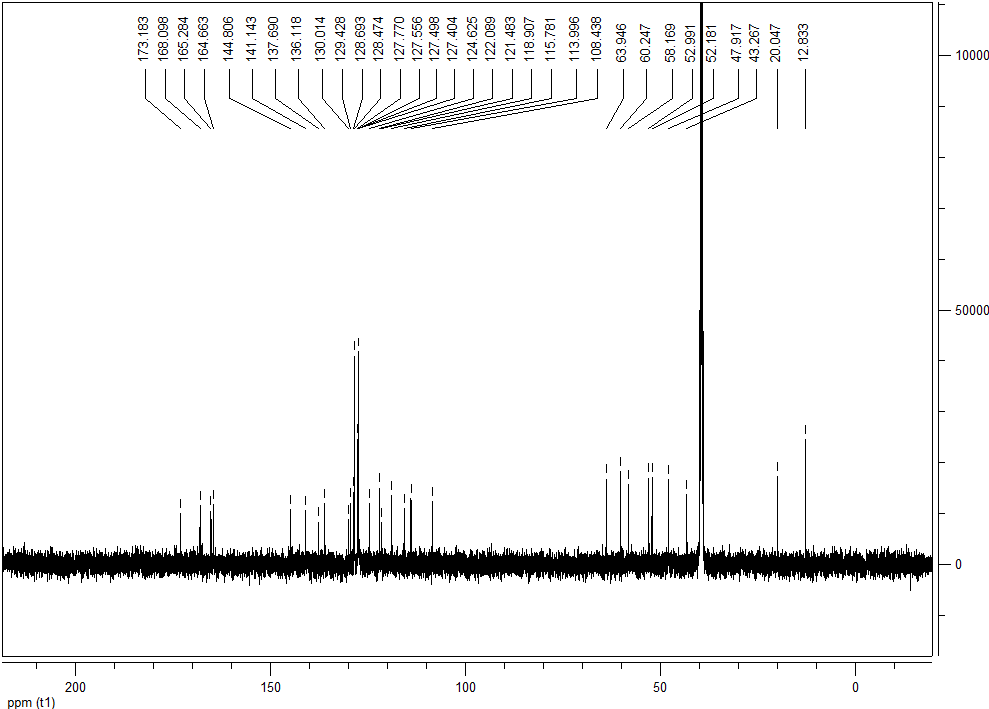


**3'-ethyl 1',2'-dimethyl 1-benzyl-2-oxo-3',4a'-dihydrospiro[indoline-3,4'-pyrido[1,2-a]quino-**

**line]-1',2',3'-tricarboxylate (3g)**: yellow solid, 63%, m.p. 176~177℃; ^1^H NMR (600 MHz, DMSO-*d_6_*) δ: 7.42~7.37 (m, 4H, ArH), 7.30 (brs, 1H, ArH), 7.17 (brs, 1H, ArH), 7.05 (brs, 1H, ArH), 6.92 (brs, 2H, ArH), 6.85~6.82 (m, 2H, ArH), 6.69 (brs, 1H, ArH), 6.52 (brs, 1H, ArH), 6.32 (brs, 1H, CH), 5.38 (brs, 1H, CH), 5.06 (d, *J* = 15.0Hz, 1H, CH), 4.90 (d, *J* = 15.0Hz, 1H, CH), 4.56 (s, 1H, CH), 4.28 (s, 1H, CH), 3.86 (s, 3H, OCH_3_), 3.70 (s, 3H, OCH_3_), 3.53 (brs, 1H, CH), 3.48 (brs, 1H, CH), 0.33 (brs, 3H, CH_3_); ^13^C NMR (150 MHz, DMSO-*d_6_*) δ: 173.4, 168.1, 165.3, 164.6, 145.0, 143.6, 137.3, 136.0, 129.6, 128.8, 128.5, 127.7, 127.6, 127.5, 126.5, 124.7, 122.3, 121.4, 121.1, 118.9, 115.3, 113.9, 109.0, 64.1, 60.3, 58.0, 53.0, 52.2, 48.0, 43.3, 12.8; IR (KBr) υ: 3452, 2950, 1740, 1707, 1610, 1571, 1493, 1463, 1435, 1384, 1357, 1307, 1282, 1252, 1221, 1177, 1132, 1087, 1020, 981, 902, 875, 831, 813, 783 cm^-1^; MS (*m*/*z*): HRMS (ESI) Calcd. for C_34_H_31_N_2_O_7_ ([M+H]^+^): 579.2126. Found: 579.2131.

**
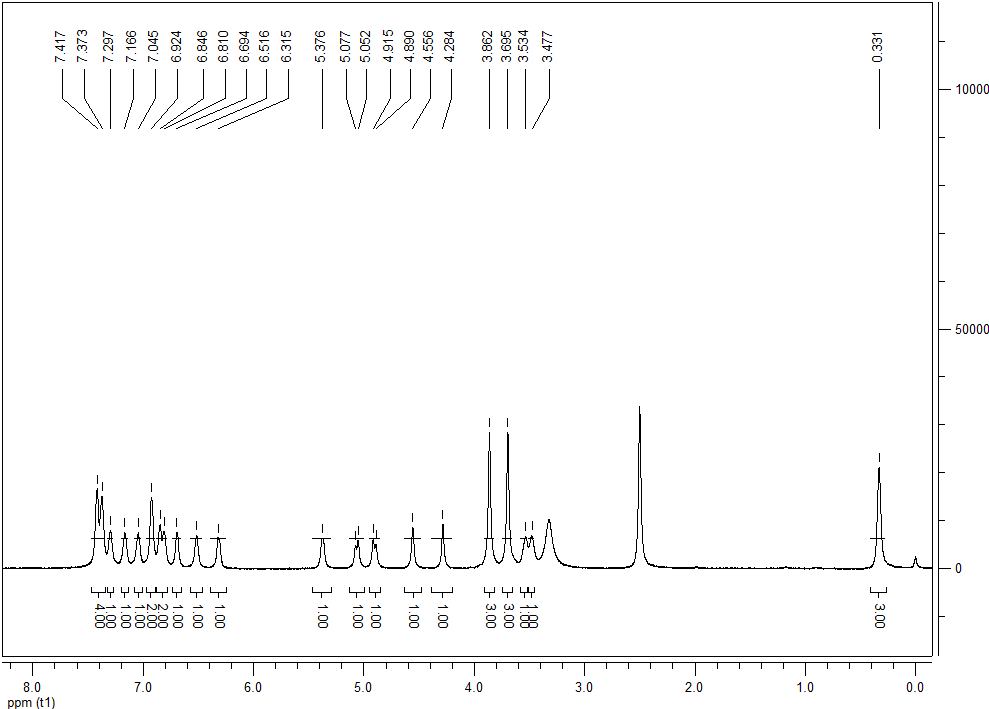
**

**
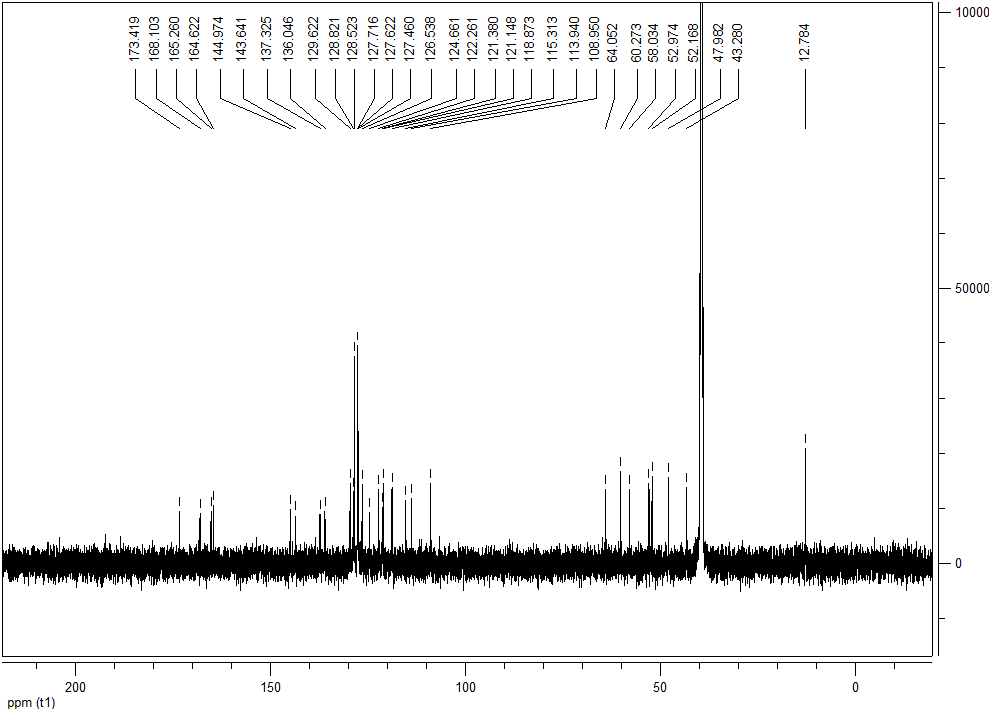
**

**3'-ethyl 1',2'-dimethyl 1-benzyl-5-chloro-2-oxo-3',4a'-dihydrospiro[indoline-3,4'-pyrido-**

**[1,2-a]quinoline]-1',2',3'-tricarboxylate (3h)**: yellow solid, 70%, m.p. 173~174℃; ^1^H NMR (600 MHz, DMSO-*d_6_*) δ: 7.39~7.36 (m, 4H, ArH), 7.31~7.30 (m, 1H, ArH), 7.20 (t, *J* = 7.8Hz, 1H, ArH), 7.13 (d, *J* = 8.4Hz, 1H, ArH), 6.95~6.90 (m, 3H, ArH), 6.84 (d, *J* = 7.2Hz, 1H, ArH), 6.60 (brs, 1H, ArH), 6.35 (d, *J* = 9.6Hz, 1H, CH), 5.37 (dd, *J_1_* = 9.6Hz, *J_2_* = 3.6Hz, 1H, CH), 5.07 (d, *J* = 15.6Hz, 1H, CH), 4.90 (d, *J* = 15.6Hz, 1H, CH), 4.58 (s, 1H, CH), 4.29 (s, 1H, CH), 3.87 (s, 3H, OCH_3_), 3.71 (s, 3H, OCH_3_), 3.61~3.56 (m, 2H, CH_2_), 0.42 (t, *J* = 7.2Hz, 3H, CH_3_); ^13^C NMR (150 MHz, DMSO-*d_6_*) δ: 172.9, 168.1, 165.2, 164.5, 144.6, 142.4, 137.3, 135.7, 129.8, 128.6, 128.0, 127.7, 127.6, 126.9, 126.5, 125.5, 122.4, 121.3, 118.5, 115.7, 113.7, 110.3, 63.7, 60.5, 58.3, 53.1, 52.3, 47.8, 43.4, 12.8; IR (KBr) υ: 3448, 2949, 1735, 1712, 1603, 1570, 1493, 1457, 1434, 1364, 1341, 1311, 1250, 1212, 1179, 1140, 1087, 1016, 977, 812, 775 cm^-1^; MS (*m*/*z*): HRMS (ESI) Calcd. for C_34_H_30_ClN_2_O_7_ ([M+H]^+^): 613.1736. Found: 613.1739.

**
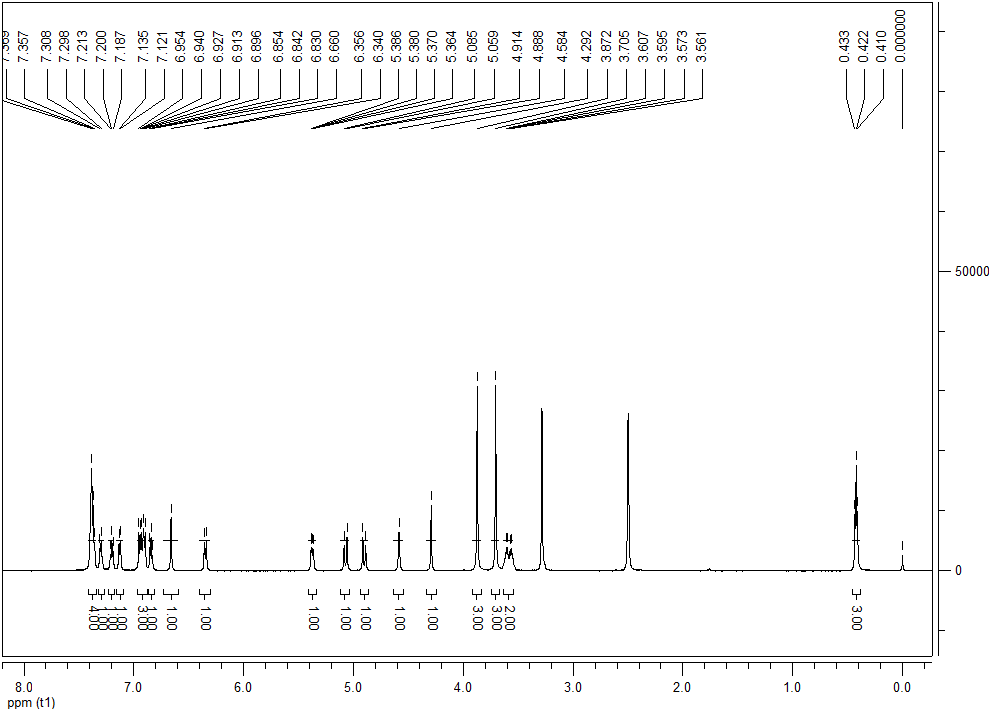
**

**
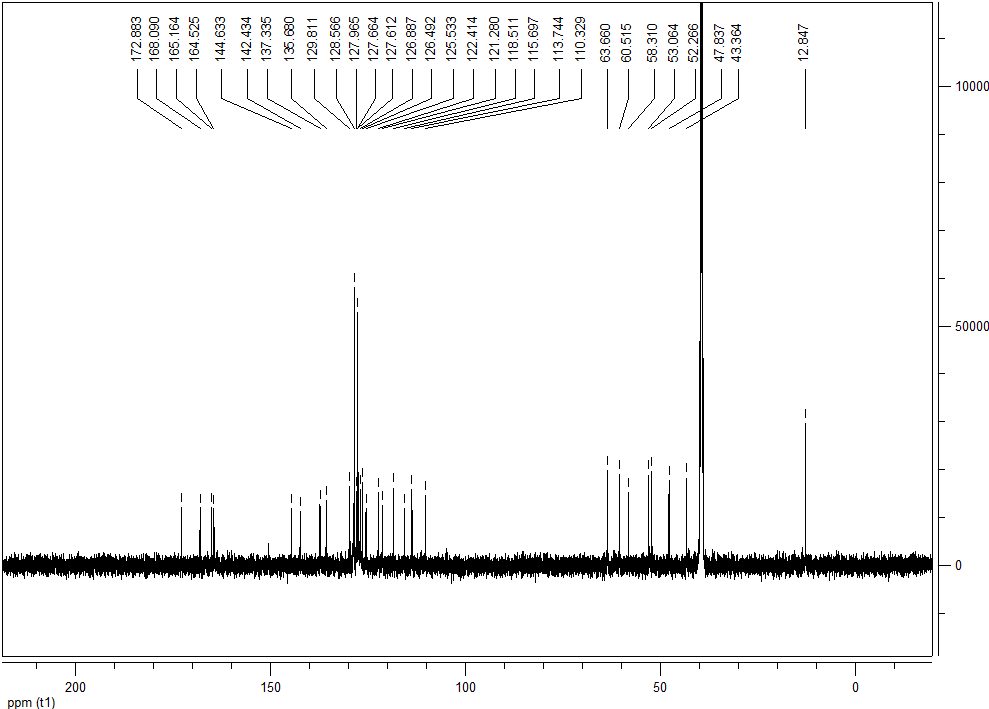
**

**3'-ethyl 1',2'-dimethyl 1-butyl-5-chloro-2-oxo-3',4a'-dihydrospiro[indoline-3,4'-pyrido[1,2-a]**

**quinoline]-1',2',3'-tricarboxylate (3i)**: yellow solid, 73%, m.p. 158~159℃; ^1^H NMR (600 MHz, DMSO-*d_6_*) δ: 7.20~7.18 (m, 2H, ArH), 7.05 (d, *J* = 7.8Hz, 1H, ArH), 6.92~6.91 (m, 2H, ArH), 6.86~6.85 (m, 1H, ArH), 6.65 (brs, 1H, ArH), 6.38 (d, *J* = 9.6Hz, 1H, CH), 5.37 (d, *J* = 6.6Hz, 1H, CH), 4.52 (s, 1H, CH), 4.21 (s, 1H, CH), 3.87 (s, 3H, OCH_3_), 3.74 (brs, 2H, CH_2_), 3.69 (s, 3H, OCH_3_), 3.63~3.62 (m, 2H, CH_2_), 1.57 (brs, 2H, CH_2_), 1.35~1.34 (m, 2H, CH_2_), 0.93 (brs, 3H, CH_3_), 0.61 (brs, 3H, CH_3_); ^13^C NMR (150 MHz, DMSO-*d_6_*) δ: 172.6, 172.6, 168.1, 165.2, 164.5, 144.6, 142.8, 137.4, 129.8, 128.6, 127.9, 127.6, 126.9, 126.6, 125.2, 122.4, 121.3, 118.5, 115.6, 113.8, 109.9, 63.5, 60.4, 58.2, 53.0, 52.2, 47.9, 29.0, 19.4, 13.5, 13.1; IR (KBr) υ: 3454, 2956, 1744, 1713, 1639, 1614, 1598, 1570, 1490, 1433, 1378, 1355, 1325, 1303, 1247, 1212, 1183, 1135, 1116, 1021, 989, 969, 944, 914, 868, 822, 780 cm^-1^; MS (*m*/*z*): HRMS (ESI) Calcd. for C_31_H_32_ClN_2_O_7_ ([M+H]^+^): 579.1893. Found: 579.1894.


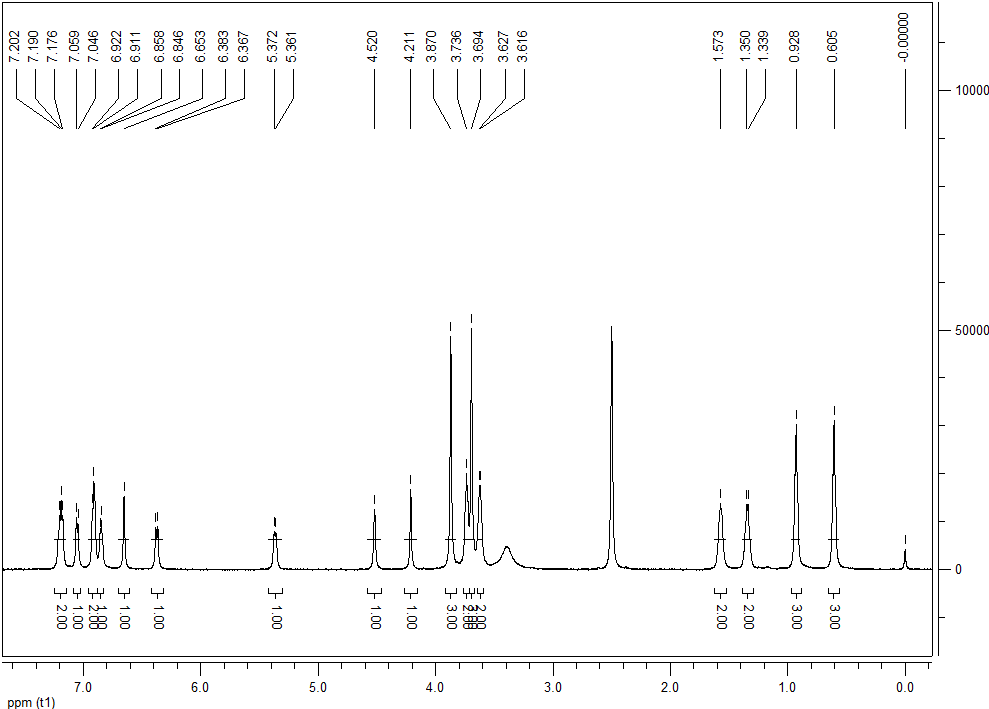


**
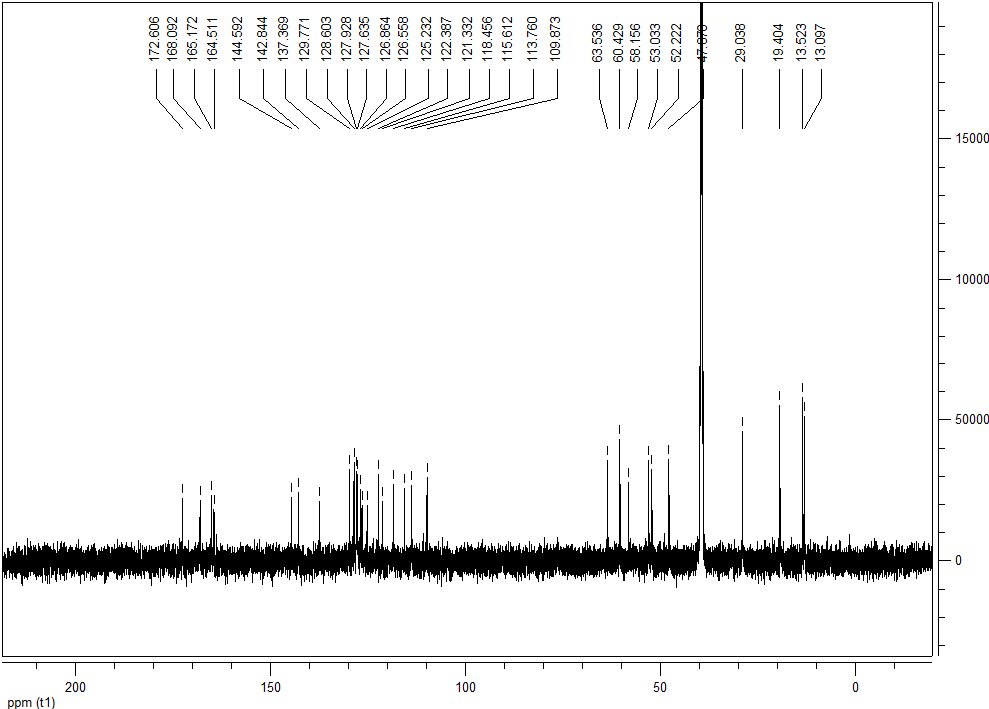
**

**3'-ethyl 1',2'-dimethyl 1-benzyl-5-fluoro-2-oxo-3',4a'-dihydrospiro[indoline-3,4'-pyrido-**

**[1,2-a]quinoline]-1',2',3'-tricarboxylate (3j)**: yellow solid, 65%, m.p. 160~163℃; ^1^H NMR (600 MHz, DMSO-*d_6_*) δ: 7.40~7.37 (m, 4H, ArH), 7.30 (brs, 1H, ArH), 7.20 (brs, 1H, ArH), 7.94~7.63 (m, 4H, ArH), 6.85 (brs, 1H, ArH), 6.43 (brs, 1H, ArH), 6.36 (d, *J* = 8.4Hz, 1H, CH), 5.40 (brs, 1H, CH), 5.08 (d, *J* = 15.6Hz, 1H, CH), 4.90 (d, *J* = 15.6Hz, 1H, CH), 4.60 (s, 1H, CH), 4.30 (s, 1H, CH), 3.87 (s, 3H, OCH_3_), 3.70 (s, 3H, OCH_3_), 3.59~3.55 (m, 2H, CH_2_), 0.38 (brs, 3H, CH_3_); ^13^C NMR (150 MHz, CDCl_3_) δ: 174.0, 168.6, 165.8, 165.1, 158.1 (d, *J* = 239.6Hz), 145.6, 139.7, 137.9, 135.3, 129.8, 128.8, 128.2, 128.0, 127.9, 127.6, 127.1 (d, *J* = 7.8Hz), 122.5, 121.6, 118.3, 115.9, 115.6 (d, *J* = 26.0Hz), 115.0 (d, *J* = 23.3Hz), 114.4, 109.0 (d, *J* = 8.0Hz), 64.5, 60.9, 59.3, 53.1, 52.2, 48.4, 44.4, 13.3; IR (KBr) υ: 3451, 2950, 1739, 1708, 1608, 1571, 1494, 1456, 1436, 1344, 1307, 1252, 1226, 1175, 1131, 1020, 979, 900, 875, 827, 775 cm^-1^; MS (*m*/*z*): HRMS (ESI) Calcd. for C_34_H_30_FN_2_O_7_ ([M+H]^+^): 597.2032. Found: 597.2034.

**
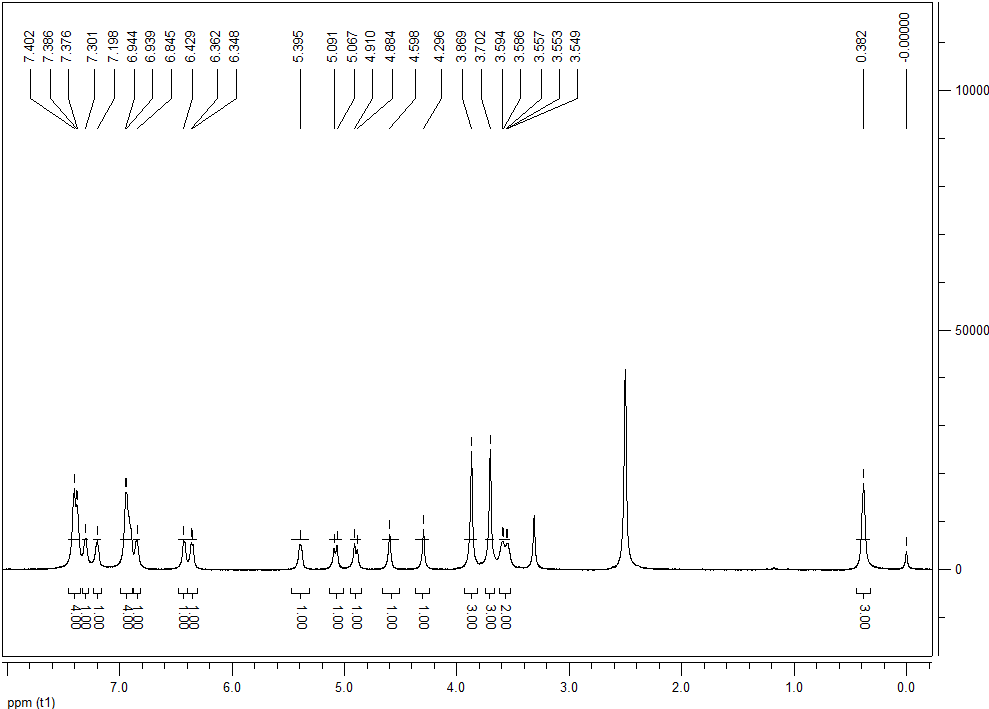
**

**
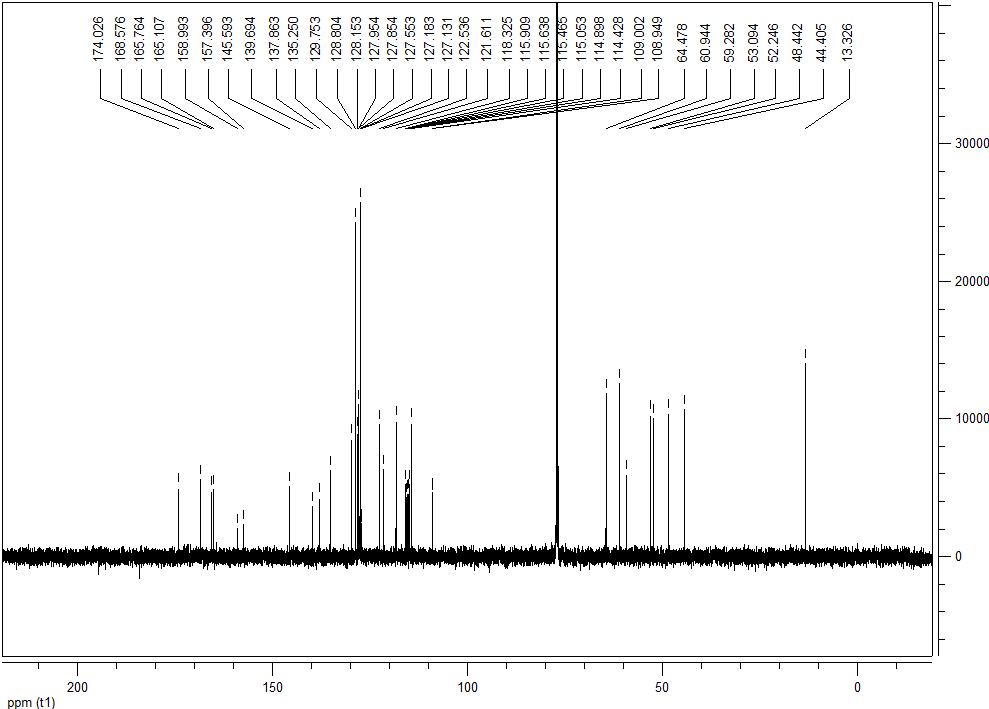
**

**spiro compounds** (**4a)**: white solid, 80%, m.p. 297~298℃; ^1^H NMR (600 MHz, DMSO-*d_6_*) δ: 7.60 (d, *J* = 8.4Hz, 2H, ArH), 7.44 (t, *J* = 7.8Hz, 2H, ArH), 7.38 (t, *J* = 7.2Hz, 1H, ArH), 7.19 (t, *J* = 7.2Hz, 1H, ArH), 7.14 (t, *J* = 7.2Hz, 2H, ArH), 7.05 (t, *J* = 8.4Hz, 1H, ArH), 6.99~6.97 (m, 6H, ArH), 6.79 (dd, *J_1_* = 9.0Hz, *J_2_* = 4.2Hz, 1H, ArH), 6.61~6.60 (m, 1H, ArH), 5.77 (d, *J* = 5.4Hz, 1H, CH), 5.20 (s, 1H, CH), 4.59 (brs, 2H, CH_2_), 4.32~4.31 (m, 1H, CH), 4.15 (s, 1H, CH), 4.00 (s, 3H, OCH_3_), 3.84 (s, 3H, OCH_3_), 3.54 (dd, *J_1_* = 7.5Hz, *J_2_* = 3.0Hz, 1H, CH), 3.48 (dd, *J_1_* = 7.5Hz, *J_2_* = 3.0Hz, 1H, CH), 3.34 (brs, 3H, OCH_3_), 2.66 (s, 1H, CH), 0.52 (s, 3H, CH_3_); ^13^C NMR (150 MHz, DMSO-*d_6_*) δ: 194.6, 175.1, 174.5, 174.1, 165.3, 164.5, 163.1, 157.7 (d, *J* = 236.4Hz), 146.5, 140.6, 138.5, 135.6, 131.8, 130.1, 129.8, 129.0, 128.6, 128.5, 127.5, 127.4, 126.6, 126.0 (d, *J* = 9.5Hz), 122.6, 117.2 (d, *J* = 24.8Hz), 114.6 (d, *J* = 23.6Hz), 113.5, 109.8 (d, *J* = 7.2Hz), 94.6, 60.7, 55.5, 53.3, 51.9, 50.8, 49.8, 45.9, 43.1, 42.2, 38.5, 18.9; IR (KBr) υ: 3450, 2951, 1778, 1711, 1670, 1590, 1488, 1459, 1434, 1376, 1345, 1324, 1244, 1176, 1123, 1033, 987, 950, 903, 843, 811 cm^-1^; MS (*m*/*z*): HRMS (ESI) Calcd. for C_46_H_39_FN_3_O_9_ ([M+H]^+^): 796.2665. Found: 796.2670.


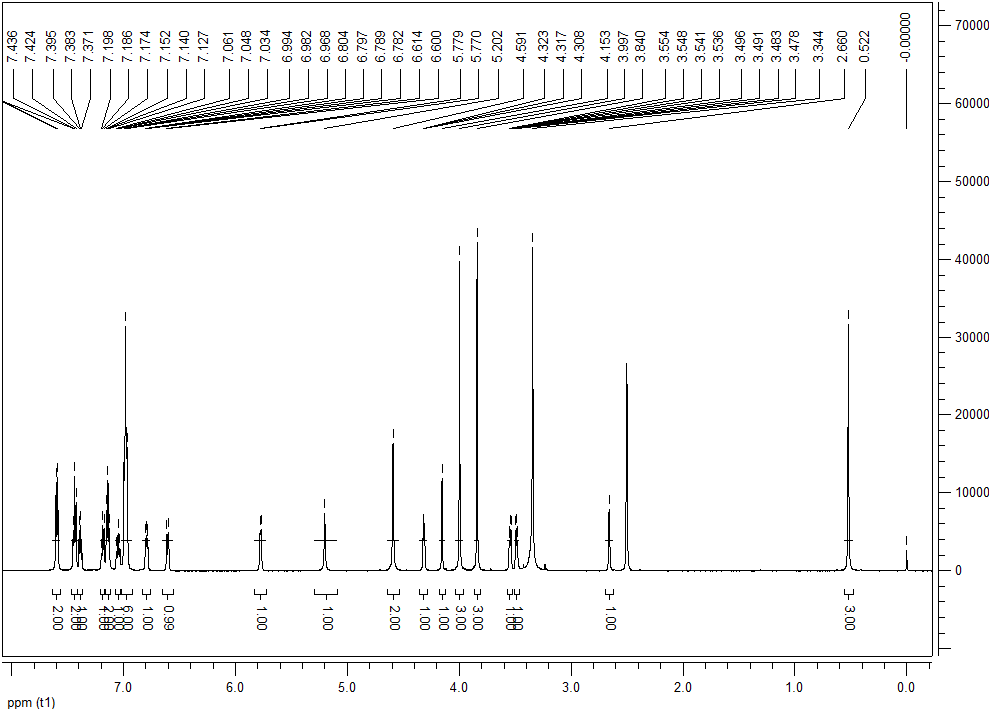


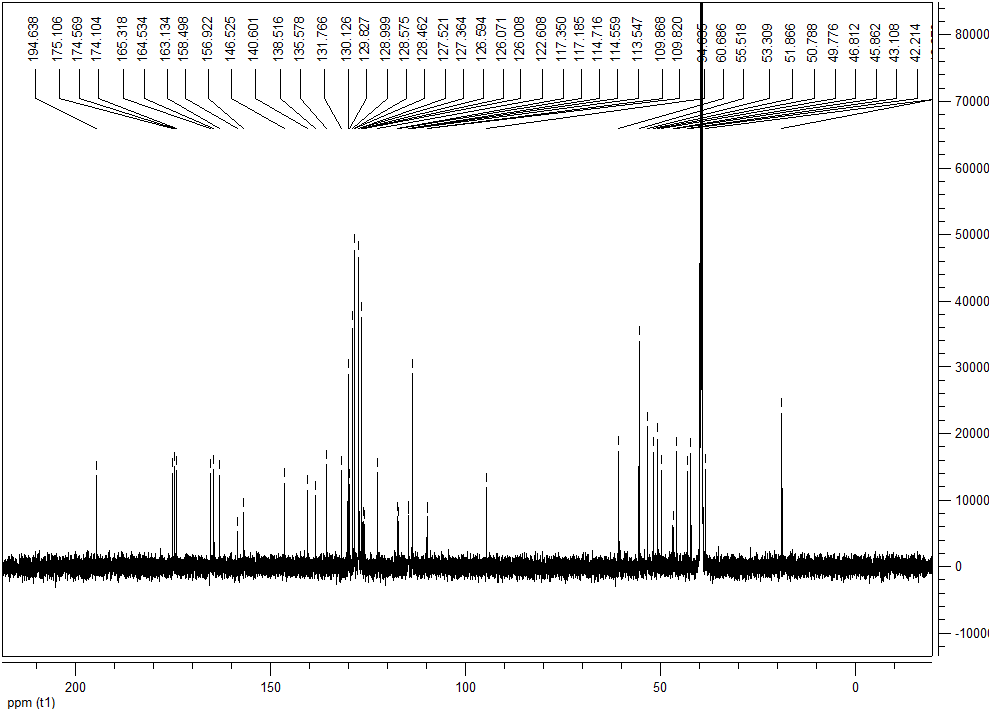


**spiro compounds** (**4b)**: white solid, 77%, m.p. >300℃; ^1^H NMR (600 MHz, DMSO-*d_6_*) δ: 7.49 (d, *J* = 7.8Hz, 2H, ArH), 7.43 (t, *J* = 7.8Hz, 2H, ArH), 7.38 (t, *J* = 7.8Hz, 1H, ArH), 7.26~7.25 (m, 3H, ArH), 7.19 (t, *J* = 7.2Hz, 1H, ArH), 7.14 (t, *J* = 7.2Hz, 2H, ArH), 7.00~6.99 (m, 4H, ArH), 6.83~6.82 (m, 2H, ArH), 5.74 (d, *J* = 5.4Hz, 1H, CH), 5.21 (s, 1H, CH), 4.54 (brs, 2H, CH_2_), 4.32~4.30 (m, 1H, CH), 4.15 (s, 1H, CH), 4.00 (s, 3H, OCH_3_), 3.54 (dd, *J_1_* = 7.5Hz, *J_2_* = 3.0Hz, 1H, CH), 3.47 (dd, *J_1_* = 7.5Hz, *J_2_* = 3.0Hz, 1H, CH), 3.35 (s, 3H, OCH_3_), 2.64 (s, 1H, CH), 2.37 (s, 3H, CH_3_), 0.50 (s, 3H, CH_3_); ^13^C NMR (150 MHz, CDCl_3_) δ: 196.0, 175.0, 174.7, 173.8, 166.0, 165.1, 146.8, 143.7, 141.7, 140.9, 135.0, 134.7, 131.3, 131.1, 129.3, 129.0, 128.9, 128.4, 128.3, 128.0, 127.4, 126.6, 126.1, 109.8, 96.5, 61.6, 53.7, 52.1, 51.3, 50.4, 46.0, 44.2, 42.6, 39.2, 21.6, 19.4; IR (KBr) υ: 3452, 2952, 1781, 1712, 1641, 1483, 1430, 1384, 1322, 1233, 1186, 1129, 949, 887, 803 cm^-1^; MS (*m*/*z*): HRMS (ESI) Calcd. for C_46_H_39_ClN_3_O_8_ ([M+H]^+^): 796.2420. Found: 796.2429.


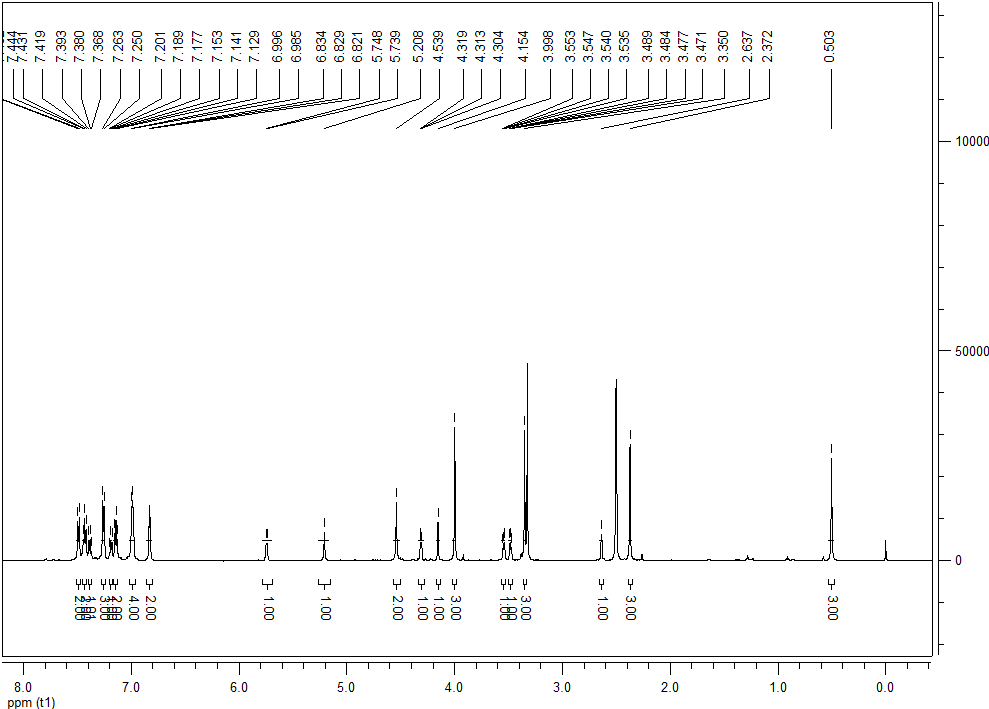


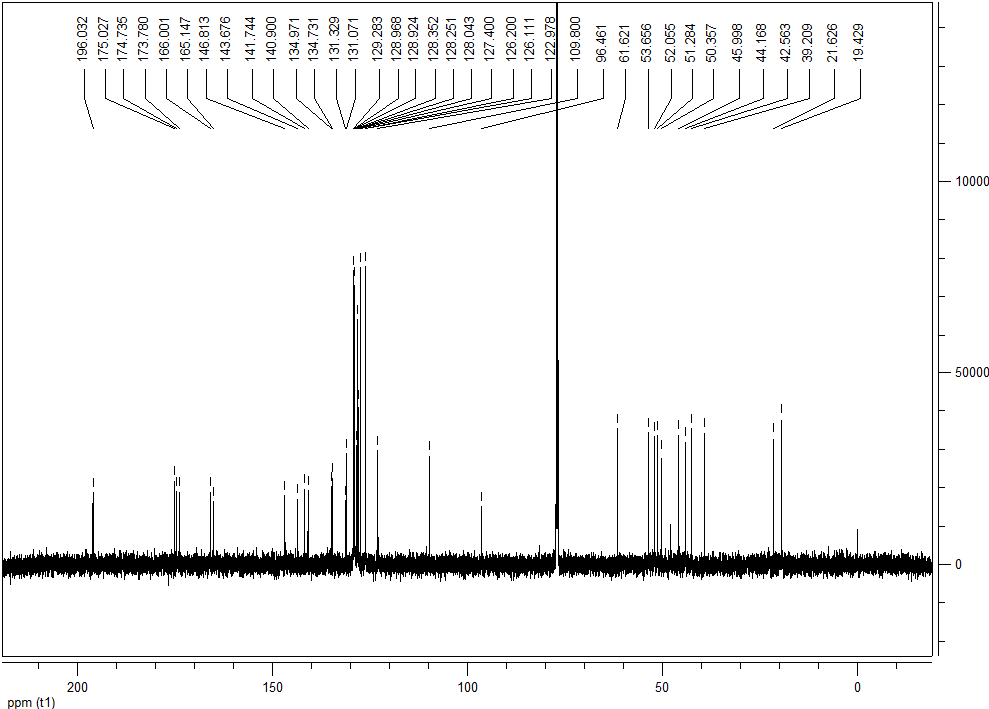


**spiro compounds** (**4c)**: white solid, 72%, m.p. 173~175℃; ^1^H NMR (600 MHz, DMSO-*d_6_*) δ: 7.46 (d, *J* = 8.4Hz, 2H, ArH), 7.22 (d, *J* = 7.8Hz, 2H, ArH), 7.09 (t, *J* = 8.4Hz, 1H, ArH), 6.91~6.86 (m, 5H, ArH), 6.57 (d, *J* = 6.6Hz, 1H, ArH), 5.81 (d, *J* = 4.2Hz, 1H, CH), 5.09 (s, 1H, CH), 4.31 (brs, 1H, CH), 4.04 (s, 1H, CH), 4.00 (s, 3H, OCH_3_), 3.80 (s, 3H, OCH_3_), 3.53~3.52 (m, 1H, CH), 3.46~3.45 (m, 1H, CH), 3.37 (brs, 1H, CH), 3.28 (s, 3H, OCH_3_), 3.26 (brs, 1H, CH), 2.61 (s, 1H, CH), 2.29 (s, 3H, CH_3_), 1.04~1.01 (m, 3H, CH), 0.84~0.82 (m, 1H, CH), 0.74~0.71 (m, 6H, CH_3_); ^13^C NMR (150 MHz, DMSO-*d_6_*) δ: 196.1, 173.7, 170.9, 169.5, 165.3, 164.5, 157.2 (d, *J* = 235.2Hz), 146.3, 143.3, 139.3, 134.6, 138.7, 127.7, 124.5 (d, *J* = 8.9Hz), 116.4 (d, *J* = 25.2Hz), 114.8 (d, *J* = 23.1Hz), 109.3 (d, *J* = 8.9Hz), 95.1, 93.6, 58.4, 55.1, 53.4, 51.6, 50.9, 49.9, 47.8, 47.5, 43.0, 37.5, 28.5, 21.0, 19.2, 13.5; IR (KBr) υ: 3457, 2956, 1712, 1600, 1514, 1489, 1455, 1386, 1322, 1237, 1178, 1131, 1025, 962, 808, 758 cm^-1^; MS (*m*/*z*): HRMS (ESI) Calcd. for C_44_H_42_FN_3_NaO_9_ ([M+Na]^+^): 798.2797. Found: 798.2783.

**
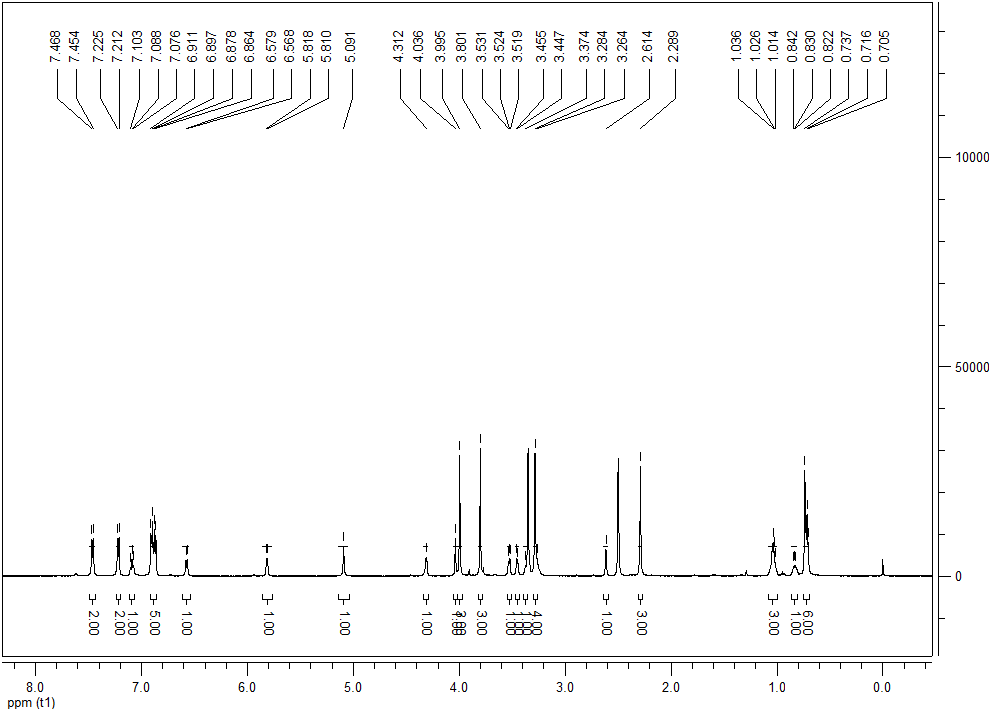
**

**
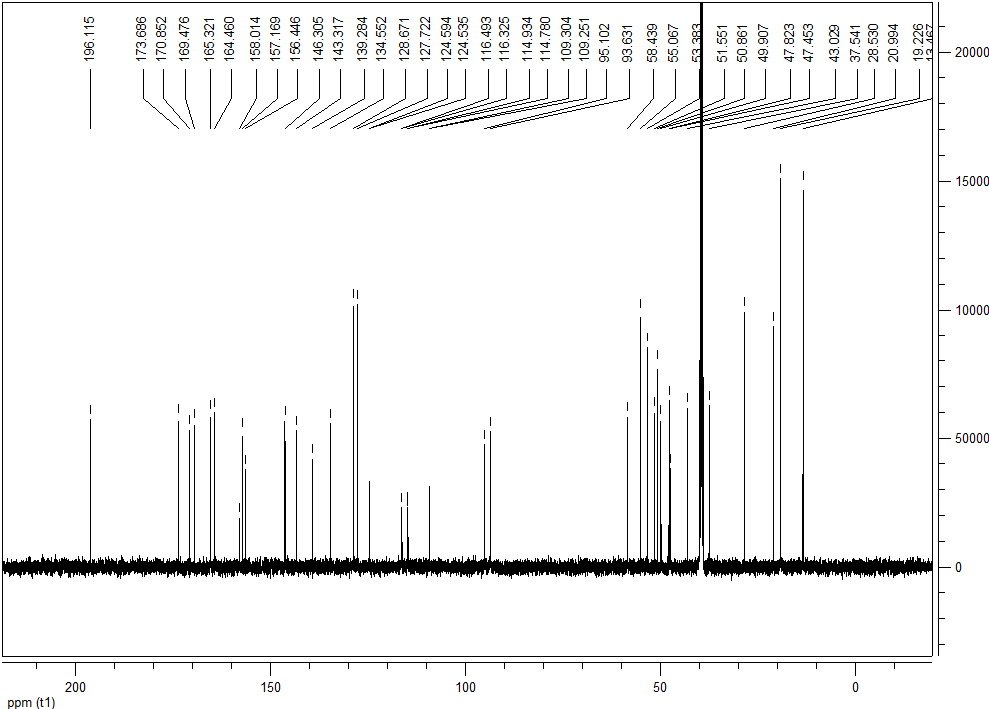
**

**spiro compounds** (**4d)**: white solid, 85%, m.p. 182~185℃; ^1^H NMR (600 MHz, DMSO-*d_6_*) δ: 7.51 (d, *J* = 8.4Hz, 2H, ArH), 7.46 (d, *J* = 8.4Hz, 2H, ArH), 7.11~7.08 (m, 1H, ArH), 7.05 (d, *J* = 8.4Hz, 2H, ArH), 6.91~6.90 (m, 3H, ArH), 6.57 (d, *J* = 8.4Hz, 1H, ArH), 5.82 (d, *J* = 1.8Hz, 1H, CH), 5.09 (s, 1H, CH), 4.32 (brs, 1H, CH), 4.06 (s, 1H, CH), 4.00 (s, 3H, OCH_3_), 3.80 (s, 3H, OCH_3_), 3.55~3.54 (m, 1H, CH), 3.48~3.47 (m, 1H, CH), 3.35 (brs, 5H, CH, OCH_3_), 2.62 (s, 1H, CH), 1.03 (brs, 3H, CH), 0.82 (brs, 1H, CH), 0.73~0.72 (m, 6H, CH_3_); ^13^C NMR (150 MHz, CDCl_3_) δ: 195.0, 174.7, 174.6, 173.6, 166.0, 165.1, 163.4, 158.5 (d, *J* = 239.9Hz), 146.6, 141.7, 138.8, 134.7, 130.4, 129.8, 129.4, 127.4, 126.2 (d, *J* = 8.6Hz), 123.0, 118.8 (d, *J* = 25.8Hz), 115.1 (d, *J* = 23.1Hz), 113.4, 108.4 (d, *J* = 8.4Hz), 96.6, 61.2, 55.4, 53.5, 51.9, 51.2, 50.5, 47.6, 46.0, 42.6, 40.2, 39.3, 29.0, 20.1, 19.7, 13.6; IR (KBr) υ: 3458, 2957, 1781, 1714, 1677, 1598, 1491, 1455, 1384, 1325, 1240, 1280, 1130, 1093, 1020, 960, 908, 868, 835, 807 cm^-1^; MS (*m*/*z*): HRMS (ESI) Calcd. for C_43_H_40_ClFN_3_O_9_ ([M+H]^+^): 796.2432. Found: 796.2432.

**
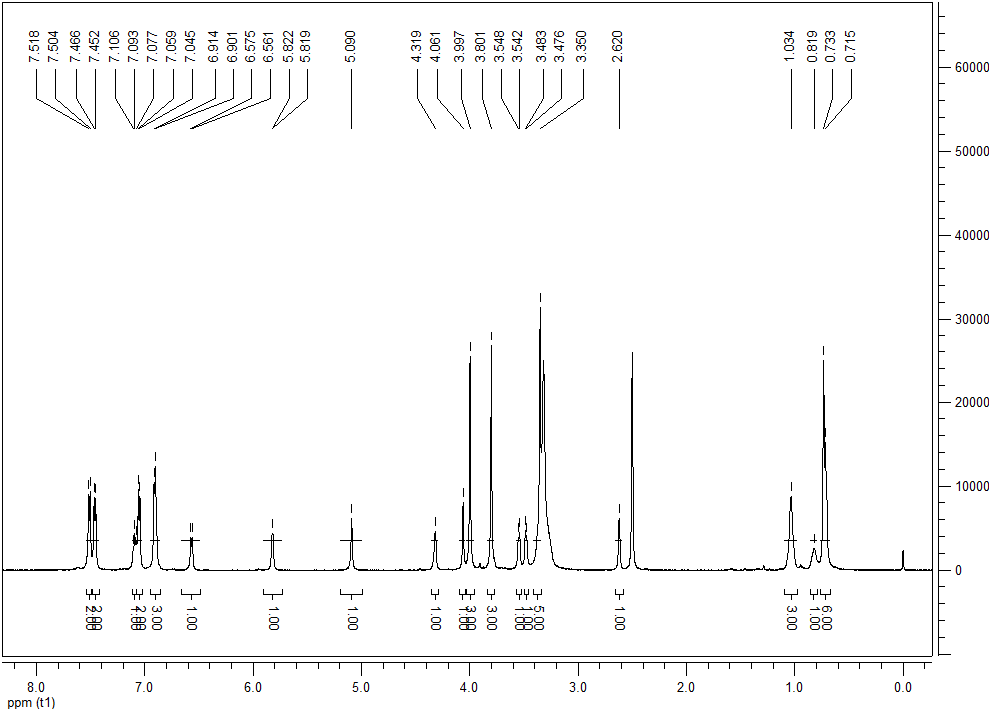
**

**
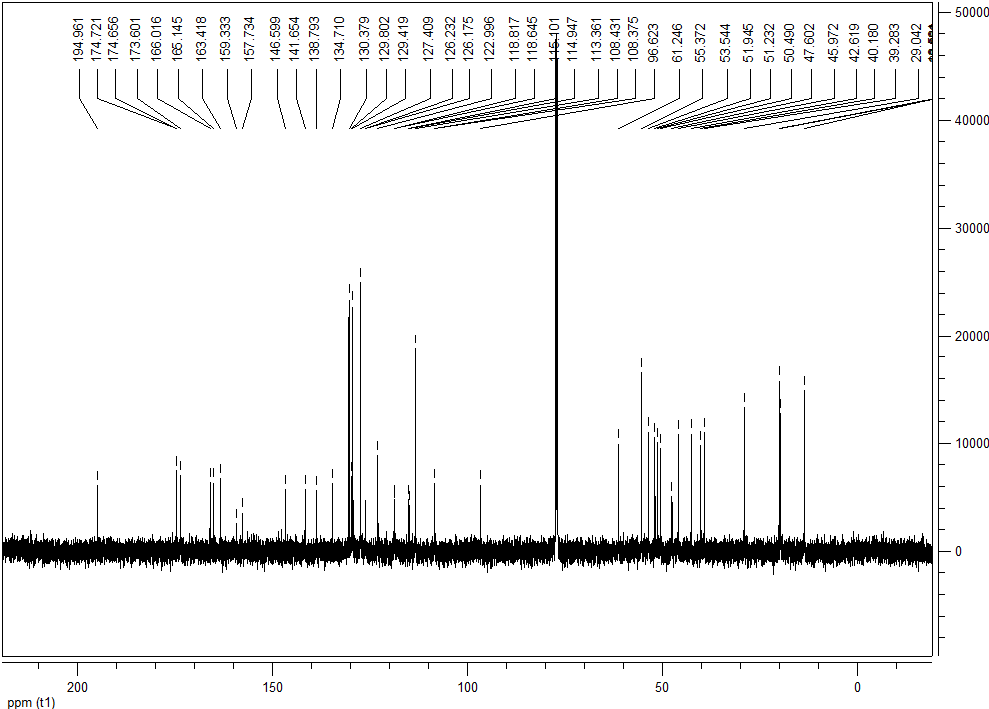
**

**spiro compounds** (**4e)**: white solid, 86%, m.p. 230~233℃; ^1^H NMR (600 MHz, DMSO-*d_6_*) δ: 7.53~7.49 (m, 4H, ArH), 7.09~7.06 (m, 1H, ArH), 7.04 (d, *J* = 8.4Hz, 2H, ArH), 6.92~6.90 (m, 3H, ArH), 6.60 (d, *J* = 8.4Hz, 1H, ArH), 5.13 (s, 1H, CH), 4.63 (d, *J* = 4.2Hz, 1H, CH), 4.05 (brs, 1H, CH), 4.02 (s, 3H, OCH_3_), 3.99 (s, 1H, CH), 3.80 (s, 3H, OCH_3_), 3.58~3.56 (m, 1H, CH), 3.48~3.43 (m, 1H, CH), 3.40~3.39 (m, 1H, CH), 3.36 (s, 3H, OCH_3_), 3.32 (brs, 1H, CH), 2.75 (s, 1H, CH), 1.46 (s, 3H, CH_3_), 1.03~1.01 (m, 1H, CH), 0.93~0.91 (m, 2H, CH), 0.80~0.79 (m, 1H, CH), 0.67 (d, *J* = 7.2Hz, 3H, CH_3_); ^13^C NMR (150 MHz, CDCl_3_) δ: 195.2, 175.1, 174.4, 173.4, 166.1, 165.2, 163.5, 158.3 (d, *J* = 240.2Hz), 146.6, 139.6, 138.0, 134.7, 130.6, 130.2, 129.8, 129.4, 127.3, 127.2 (d, *J* = 8.9Hz), 122.3, 117.4 (d, *J* = 25.5Hz), 115.0 (d, *J* = 23.4Hz), 113.4, 108.4 (d, *J* = 8.0Hz), 97.0, 61.8, 55.7, 55.4, 53.5, 51.3, 50.4, 46.6, 45.0, 42.7, 40.2, 34.6, 29.0, 20.0, 19.5, 13.6; IR (KBr) υ: 3457, 2958, 1747, 1712, 1601, 1492, 1446, 1426, 1384, 1355, 1318, 1264, 1236, 1175, 1138, 1022, 956, 918, 896, 876, 825, 803 cm^-1^; MS (*m*/*z*): HRMS (ESI) Calcd. for C_43_H_30_ClFN_3_O_9_ ([M+H]^+^): 796.2432. Found: 796.2429.

**
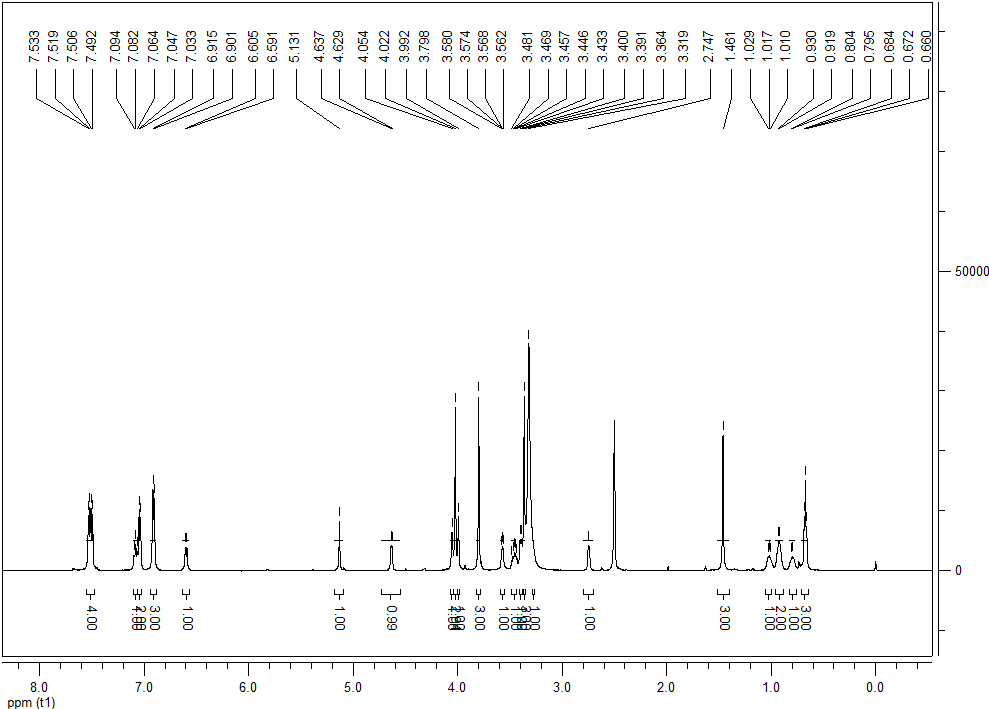
**

**
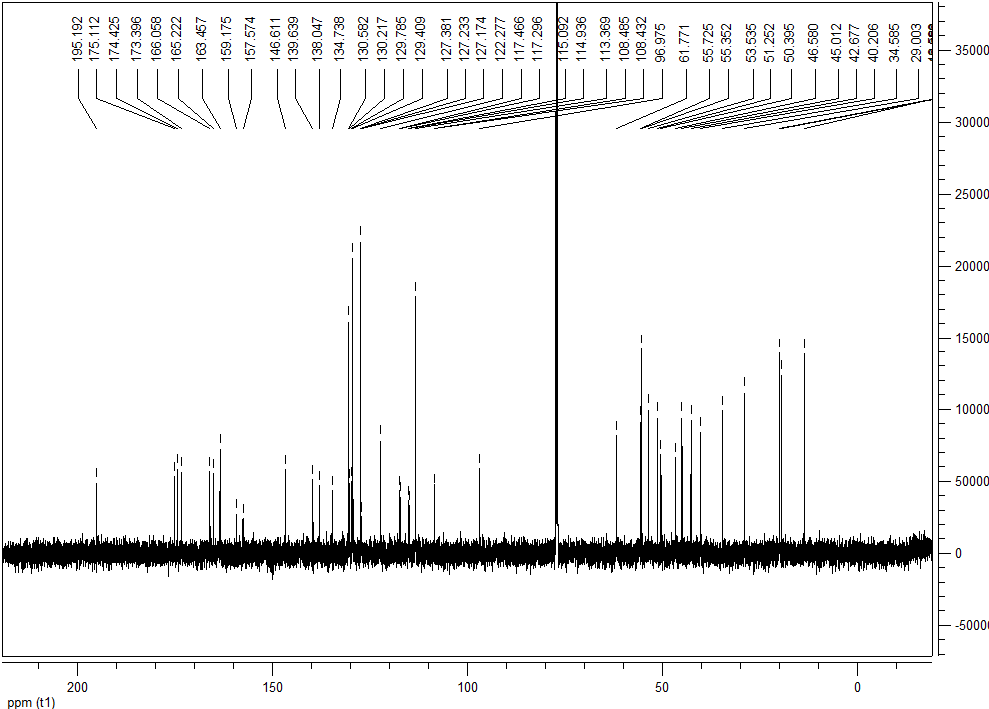
**

**spiro compounds** (**4f)**: white solid, 90%, m.p. >300℃; ^1^H NMR (600 MHz, DMSO-*d_6_*) δ: 7.43 (d, *J* = 4.8Hz, 2H, ArH), 7.27 (d, *J* = 7.2Hz, 1H, ArH), 6.90 (brs, 3H, ArH), 6.84 (brs, 1H, ArH), 5.50 (s, 1H, CH), 4.89 (d, *J* = 3.0Hz, 1H, CH), 4.47 (brs, 1H, CH), 4.00 (s, 3H, OCH_3_), 3.98 (s, 1H, CH), 3.79 (s, 3H, OCH_3_), 3.77~3.75 (m, 1H, CH), 3.69 (brs, 1H, CH), 3.36 (brs, 4H, CH, OCH_3_), 3.26~3.24 (m, 1H, CH), 2.74 (s, 3H, OCH_3_), 2.54 (s, 1H, CH), 1.00 (brs, 3H, CH), 0.82~0.78 (m, 1H, CH), 0.70 (brs, 3H, CH_3_); ^13^C NMR (150 MHz, CDCl_3_) δ:; IR (KBr) υ: 3453, 2957, 1861, 1779, 1699, 1675, 1641, 1584, 1511, 1483, 1455, 1433, 1368, 1342, 1321, 1269, 1232, 1203, 1170, 1129, 1086, 1049, 1017, 972, 942, 924, 879, 841, 811 cm^-1^; MS (*m*/*z*): HRMS (ESI) Calcd. for C_37_H_35_ClN_2_NaO_11_ ([M+Na]^+^): 741.1822. Found: 741.1808.

**
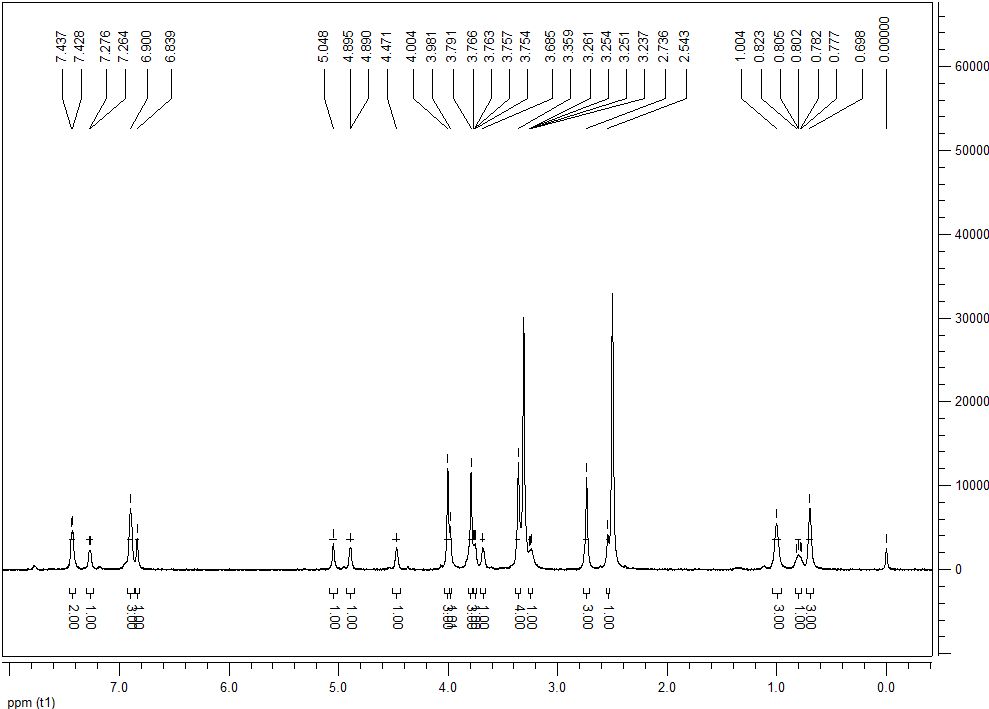
**

**spiro compounds** (**4g)**: white solid, 88%, m.p. 283~285℃; ^1^H NMR (600 MHz, DMSO-*d_6_*) δ: 7.30 (d, *J* = 8.4Hz, 2H, ArH), 7.17 (d, *J* = 7.8Hz, 2H, ArH), 7.08 (td, *J_1_* = 9.3Hz, *J_2_* = 2.4Hz, 1H, ArH), 6.88 (dd, *J_1_* = 8.4Hz, *J_2_* = 4.2Hz, 1H, ArH), 6.61 (dd, *J_1_* = 9.3Hz, *J_2_* = 2.4Hz, 1H, ArH), 5.06 (s, 1H, CH), 4.92 (dd, *J_1_* = 6.6Hz, *J_2_* = 2.4Hz, 1H, CH), 4.48 (dd, *J_1_* = 6.6Hz, *J_2_* = 3.6Hz, 1H, CH), 4.00~3.99 (m, 4H, CH, OCH_3_), 3.76 (dd, *J_1_* = 7.5Hz, *J_2_* = 3.6Hz, 1H, CH), 3.67 (dd, *J_1_* = 7.5Hz, *J_2_* = 3.6Hz, 1H, CH), 3.36 (s, 3H, OCH_3_), 3.28~3.27 (m, 1H, CH), 3.20~3.19 (m, 1H, CH), 2.71 (s, 3H, OCH_3_), 2.56 (s, 1H, CH), 2.31 (s, 3H, CH_3_), 1.02 (brs, 3H, CH), 0.81~0.79 (m, 1H, CH), 0.71 (d, *J* = 7.2Hz, 3H, CH_3_); ^13^C NMR (150 MHz, DMSO-*d_6_*) δ: 194.8, 175.3, 174.2, 174.1, 165.4, 164.6, 163.1, 157.5 (d, *J* = 236.1Hz), 146.5, 140.6, 139.0, 138.2, 130.0, 129.8, 129.4, 129.1, 126.3, 125.9 (d, *J* = 8.3Hz), 122.7, 117.3 (d, *J* = 22.5Hz), 114.7 (d, *J* = 23.3Hz), 113.4, 109.3, 94.5, 60.2, 55.4, 53.3, 51.8, 50.8, 49.7, 47.0, 45.8, 42.1, 28.7, 20.6, 19.4, 19.2, 13.4; IR (KBr) υ: 3456, 2956, 1864, 1780, 1740, 1697, 1641, 1585, 1489, 1455, 1435, 1382, 1339, 1320, 1267, 1232, 1202, 1177, 1120, 1086, 1046, 1017, 979, 929, 872, 816, 792 cm^-1^; MS (*m*/*z*): HRMS (ESI) Calcd. for C_37_H_35_FN_2_NaO_10_ ([M+Na]^+^): 709.2168. Found: 709.2158.

**
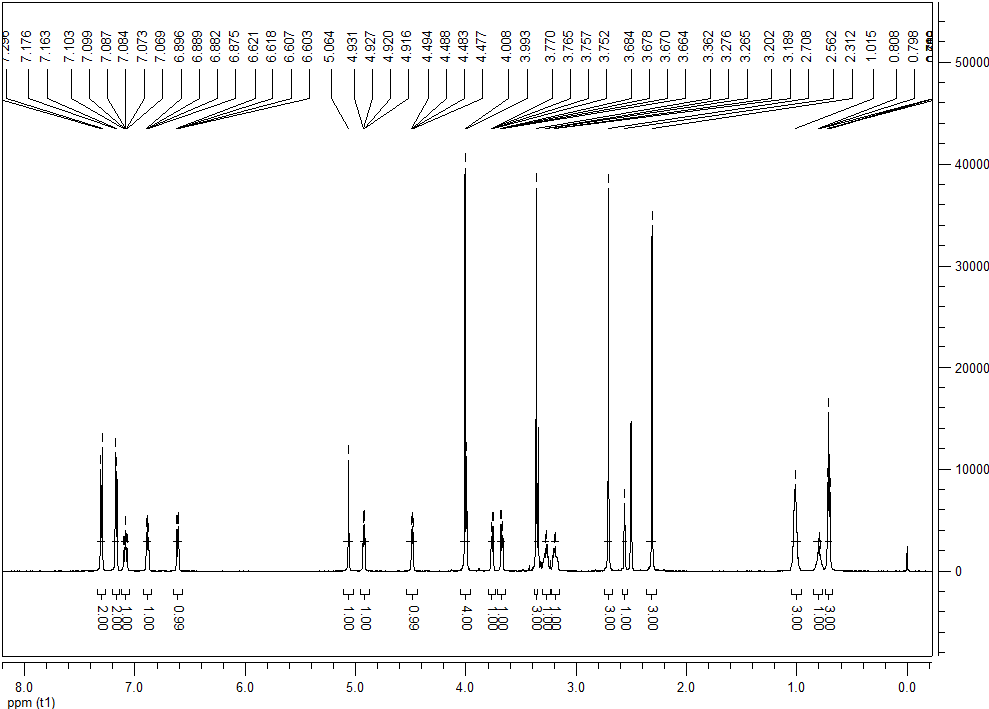
**


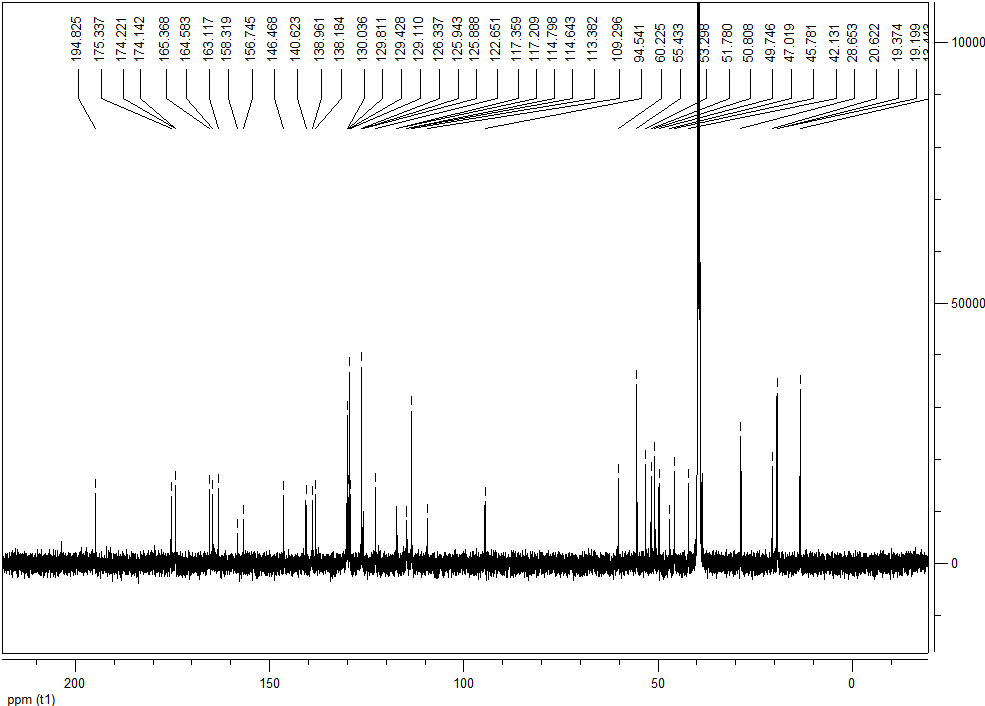

Supplement: Supplementary file 1 — Supplementary material 1 (doc 4383 KB) [file 11030_2013_9459_MOESM1_ESM.doc]
